# Nucleophilic Fluorination Catalyzed by a Cyclometallated Rhodium Complex

**DOI:** 10.1021/acs.organomet.2c00052

**Published:** 2022-03-31

**Authors:** Patrick J. Morgan, Graham C. Saunders, Stuart A. Macgregor, Andrew C. Marr, Peter Licence

**Affiliations:** † GSK Carbon Neutral Laboratory, School of Chemistry, University of Nottingham, Nottingham NG7 2TU, U.K.; ‡ School of Science, University of Waikato, Hamilton 3240, New Zealand; § School of Engineering and Physical Sciences, Heriot-Watt University, William H. Perkin Building, Edinburgh EH14 4AS, U.K.; ∥ School of Chemistry and Chemical Engineering, Queen’s University Belfast, David Keir Building, Belfast BT9 5AG, U.K.

## Abstract

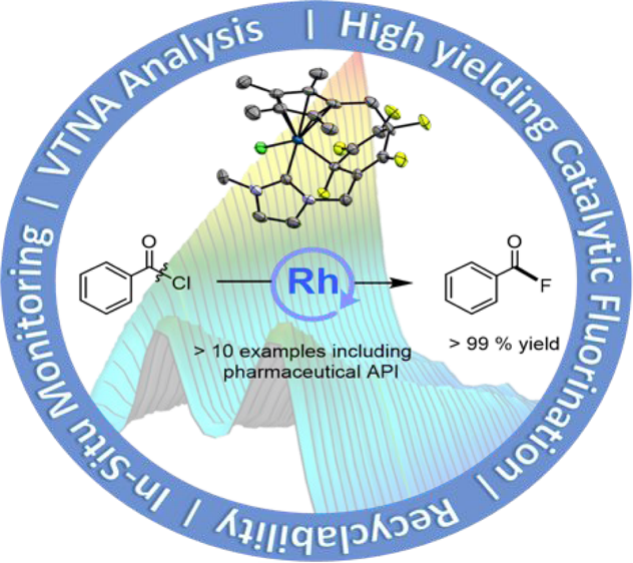

Quantitative catalytic nucleophilic fluorination of a range of acyl chlorides to acyl fluorides was promoted by a cyclometallated rhodium complex [(η^5^,κ_2_C-C_5_Me_4_CH_2_C_6_F_5_CH_2_NC_3_H_2_NMe)- RhCl] (**1**). **1** can be prepared in high yields from commercially available starting materials using a one-pot method. The catalyst could be separated, regenerated, and reused. Rapid quantitative fluorination generated the fluoride analogue of the active pharmaceutical ingredient probenecid. Infrared in situ monitoring verified the clean conversion of the substrates to products. VTNA graphical kinetic analysis and DFT calculations lead to a postulated reaction mechanism involving a nucleophilic Rh–F bond.

## Introduction

Fluorinated organic compounds are of unique strategic importance to the pharmaceutical industry. Thirty-seven percent of all small molecule active pharmaceutical ingredients approved by the FDA in 2020 contained at least one fluorine moiety. Fluorine-containing pharmaceuticals have been increasingly targeted in the past decade and represented 26% of all pharmaceuticals approved by the FDA between 2011 and 2020.^1^ Synthetic methods are available for the inclusion of fluorine within these pharmaceutical targets; however, these methods often require the use of highly activated fluorination reagents, such as NFSI and Selectfluor.^2^ As we drive toward more sustainable and less-toxic synthesis, such reactive reagents become increasingly unsuitable due to their superstoichiometric use, poor atom efficiency, and the associated embedded energy and waste related to their synthesis over their life cycle. It is therefore vital to develop clean catalytic methodologies for the synthesis of fluorinated compounds.

Nucleophilic fluorination represents the state of the art in fluorination, with significant focus in the past decade on expanding the scope of synthetic methods by accessing new sites of reactivity.^3^ Acyl fluorides are key targets and intermediates in nucleophilic fluorinations, as they have important synthetic utility and exhibit improved stability and interesting reactivity compared to more commercially accessible chloride analogues. Applications have been demonstrated in the generation of nucleophilic fluorine,^4^ cross-coupling reactions,^5−11^ enantioselective transformations,^12,13^ and the trifluoromethylation of aryl species.^14^ Traditional syntheses of acyl fluorides require toxic reagents (such as the SeF_4_/pyridine complex^15^ or cyanuric fluoride^16^) and harsh reaction conditions and typically suffer from poor functional group compatibility. Recently, new protocols for acyl fluoride synthesis have emerged,^17^ including the deoxyfluorination of carboxylic acids,^18−22^ direct fluorination with (Me_4_N)SCF_3_,^23^ acyl/fluorine transfer,^5,24−27^ and transition metal catalysis.^28^

**Scheme 1:**
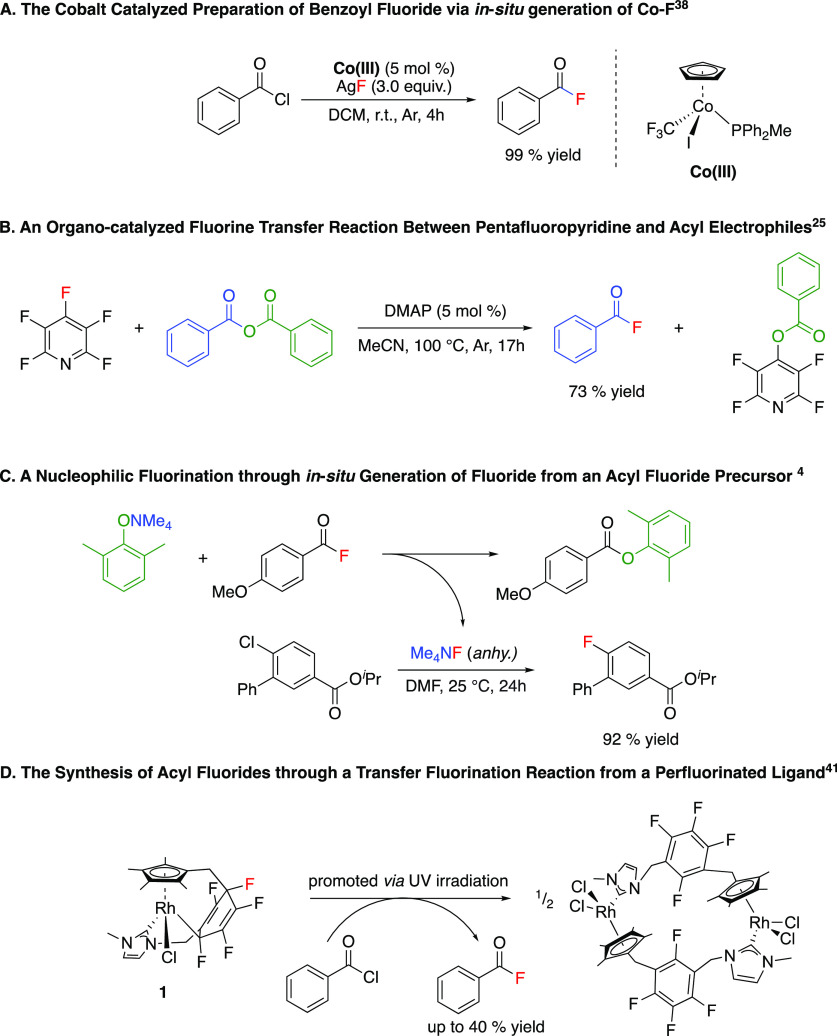
Recent Literature Examples of Fluorination Reactions

One of the most successful strategies for nucleophilic fluorination involves the formation of a nucleophilic fluorine attached to a transition metal.^29−32^ Treatment of metal complexes with fluoride salts, such as KF, CsF, and AgF, can result in the formation of transition metal–fluorine bonds, either in isolated complexes^33−38^ or in situ,^39^ and these complexes have been shown to promote nucleophilic fluorination. Fluoride salts provide a cheap and abundant source of fluorine, and compared to stoichiometric fluorinating reagents, the use of fluoride salts represents a more atom economical approach to the introduction of a fluorine atom into a molecule. In 2015, Gray and co-workers demonstrated the fluorination of acyl chlorides through the stoichiometric addition of an iridium–fluoride complex, highlighting the capability of transition metal fluoride complexes to undergo fluorination upon treatment with organic electrophiles.^36^ Baker and co-workers developed a similar approach to the synthesis of acyl fluorides, via the stoichiometric addition of cobalt transition metal fluoride complexes with acyl chloride substrates. A catalytic protocol was then developed based on Co(III) with AgF acting as the fluorine source, with a metal loading of 5 mol % (Scheme 1A). In the proposed qualitative mechanism, it was suggested that a nucleophilic Co–F bond was generated in situ by the reaction of a Co–I bond with AgF.^39^

Nucleophilic fluorination can also be achieved without the formation of a transition metal fluoride at the point of fluorination. Crimmin and co-workers reported a fluorine transfer reaction, where nucleophilic fluorine, generated through the treatment of pentafluoropyridine with 4-dimethylamino pyridine, resulted in the fluorination of acyl electrophiles through the generation of a nucleophilic fluoride salt in situ (Scheme 1B).^24^ Gouverneur and co-workers demonstrated a nucleophilic fluorination methodology utilizing a hydrogen-bonding phase transfer catalyst capable of solubilizing and improving the reactivity of metal fluoride salts such as KF.^40^ This asymmetric fluorination enabled selective fluorination, accessing a range of enantiopure β-fluoroamines using a superstoichiometric excess of the metal fluoride salt. Sanford and co-workers developed S_N_Ar nucleophilic fluorinations, targeting the enhancement of the reactivity of metal fluoride salts, or employing acyl fluorides to generate nucleophilic fluoride in situ (Scheme 1C).^4,41^ Recently, we reported the unexpected generation of a nucleophilic carbon–fluorine bond within the perfluorinated group of a ligand (Scheme 1D).^42^ Treatment of the cyclometallated Rh complex (**1**) with an organic electrophile led to the formation of a fluorinated organic product by fluorine transfer.

**Figure 1:**
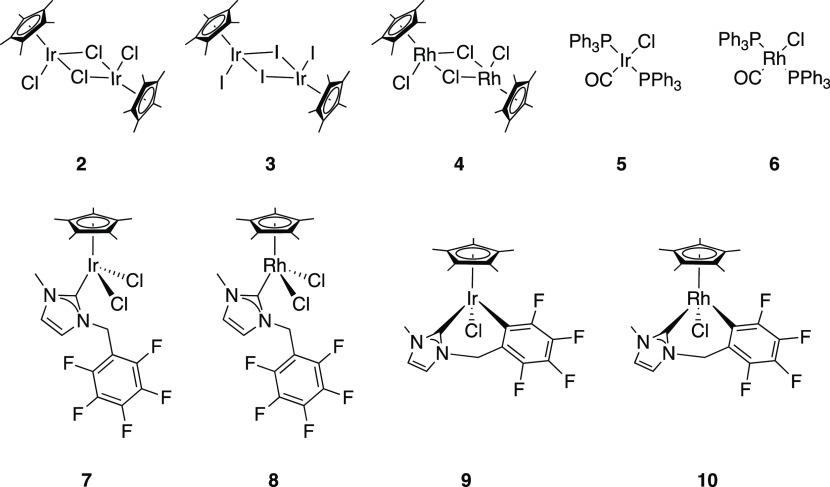
Structure of catalysts tested for the transition-metal-catalyzed fluorination of acyl chlorides as described in Table 1.

**Scheme 2:**
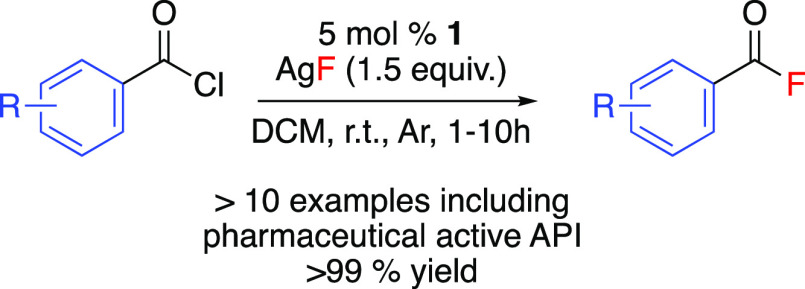
Rhodium-Catalyzed Nucleophilic Fluorination of Acyl Chlorides

In order to search for robust and selective catalytic methods for fluorination that avoid the use of expensive superstoichiometric additives and obscure reagents, the catalytic activity of Rh and Ir complexes was assessed, including that of previously reported cyclometallated complexes [Cp*IrCl(κC_2_-MeNC_3_H_2_NCH_2_C_6_F_4_)] (**9**),^43^ [Cp*RhCl(κC_2_-MeNC_3_H_2_NCH_2_C_6_F_4_)] (**10**) (see Figure 1), and [(η^5^,κ_2_C-C_5_Me_4_CH_2_C_6_F_5_CH_2_NC_3_H_2_NMe)-RhCl] (**1**).^44^ Herein, we report the activity of **1** in the catalytic nucleophilic fluorination of acyl chlorides affording fluorinated products in excellent yields (Scheme 2).

This observation poses the following questions: does the nucleophilic fluorination proceed by M–F bond formation, as proposed by Baker and co-workers for Co(III)?^28,39^ Or does it involve transfer of fluorine from the fluorinated ligand itself in **1**?^42^ A spectroscopic and computational investigation leads to the proposal of a mechanism involving the formation of a new Rh–F bond.

## Results and Discussion

### Catalyst Screening

Following the recent discovery of [Cp*IrCl(κC^2^-MeNC_3_H_2_NCH_2_C_6_F_4_)], **9**,^43^ and [(η^5^,*κ*^2^C-C_5_Me_4_CH_2_C_6_F_5_CH_2_NC_3_H_2_NMe)-RhCl], **1**,^44^ the activity of these complexes toward catalytic fluorination was evaluated. Toluoyl chloride was chosen as a common model substrate for catalytic fluorinations employing group 9 organometallic complexes.^36,39^ Silver fluoride was utilized as the source of fluorine, as silver has been shown to have interesting and complementary reactivity with Rh and Ir organometallic complexes^43,44^ and to act as a source of fluoride to Co(III).^28,39^ In all cases, the yield of the resultant toluoyl fluoride product was calculated as a contained yield by ^19^F NMR, against an internal standard. Activity was benchmarked against well-established iridium and rhodium complexes. Initially, the “parent” pentamethyl cyclopentadienyl complexes [IrCp*Cl_2_]_2_, **2**, [IrCp*I_2_]_2_, **3**, and [RhCp*Cl_2_]_2_, **4**, were tested as catalysts; these gave poor to moderate yields of the fluorinated product (Table 1, entries 2/3/4). The rhodium complex **4** (Table 1, entry 4) promoted greater fluorination than the iridium analogue, **2** (entry 2), whereas substituting the iridium chloride for iodide (**3**, entry 3) improved the yield of the fluorinated product from 9 to 40%. Vaska’s complex, **5**, and Rh analogue **6** exhibited a similar reactivity, with improved fluorination capability of **6** over **5** (Table 1, entry 6/5, respectively). Reacting the substrate in the presence of catalytic quantities of N-heterocyclic carbene complexes of Cp*Ir and Rh **7** and **8** (Table 1, entry 7/8, respectively) resulted in minor conversion to the fluorinated product and demonstrated similar yield to the noncatalyzed control reaction (Table 1, entry 1). Reaction with the orthometallated complexes **9** and **10** (Table 1, entry 9/10, respectively) resulted in 16% contained yield for both, a slightly higher degree of fluorination than the nonorthometallated complexes **7** and **8**. The performance of cyclometallated complex **1** proved to be significantly better than that of other complexes screened, affording 88% contained yield (Table 1, entry 11).

**Table 1:**
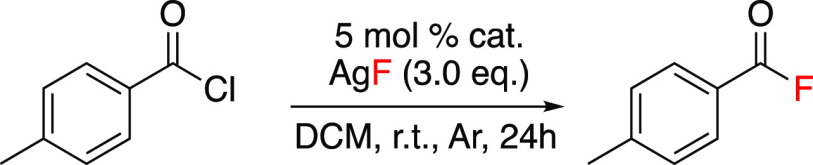
Catalyst Screen for the Fluorination of Toluoyl Fluoride with AgF^a^

entry	catalyst	contained yield (%)^b^
1	none	2
2	[IrCp*Cl_2_]_2_**(2)**	9
3	[IrCp*I_2_]_2_**(3)**	40
4	[RhCp*Cl_2_]_2_**(4)**	24
5	IrCl(CO)(PPh_3_)_2_**(5)**	16
6	RhCl(CO)(PPh_3_)_2_**(6)**	26
7	IrCp*Cl_2_(F_5_Bzmim) **(7)**	4
8	RhCp*Cl_2_(F_5_Bzmim) **(8)**	5
9	[Cp*IrCl(κC_2_-MeNC_3_H_2_NCH_2_C_6_F_4_)] **(9)**	16
10	[Cp*RhCl(κC_2_-MeNC_3_H_2_NCH_2_C_6_F_4_)] **(10)**	16
11	[(*η*^5^,*κ*_2_C-C_5_Me_4_CH_2_C_6_F_5_CH_2_NC_3_H_2_NMe)- RhCl] **(1)**	88

a Reaction conditions: Toluoyl chloride (1 mmol), silver fluoride (3.0 mmol), catalyst (5 mol %), DCM (5 mL), 400 rpm stirring, argon, room temperature, 24 h.

b ^19^F NMR yields determined against the internal standard of α,α,α-trifluorotoluene (0.163 mmol).

The integrity of the catalyst **1** was followed by NMR throughout the reaction. Over the course of the reaction, a quantity of **1** was found to convert to relatively inactive complex **8**. This is indicated by the loss of the −145.60, −149.36, −174.06, −176.81, and −185.71 ppm signals within the ^19^F NMR spectra and the growth of resonances at −140.54, −151.44, and −160.05 ppm. Workup in air, followed by precipitation with hexanes and washing with diethyl ether, resulted in the separation and isolation of toluoyl fluoride in 84% isolated yield. The more active complex **1** could be simply regenerated by treatment with Ag_2_O.

### Development of Nucleophilic Fluorination Catalyzed by **1**

The effect of altering the fluoride source was examined (Table 2). Experiments confirmed that AgF was required to achieve a high degree of fluorination. When CsF or KF was utilized as the fluorine source, a large decrease in the resultant yield was observed (Table 2, entry 2/3, respectively). Similar effects were reported by Baker and co-workers.^39^ The decrease in reactivity from AgF > CsF > KF is commonly observed and can be correlated with the larger lattice enthalpies and poorer solubility of these salts. All of these fluoride salts are sparingly soluble in solution. A high degree of fluorination using AgF (up to 90%) was retained when reducing the loading of the fluoride source to half (Table 2, entry 4), significantly reducing the quantity of waste produced, with no apparent reduction in yield.

**Table 2:** Reaction Conditions for the Fluorination of Toluoyl Chloride Catalyzed by **1**^a^

entry	fluorine source	[fluorine source]	additive	TON	^19^F NMR yield (%)^b^
1	AgF	3.0		17.6	88
2	CsF	3.0		7.2	36
3	KF	3.0		5	25
4	AgF	1.5		18.0	90
5^c^	AgF	1.5		13.6	68
6^d^	AgF	1.5		18.8	94
7^e^	AgF	1.5		12.2	61^h^
8	AgF	1.5	Ag_2_O (1.0 mmol)	2.8	14^h^
9	AgF	1.5	Ag_2_O (0.1 mmol)	18.8	94
10^f^	AgF	1.5	Ag_2_O (1.0 mmol)	6.6	33^h^
11^g^	AgF	1.5		7.2	36^h^
12	AgF	1.5	TEMPO (10 mol %)	18.0	90
13	AgF	1.5	UV 300 W lamp		6^h^

a Standard reaction conditions: Toluoyl chloride (1 mmol), fluoride source (1.5 equiv), **1** (5 mol %), DCM (5 mL), 400 rpm stirring, argon, room temperature, 24 h.

b Yields were determined by ^19^F NMR integration against an internal standard (α,α,α-trifluorotoluene, 0.163 mmol).

c 200 rpm stirring.

d 600 rpm stirring.

e 1000 rpm stirring.

f **8** used as the precatalyst.

g Reaction carried out under air.

h Catalyst destroyed under reaction conditions.

Catalyst recovery is a key consideration in homogeneous catalysis, where the recovery and reusability of the catalytic species are a fundamental requirement when analyzing the overall sustainability and commercial viability of the system, which is often overlooked.^28,45,46^ The recovery of catalyst **1** was demonstrated. The active catalyst, which was found to be partly converted to **8**, was reactivated with the addition of Ag_2_O and could be reused with a small reduction in activity. Following workup, the recovered catalyst was treated with Ag_2_O for 24 h to reform **1**, which was then reused, resulting in the recovery of up to 91% mass of the catalyst. Catalytic activity was retained upon recovery. Catalyst recovery has not been previously demonstrated for related systems.^28,39^ The strong-donor properties, late and highly chelating nature of the ligand sphere, and the later transition metal in **1** help to increase stability during recycling. Catalyst losses upon regeneration can be attributed to work at the small scale leading to mechanical losses. Methodologies including polymer-supported catalysis or organic solvent nanofiltration could be employed to optimize catalyst recycling.^47−51^

The reaction was optimized to afford a contained yield of 94% acyl fluoride (Table 2, entries 6 and 9). Stirring was required to achieve an optimum level of fluorination (Table 2, entry 6). The stirring rate must be sufficient to ensure even dispersion of AgF; however, stirring above 600 rpm led to the decomposition of the catalyst. Stirring of silver salts in an organic solvent has been shown to lead to the mechanochemical generation of silver particles and could be very important in determining the speciation of silver in solution.^43^ Silver is redox active and can influence the speciation of other solutes, including Rh complexes.

To promote recycling of the active catalytic species in situ and prevent the conversion of **1** into **8** during prolonged reaction times, the addition of different quantities of Ag_2_O was investigated. Stoichiometric addition of Ag_2_O resulted in complete decomposition of the catalyst (Table 2, entry 8), whereas the addition of 0.1 mmol Ag_2_O resulted in the observation of a high concentration of the active catalyst, **1**, in solution following the reaction (Table 2, entry 9). The addition of Ag_2_O had a negligible effect on the reaction yield (94% yield) and was discontinued, as it was found that the presence of 0.1 mmol Ag_2_O could result in decomposition of the catalyst during the reaction. This could be due to the mechanochemical conversion of Ag_2_O to silver particles and the subsequent reduction of the catalytic species. Catalyst decomposition was also observed when the reaction was conducted in air (Table 2, entry 11).

To probe whether the reaction was proceeding via a radical mechanism, the radical scavenger, TEMPO, was added; however, little effect was observed (Table 2, entry 12). Attempts to aid the reaction via photoexcitation proved unsuccessful, as on each occasion the catalyst was decomposed with very minimal formation of the fluorinated product (Table 2, entry 13). This contrasts with the ligand transfer fluorination reaction (Scheme 1D), which was promoted by UV irradiation.^40^

### Substrate Scope

**Scheme 3:**
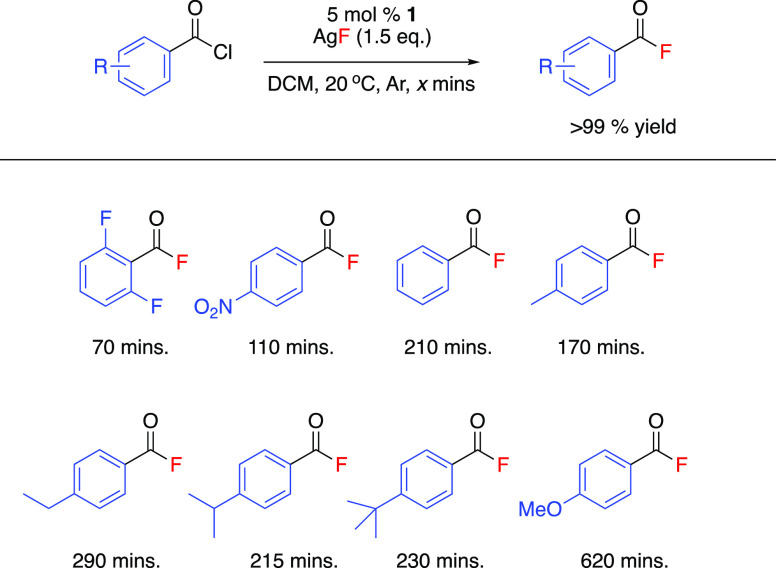
Reaction Time for the Fluorination of a Range of Acyl Chlorides Catalyzed by **1**(53)

The substrate scope of fluorination was probed by varying the substituents on the aromatic ring of the acyl chloride (Scheme 3). The fluorination of a range of acyl chlorides was carried out using 5 mol % **1**, with 1.5 equivalents of silver fluoride, in DCM, at 20 °C, with a stir rate of 600 rpm.

**Figure 2:**
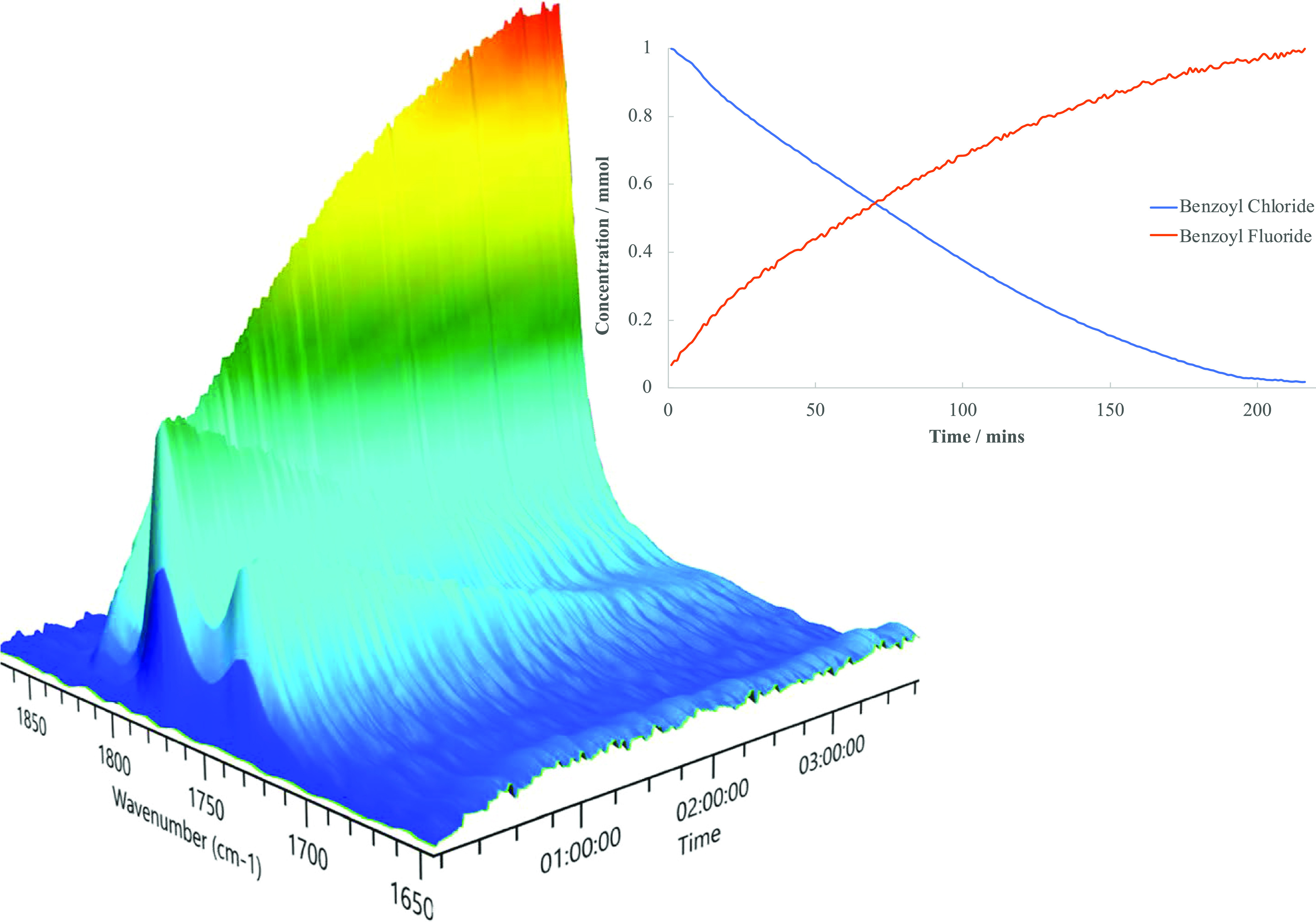
In situ ReactIR surface plot, highlighting the carbonyl region, showing the consumption of benzoyl chloride (1775 and 1736 cm^–1^) and the formation of benzoyl fluoride (1812 cm^–1^) over the course of the reaction.

Reactions were followed by FTIR using ReactIR (Figure 2). ReactIR allows for changes in concentration to be monitored in real-time, with in situ FTIR measurement results collected every minute. The characteristic carbonyl stretches of the acyl chloride and acyl fluoride functional groups were used to determine the conversion and formation of the respective species, monitoring the consumption of acyl chloride, and the reduction of its associated absorption band over time as the acyl fluoride product was formed. The surface plot shows that the benzoyl chloride reagent (1775 cm^–1^) was consumed, while the benzoyl fluoride product (1812 cm^–1^) was formed, in an A to B type transition (Figure 2). The reactant consumption and product formation traces were observed to cross at 50% relative abundance, which is indicative of a 1:1 stoichiometry between reactants and products (Scheme 3), indicating a clean reaction. This suggests that no intermediates are long-lived during the reaction, as the rate of consumption of benzoyl chloride equals the rate of formation of benzoyl fluoride. Monitoring by ReactIR enabled more precise determination of the reaction time and allowed for direct comparisons of the effects of aryl substitution on the rate of fluorination. The time taken for the reaction to complete for a range of substrates is given (Scheme 3). The use of the standard addition method (see the Supporting Information (SI): Section 1.3.3)^52^ allowed for the quantification of the starting materials and products allowing rapid and accurate concentration analysis for the reactions. Off-line quantitative ^19^F NMR analysis of the reactions confirmed the in situ measurements showing quantitative conversion of the benzoyl chloride starting material; a >99% contained ^19^F NMR yield of acyl fluoride was calculated for all substrates.

The aryl substituent played a significant role in the outcome of fluorination. The fluorination of toluoyl chloride resulted in the complete formation of the fluorinated product over the course of 170 min, with an isolated yield of 92%. Acyl chlorides bearing electron-withdrawing substituents exhibited faster reaction times, with the quantitative formation of 4-nitrobenzoyl fluoride occurring after 110 min, and an isolated yield of 94%. Off-line ^19^F NMR analysis of the reaction with 4-nitrobenzoyl chloride, 4-(α,α,α-trifluoromethyl)benzoyl chloride, and (2,3,4,5,6-pentafluoro)benzoyl chloride verified near quantitative conversion to the associated acyl fluorides (see the SI: Section 1.3.3).

As a general trend, it was observed that acyl chlorides that contained electron-withdrawing substituents in the *para*-position on the aryl ring underwent more rapid fluorination. Electron withdrawal from the carbonyl region will tend to render the acyl chloride more δ^+^ and thus more electrophilic, resulting in a greater propensity to undergo nucleophilic substitution with nucleophilic fluorine. Similarly, fast reaction times were observed for a substrate containing electron-withdrawing substituents in the *ortho*-position. 2,6-Difluorobenzoyl chloride reacted to give 2,6-difluorobenzoyl fluoride in 70 min. When less electron-withdrawing substituents were placed in the *para*-position, the length of time to achieve quantitative fluorination increased, up to 620 min in the case of 4-methoxybenzoyl chloride. The effect of changing the steric bulk of the substituents in the *para*-position was shown to have less impact on the rate of fluorination. 4-Ethylbenzoyl chloride, 4-^*i*^propylbenzoyl chloride, and 4-^*t*^butylbenzoyl chloride all achieved quantitative conversion to their analogous acyl fluorides between 215 and 290 min.

Fluorination of anhydrides leads to the quantitative formation of the acyl fluoride product over extended timeframes (see the SI: Section 1.3.3.12). Attempts to carry out the fluorination of other functional groups were conducted including benzyl halides, aldehydes, ketones, and aryl halides without success. It is concluded that these substrates are not sufficiently electrophilic. Widening the substrate scope for this fluorination reaction, including targeting alkyl chlorides, is a priority for future work.

**Scheme 4:**
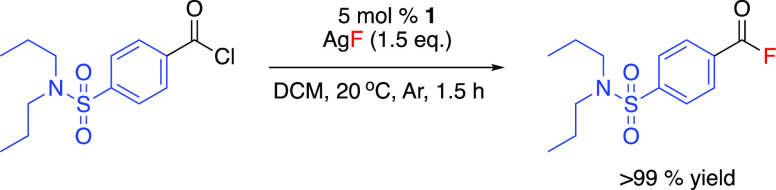
Catalytic Fluorination of the Acyl Chloride of the API Probenecid

Acyl fluoride substituents can undergo further reactions leading to functionalized biologically active pharmaceuticals.^54−56^ To investigate the utility of the protocol for pharmaceutically relevant molecules, the fluorination of the acyl chloride analogue of the API probenecid was performed. Under reaction conditions, the acyl chloride of probenecid underwent fluorination over the course of 90 min (Scheme 4), with an initial rate of 0.98 mmolh^–1^ and a TOF of 13.3 h^–1^.

**Figure 3:**
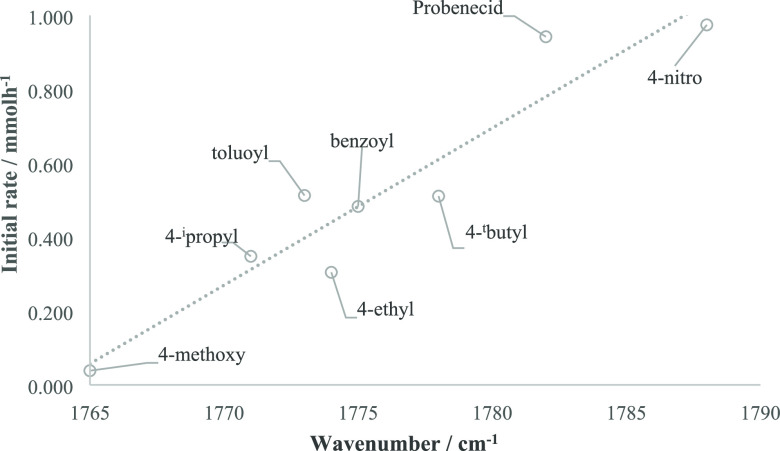
IR carbonyl stretching frequency of the acyl chloride substrates (cm^–1^) measured against the initial rate of reaction (mmolh^–1^) calculated for fluorination of the acyl chloride substrates.

These results of changing the substrate are consistent with a nucleophilic fluorination in which the attack of nucleophilic fluorine on the electrophile is the rate-determining step. The effect of the electron density of the electrophile on the reaction rate was visualized by plotting the carbonyl stretching frequency of the acyl chloride reagents with *para*-substituents against the initial rate of reaction (Figure 3). Acyl chlorides bearing electron-withdrawing groups have a higher carbonyl stretching frequency and a faster initial rate of reaction. The plot gives an approximately linear relationship.

### VTNA Graphical Kinetic Analysis and DFT Calculations

In situ IR monitoring of the reaction enabled time course concentration profiles to be analyzed. These data, in conjunction with variable time normalized analysis (VTNA),^57^ provide a tool to qualitatively assess reaction features of interest, including product inhibition and catalyst deactivation. In addition, information can be gleaned on reaction order with respect to the catalyst and other components in the system. This can be achieved by visual analysis of time-normalized reaction profiles, where the concentration plots are overlaid (see the SI: Section 1.4).

Over the time course of the catalytic fluorination of benzoyl chloride, no catalyst deactivation or product inhibition was apparent (see the SI: Figure S1a). The plots exhibited overlay between time-normalized concentration profiles. Probing the impact of catalyst activation revealed significant effects on the initial rate of the system due to catalyst preactivation (see the SI: Figure S1b). Following initial consumption of benzoyl chloride, when a second aliquot of substrate was added to the reaction vessel, fluorination was restarted with a faster initial rate than that observed for the first aliquot before following the same concentration profile. This indicates that a steady-state concentration of the active catalytic species was present in solution (see the SI: Figure S1c). This observed induction period in catalysis highlighted the need for catalyst preactivation prior to the addition of the acyl chloride substrates (see the SI: Section 1.3.3).

VTNA analysis also enabled elucidation of the initial rate order with respect to the different components within the system at turnover (see the SI: Section 1.4.2). The reaction was first order with respect to [benzoyl chloride]. The behavior was more complex with relation to [**1**], but setting the rate order to one gave the closest correlation. The initial rate constant for the reaction was calculated, from the measurements gathered from VTNA analysis, to be *k*_obs_ = 15.6 mmol^–2^ h^–1^.

**Scheme 5:**
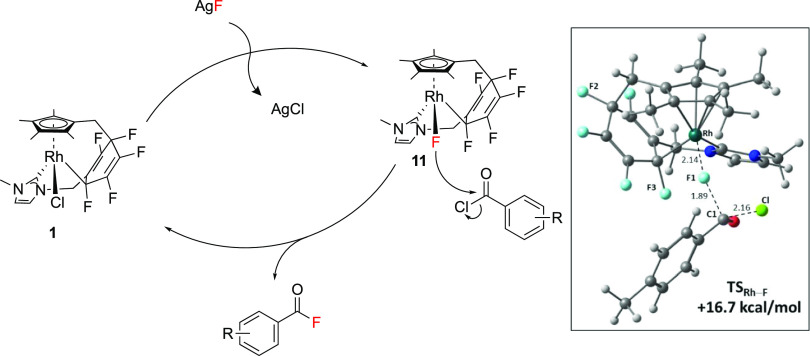
Proposed Mechanism for the Rhodium-Catalyzed Nucleophilic Fluorination of Acyl Chlorides Inset: calculated transition state for fluoride attack at para-toluoyl chloride with selected distances in Å; the computed barrier is relative to **11** + free p-toluoyl chloride.

Based on the observations described above, and supported by DFT calculations (see the SI: Section 1.5, and below), a plausible mechanism for the catalytic cycle has been proposed (Scheme 5), in agreement with the postulates of Baker and co-workers for their Co(III) catalyst (Scheme 1A).^28,39^ In the main catalytic cycle, the active catalytic species is formed through treatment of **1** with an equivalent of AgF, resulting in the formation of a metal–fluorine bond. This activated catalytic species, termed **“11”**, contains the active Rh–F bond, a fluorine of sufficient nucleophilicity to attack the electrophilic acyl chloride reagent and form the product. **11** is subsequently converted back to **1** ready to interact with AgF again.

Nucleophilic attack of the fluoride ligand in **11** was modeled with DFT calculations (see the SI: Figure S5),^58^ and a transition state was located for attack at the carbonyl carbon of toluoyl chloride (**TS_Rh-F_**, see the Scheme 5 inset). This shows elongation of both the Rh–F1 (from 2.06 Å in **11** to 2.14 Å in **TS_Rh-F_**) and C1–Cl distances in the acyl substrate (from 1.90 to 2.16 Å) with concomitant shortening of the F1···C1 distance (1.89 A in **TS_Rh-F_** cf. 1.38 Å in the toluoyl fluoride product). The associated free energy barrier (relative to **11** and free toluoyl chloride) is computed to be 16.7 kcal/mol, consistent with a very facile process once **11** is available in solution. The overall free energy change for the F/Cl exchange process to form **1** and toluoyl fluoride is −25.8 kcal/mol.^59^

Substituent effects show an enhanced rate when electron-withdrawing substituents are present on the aryl ring, indicative of nucleophilic attack of the acyl chloride electrophile being the rate-determining step. The DFT calculations provide additional qualitative support for this, with a reduced barrier of 13.1 kcal/mol computed with 4-nitrobenzoyl chloride, while that with 4-methoxybenzoyl chloride increases to 19.2 kcal/mol.

The reaction was found to be first order with respect to the substrate and approximately first order with respect to **1**. The order with respect to the substrate is straightforward, whereas the order with respect to **1** can be rationalized through a rapid proportional increase in the concentration of the active catalytic species **11** with an increased concentration of **1**, suggesting that the formation of **11** is rapid. During catalyst activation studies, an initial slow rate of reaction is present as catalyst activation occurs. In these initial stages of the reaction, the speciation of silver is not optimal, and therefore, a lower concentration of **11** results, as the formation of **11** from **1** depends on the interaction of the rhodium complex with the fluoride source AgF. This results in an induction period. This is followed by a constant rate as a steady-state concentration of the active catalytic species is present in solution. AgF is sparingly soluble, and the behavior of AgF is complex in solution; furthermore, it may not enter solution immediately in its activated form. A period of silver halide equilibration may account for the induction time observed. The successful formation of **11** is dependent on the speciation of silver. The reactions initiated in solution by the addition of AgF will lead to a high steady-state concentration of **11** under ideal conditions. However, nonideal conditions, such as excessive stirring or high AgX concentration, could lead to a loss of active catalyst through the formation of dihalide **8** analogues.

The proposed mechanism is consistent with the poor reactivity observed with less-electrophilic functional groups, as the nucleophilic attack on the electrophile is rate limiting. The reaction of anhydrides results in a very different silver halide speciation, as silver chloride concentrations will be significantly lower.

An alternative mechanism may involve nucleophilic fluoride originating from the ligand, as observed in the fluorine transfer reaction (Scheme 1D).^42^ In this mechanism, the rate-determining step would involve acyl chloride interacting with **1** to yield acyl fluoride and a partially defluorinated complex, which is regenerated to **1** or the fluorinated analogue upon interaction with silver fluoride. This mechanism is consistent with the calculated rate orders; however, computed barriers for transfer from the ligand (F2 and F3 in Scheme 5) were in excess of 40 kcal/mol (see the SI: Figure S5 inset), and we conclude that this mechanism is less likely to render a catalytic process. The DFT results suggest that no transfer fluorination from the ligand should occur under thermal conditions, and are consistent with the requirement for irradiation to promote a significant rate of stoichiometric transfer fluorination from **1**; this reaction was ideally conducted under high-intensity UV irradiation at a higher metal concentration.^42^

## Conclusions

An efficient protocol was developed for the fluorination of a range of acyl chlorides, achieving quantitative yield in as little as 1 h, under mild conditions, using a small excess of AgF as the fluoride donor. The catalyst was prepared from commercially available materials, utilizing a simple one-pot method. Workup of the catalytic fluorination reaction, through solvent/antisolvent elution and recrystallization, afforded the isolated fluorinated product in high yields and enabled recovery of the catalyst, which could be regenerated and reused. The reaction tolerated a range of substituents and was demonstrated for a substrate of pharmaceutical interest. In situ infrared measurements gave insight into the reaction mechanism. VTNA analysis of the reaction led to proposed mechanisms involving the nucleophilic fluorination of acyl chloride as the rate-determining step. DFT calculations supported a mechanism invoking a reactive Rh–F bond. Further catalyst development will concentrate on expanding the range of substrates that can be fluorinated. This may be achieved by increasing the electron density of the active catalyst, to generate a more reactive fluorine-based nucleophile.

## Supporting Information Available

Full experimental procedures/characterization, VTN analysis, and DFT calculations (PDF)

## References

[ref1] United States Food and Drug Administration Advancing Health Through Innovation: New Drug Therapy Approvals 2020; United States Food and Drug Administration: Silver Springs, MD, 2021.

[ref2] Yerien D. E.; Bonesi S.; Postigo A. Fluorination methods in drug discovery. Org. Biomol. Chem. 2016, 14, 8398–8427. 10.1039/C6OB00764C. 27506398

[ref3] Szpera R.; Moseley D. F. J.; Smith L. B.; Sterling A. J.; Gouverneur V. The Fluorination of C-H Bonds: Developments and Perspectives. Angew. Chem., Int. Ed. 2019, 58, 14824–14848. 10.1002/anie.201814457. 30759327

[ref4] Cismesia M. A.; Ryan S. J.; Bland D. C.; Sanford M. S. Multiple Approaches to the In Situ Generation of Anhydrous Tetraalkylammonium Fluoride Salts for S_N_Ar Fluorination Reactions. J. Org. Chem. 2017, 82, 5020–5026. 10.1021/acs.joc.7b00481. 28459241

[ref5] Ogiwara Y.; Hosaka S.; Sakai N. Benzoyl Fluorides as Fluorination Reagents: Reconstruction of Acyl Fluorides via Reversible Acyl C–F Bond Cleavage/Formation in Palladium Catalysis. Organometallics 2020, 39, 856–861. 10.1021/acs.organomet.0c00028.

[ref6] Ogiwara Y.; Maegawa Y.; Sakino D.; Sakai N. Palladium-catalyzed Coupling of Benzoyl Halides with Aryltrifluorosilanes Leading to Diaryl Ketones. Chem. Lett. 2016, 45, 790–792. 10.1246/cl.160223.

[ref7] Ogiwara Y.; Sakurai Y.; Hattori H.; Sakai N. Palladium-Catalyzed Reductive Conversion of Acyl Fluorides via Ligand-Controlled Decarbonylation. Org. Lett. 2018, 20, 4204–4208. 10.1021/acs.orglett.8b01582. 29963866

[ref8] Sakurai S.; Yoshida T.; Tobisu M. Iridium-catalyzed Decarbonylative Coupling of Acyl Fluorides with Arenes and Heteroarenes via C-H Activation. Chem. Lett. 2019, 48, 94–97. 10.1246/cl.180857.

[ref9] Malapit C. A.; Bour J. R.; Brigham C. E.; Sanford M. S. Base-free nickel-catalysed decarbonylative Suzuki–Miyaura coupling of acid fluorides. Nature 2018, 563, 100–104. 10.1038/s41586-018-0628-7. 30356210 PMC6212315

[ref10] Ogiwara Y.; Sakai N. Acyl Fluorides in Late-Transition-Metal Catalysis. Angew. Chem., Int. Ed. 2020, 59, 574–594. 10.1002/anie.201902805. 30969455

[ref11] Lalloo N.; Malapit C. A.; Taimoory S. M.; Brigham C. E.; Sanford M. S. Decarbonylative Fluoroalkylation at Palladium(II): From Fundamental Organometallic Studies to Catalysis. J. Am. Chem. Soc. 2021, 143, 18617–18625. 10.1021/jacs.1c08551. 34709804 PMC8693446

[ref12] Craig R.; Litvajova M.; Cronin S. A.; Connon S. J. Enantioselective acyl-transfer catalysis by fluoride ions. Chem. Commun. 2018, 54, 10108–10111. 10.1039/C8CC05692G. 30124692

[ref13] Gillard R. M.; Fernando J. E. M.; Lupton D. W. Enantioselective N-Heterocyclic Carbene Catalysis via the Dienyl Acyl Azolium. Angew. Chem., Int. Ed. 2018, 57, 4712–4716. 10.1002/anie.201712604. 29380549

[ref14] Keaveney S. T.; Schoenebeck F. Palladium-Catalyzed Decarbonylative Trifluoromethylation of Acid Fluorides. Angew. Chem., Int. Ed. 2018, 57, 4073–4077. 10.1002/anie.201800644. PMC5900963 29479784

[ref15] Olah G. A.; Nojima M.; Kerekes I. Synthetic methods and reactions. I. Seleniuum tetrafluoride and its pyridine complex. Convenient fluorinating agents for fluorination of ketones, aldehydes, amides, alcohols, carboxylic acids, and anhydrides. J. Am. Chem. Soc. 1974, 96, 925–927. 10.1021/ja00810a052.

[ref16] Olah G. A.; Nojima M.; Kerekes I. Synthetic Methods and Reactions; IV. Fluorination of Carboxylic Acids with Cyanuric Fluoride. Synthesis 1973, 8, 487–488. 10.1055/s-1973-22238.

[ref17] Paquin J.-F.; Batisse C.; Gonay M. Recent Advances in the Synthesis of Acyl Fluorides. Synthesis 2020, 53, 653–665. 10.1055/s-0040-1705951. 32691597

[ref18] Munoz S. B.; Dang H.; Ispizua-Rodriguez X.; Mathew T.; Prakash G. K. S. Direct Access to Acyl Fluorides from Carboxylic Acids Using a Phosphine/Fluoride Deoxyfluorination Reagent System. Org. Lett. 2019, 21, 1659–1663. 10.1021/acs.orglett.9b00197. 30840474

[ref19] Foth P. J.; Malig T. C.; Yu H.; Bolduc T. G.; Hein J. E.; Sammis G. M. Halide-Accelerated Acyl Fluoride Formation Using Sulfuryl Fluoride. Org. Lett. 2020, 22, 6682–6686. 10.1021/acs.orglett.0c02566. 32806146

[ref20] Song H. X.; Tian Z. Y.; Xiao J. C.; Zhang C. P. Tertiary-Amine-Initiated Synthesis of Acyl Fluorides from Carboxylic Acids and CF_3_SO_2_OCF_3_. Chem. – Eur. J. 2020, 26, 16261–16265. 10.1002/chem.202003756. 32954583

[ref21] Liang Y.; Zhao Z.; Taya A.; Shibata N. Acyl Fluorides from Carboxylic Acids, Aldehydes, or Alcohols under Oxidative Fluorination. Org. Lett. 2021, 23, 847–852. 10.1021/acs.orglett.0c04087. 33464095

[ref22] Mao S.; Kramer J. H.; Sun H. Deoxyfluorination of Carboxylic Acids with KF and Highly Electron-Deficient Fluoroarenes. J. Org. Chem. 2021, 86, 6066–6074. 10.1021/acs.joc.0c02491. 33876634

[ref23] Scattolin T.; Deckers K.; Schoenebeck F. Direct Synthesis of Acyl Fluorides from Carboxylic Acids with the Bench-Stable Solid Reagent (Me_4_N)SCF_3_. Org. Lett. 2017, 19, 5740–5743. 10.1021/acs.orglett.7b02516. 29023131

[ref24] Mulryan D.; White A. J. P.; Crimmin M. R. Organocatalyzed Fluoride Metathesis. Org. Lett. 2020, 22, 9351–9355. 10.1021/acs.orglett.0c03593. 33201721

[ref25] Brittain W. D. G.; Cobb S. L. Carboxylic Acid Deoxyfluorination and One-Pot Amide Bond Formation Using Pentafluoropyridine (PFP). Org. Lett. 2021, 23, 5793–5798. 10.1021/acs.orglett.1c01953. 34251217 PMC8397423

[ref26] Arisawa M.; Yamada T.; Yamaguchi M. Rhodium-Catalyzed Interconversion between Acid Fluorides and Thioesters Controlled using Heteroatom Acceptors. Tetrahedron Lett. 2010, 47, 6090–6092. 10.1016/j.tetlet.2010.09.009.

[ref27] Arisawa M.; Igarashi Y.; Kobayashi H.; Yamada T.; Bando K.; Ichikawa T.; Yamaguchi M. Equilibrium Shift in the Rhodium-Catalyzed Acyl Transfer Reactions. Tetrahedron 2011, 67, 7846–7859. 10.1016/j.tet.2011.07.031.

[ref28] Lee G. M.; Clément R.; Tom Baker R. High-throughput evaluation of in situ-generated cobalt(III) catalysts for acyl fluoride synthesis. Catal. Sci. Technol. 2017, 7, 4996–5003. 10.1039/C7CY01519D.

[ref29] Ajenjo J.; Destro G.; Cornelissen B.; Gouverneur V. Closing the gap between ^19^F and ^18^F chemistry. EJNMMI Radiopharm. Chem. 2021, 6, 33–70. 10.1186/s41181-021-00143-y. 34564781 PMC8464544

[ref30] Sather A. C.; Buchwald S. L. The Evolution of Pd(0)/Pd(II)-Catalyzed Aromatic Fluorination. Acc. Chem. Res. 2016, 49, 2146–2157. 10.1021/acs.accounts.6b00247. 27656765 PMC5072418

[ref31] Marchese A. D.; Adrianov T.; Lautens M. Recent Strategies for Carbon-Halogen Bond Formation Using Nickel. Angew. Chem., Int. Ed. 2021, 60, 16750–16762. 10.1002/anie.202101324. 33647169

[ref32] Grushin V. V. The Organometallic Fluorine Chemistry of Palladium and Rhodium: Studies toward Aromatic Fluorination. Acc. Chem. Res. 2010, 43, 160–171. 10.1021/ar9001763. 19788304

[ref33] Vaska L. Fluoro complexes of rhodium(I) and iridium(I). Inorg. Synth. 1974, 15, 64–68. 10.1002/9780470132463.ch14.

[ref34] Veltheer J. E.; Burger P.; Bergman R. G. Synthesis and Chemistry of the Aryliridium(III) Fluorides Cp’Ir(PMe_3_)(Aryl)F: High Reactivity due to Surprisingly Easy Ir-F Ionization. J. Am. Chem. Soc. 1995, 117, 12478–12488. 10.1021/ja00155a012.

[ref35] Bourgeois C. J.; Garratt S. A.; Hughes R. P.; Larichev R. B.; Smith J. M.; Ward A. J.; Willemsen S.; Zhang D.; DiPasquale A. G.; Zakharov L. N.; Rheingold A. L. Synthesis and Structural Characterization of (Perfluoroalkyl)fluoroiridium(III) and (Perfluoroalkyl)methyliridium(III) Compounds. Organometallics 2006, 25, 3474–3480. 10.1021/om060267s.

[ref36] Maity A.; Stanek R. J.; Anderson B. L.; Zeller M.; Hunter A. D.; Moore C. E.; Rheingold A. L.; Gray T. G. Fluoride complexes of cyclometallated iridium(III). Organometallics 2015, 34, 109–120. 10.1021/om5009555.

[ref37] Chong E.; Kampf J. W.; Ariafard A.; Canty A. J.; Sanford M. S. Oxidatively Induced C-H Activation at High Valent Nickel. J. Am. Chem. Soc. 2017, 139, 6058–6061. 10.1021/jacs.7b02387. 28425702

[ref38] Malapit C. A.; Bour J. R.; Laursen S. R.; Sanford M. S. Mechanism and Scope of Nickel-Catalyzed Decarbonylative Borylation of Carboxylic Acid Fluorides. J. Am. Chem. Soc. 2019, 141, 17322–17330. 10.1021/jacs.9b08961. 31617708 PMC11103277

[ref39] Leclerc M. C.; Bayne J. M.; Lee G. M.; Gorelsky S. I.; Vasiliu M.; Korobkov I.; Harrison D. J.; Dixon D. A.; Baker R. T. Perfluoroalkyl Cobalt(III) Fluoride and Bis(perfluoroalkyl) Complexes: Catalytic Fluorination and Selective Difluorocarbene Formation. J. Am. Chem. Soc. 2015, 137, 16064–16073. 10.1021/jacs.5b12003. 26674217

[ref40] Pupo G.; Vicini A. C.; Ascough D. M. H.; Ibba F.; Christensen K. E.; Thompson A. L.; Brown J. M.; Paton R. S.; Gouverneur V. Hydrogen Bonding Phase-Transfer Catalysis with Potassium Fluoride: Enantioselective Synthesis of β-Fluoroamines. J. Am. Chem. Soc. 2019, 141, 2878–2883. 10.1021/jacs.8b12568. 30689372

[ref41] See Y. Y.; Morales-Colón M. T.; Bland D. C.; Sanford M. S. Development of S_N_Ar Nucleophilic Fluorination: A Fruitful Academia-Industry Collaboration. Acc. Chem. Res. 2020, 53, 2372–2383. 10.1021/acs.accounts.0c00471. 32969213

[ref42] Morgan P. J.; Hanson-Heine M. W. D.; Thomas H. P.; Saunders G. C.; Marr A. C.; Licence P. C–F Bond Activation of a Perfluorinated Ligand Leading to Nucleophilic Fluorination of an Organic Electrophile. Organometallics 2020, 39, 2116–2124. 10.1021/acs.organomet.0c00176.

[ref43] Thomas H. P.; Wang Y.-M.; Lorenzini F.; Coles S. J.; Horton P. N.; Marr A. C.; Saunders G. C. Cyclometalation via Carbon–Fluorine Bond Activation Induced by Silver Particles. Organometallics 2017, 36, 960–963. 10.1021/acs.organomet.6b00872.

[ref44] Thomas H. P.; Marr A. C.; Morgan P. J.; Saunders G. C. Tethering of Pentamethylcyclopentadienyl and N-Heterocycle Stabilized Carbene Ligands by Intramolecular 1,4-Addition to a Polyfluorophenyl Substituent. Organometallics 2018, 37, 1339–1341. 10.1021/acs.organomet.8b00164.

[ref45] Sorlin A. M.; Mixdorf J. C.; Rotella M. E.; Martin R. T.; Gutierrez O.; Nguyen H. M. The Role of Trichloroacetimidate To Enable Iridium-Catalyzed Regio- and Enantioselective Allylic Fluorination: A Combined Experimental and Computational Study. J. Am. Chem. Soc. 2019, 141, 14843–14852. 10.1021/jacs.9b07575. 31438667

[ref46] Zhang Q.; Mixdorf J. C.; Reynders G. J.; Nguyen H. M. Rhodium-catalyzed benzylic fluorination of trichloroacetimidates. Tetrahedron 2015, 71, 5932–5938. 10.1016/j.tet.2015.04.066.

[ref47] Shende V. S.; Saptal V. B.; Bhanage B. M. Recent Advances Utilized in the Recycling of Homogeneous Catalysis. Chem. Rec. 2019, 19, 2022–2043. 10.1002/tcr.201800205. 31021522

[ref48] Dreimanna J.; Lutze P.; Zagajewski M.; Behr A.; Górak A.; Vorholt A. J. Highly integrated reactor–separator systems for the recycling of homogeneous catalysts. Chem. Eng. Process. 2016, 99, 124–131. 10.1016/j.cep.2015.07.019.

[ref49] Dreimann J. M.; Skiborowski M.; Behr A.; Vorholt A. J. Recycling Homogeneous Catalysts Simply by Organic Solvent Nanofiltration: New Ways to Efficient Catalysis. ChemCatChem 2016, 8, 3330–3333. 10.1002/cctc.201601018.

[ref50] Cole-Hamilton D. J. Homogeneous catalysis--new approaches to catalyst separation, recovery, and recycling. Science 2003, 299, 1702–1706. 10.1126/science.1081881. 12637737

[ref51] Bianga J.; Künnemann K. U.; Gaide T.; Vorholt A. J.; Seidensticker T.; Dreimann J. M.; Vogt D. Thermomorphic Multiphase Systems: Switchable Solvent Mixtures for the Recovery of Homogeneous Catalysts in Batch and Flow Processes. Chem. – Eur. J. 2019, 25, 11586–11608. 10.1002/chem.201902154. 31241213

[ref52] Hutchinson G.; Welsh C. D. M.; Burés J. Use of Standard Addition to Quantify In Situ FTIR Reaction Data. J. Org. Chem. 2021, 86, 2012–2016. 10.1021/acs.joc.0c02684. 33356249

[ref53] Reaction times correspond to the time taken for each substrate to reach quantitative conversion from the acyl chloride substrate, as monitored by in situ FTIR, calibrated against known concentrations of the acyl chloride substrate. Contained yield of the acyl fluoride product calculated by off-line ^19^F NMR analysis vs an internal standard.

[ref54] Ogiwara Y.; Iino Y.; Sakai N. Catalytic C–H/C–F Coupling of Azoles and Acyl Fluorides. Chem. – Eur. J. 2019, 25, 6513–6516. 10.1002/chem.201901219. 30941769

[ref55] Pan F.-F.; Guo P.; Li C.-L.; Su P.; Shu X.-Z. Enones from Acid Fluorides and Vinyl Triflates by Reductive Nickel Catalysis. Org. Lett. 2019, 21, 3701–3705. 10.1021/acs.orglett.9b01164. 31066568

[ref56] Kayumov M.; Zhao J. N.; Mirzaakhmedov S.; Wang D. Y.; Zhang A. Synthesis of Arylstannanes via Palladium-Catalyzed Decarbonylative Coupling of Aroyl Fluorides. Adv. Synth. Catal. 2020, 362, 776–781. 10.1002/adsc.201901223.

[ref57] Nielsen C. D.; Burés J. Visual kinetic analysis. Chem. Sci. 2019, 10, 348–353. 10.1039/C8SC04698K. 30746083 PMC6335952

[ref58] DFT calculations employed the BP86 functional with SDD pseudopotentials and basis sets on Rh and Cl (with added d-orbital polarization on the latter) and 6-31g** basis sets for other atoms. Optimizations were performed including the effects of the dichloromethane solvent (PCM method). Electronic energies were recomputed with the ωB97x-D functional with def2tzvp basis sets. These energies were combined with the thermochemical corrections from the BP86 frequency calculations to give the free energies quoted in the text. See Supporting Materials for full details and references.

[ref59] IRC calculations on TS_Rh-F_ showed that the toluoyl fluoride product initially binds to Rh through fluorine and has a computed free energy of +3.1 kcal/mol). The O-bound isomer is located at -4.3 kcal/mol. The structures are all computed as contact ion-pairs with Cl- in the outer sphere. We assume that the final substitution of toluoyl fluoride by chloride to reform **1** is a facile process.

