# Supplementary material for: Nucleophilic Fluorination Catalyzed by a Cyclometallated Rhodium Complex
Source: Organometallics. 2022 Mar 31;41(7):883–91. doi: 10.1021/acs.organomet.2c00052 (PMC9098193; doi:10.1021/acs.organomet.2c00052)
Supplement: Supplementary file 1 — om2c00052_si_001.pdf [file om2c00052_si_001.pdf]

Supporting Information

Nucleophilic Fluorination Catalyzed by a Cyclometallated Rhodium Complex

Patrick J. Morgan,[a] Graham C. Saunders,[b] Stuart A. Macgregor,[c]* Andrew C. Marr,[d]* Peter Licence.[a]*

[a]GSK Carbon Neutral Laboratory, School of Chemistry, University of Nottingham, Nottingham NG7 2TU, United Kingdom.

[b]School of Science, University of Waikato, Hamilton 3240, New Zealand.

[c]School of Engineering and Physical Sciences, William H. Perkin Building, Heriot-Watt University, Edinburgh EH14 4AS, United Kingdom.

[d]School of Chemistry and Chemical Engineering, Queen's University Belfast, David Keir Building, Belfast BT9 5AG, United Kingdom.

*E-mail: s.a.macgregor@hw.ac.uk (S.A.M.), a.marr@qub.ac.uk (A.C.M.), peter.licence@nottingham.ac.uk (P.L.).

Table of Contents

1 Experimental details ... 3

1.1 Instrumentation ... 3

1.2 Materials ... 3

1.3 Catalytic Fluorination ... 4

1.3.1 General procedure for the catalyst screen ... 4

1.3.2 General procedure for further development ... 4

1.3.3 General procedure for substrate scope ... 4

1.3.3.1 Fluorination of benzoyl chloride ... 6

1.3.3.2 Fluorination of toluoyl chloride ... 7

1.3.3.3 Fluorination of 4-nitrobenzoyl chloride ... 8

1.3.3.4 Fluorination of 2,6-difluorobenzoyl chloride ... 9

1.3.3.5 Fluorination of (2,3,4,5,6)-pentafluorobenzoyl chloride ... 10

1.3.3.6 Fluorination of 4-(trifluoromethyl)benzoyl chloride ... 10

1.3.3.7 Fluorination of 4-ethylbenzoyl chloride ... 11

1.3.3.8 Fluorination of 4-^{i}propylbenzoyl chloride ... 12

1.3.3.9 Fluorination of 4-^{t}butylbenzoyl chloride ... 13

1.3.3.10 Fluorination of 4-methoxybenzoyl chloride ... 14

1.3.3.11 Fluorination of probenecid chloride ... 15

1.4 Visual Time Normalised Analysis ... 17

1.4.1 VTN analysis ... 17

1.4.2 Rate order calculation from VTNA analysis ... 19

1.4.3 Initial rate calculations 21
1.4.4 Calibration curve 22

1.5 Computations **23**

1.6 References **25**

2 Characterisation ***27***

Figure S6.^{19}F NMR spectrum of the recovered catalyst, pre-regeneration with Ag_2O. **27**

Figure S7. ^{19}F NMR spectrum of the recovered catalyst, post-regeneration with Ag_2O. **28**

Figure S8. NMR spectra of benzoyl fluoride **29**

Figure S9. NMR spectra of toluoyl fluoride. **31**

Figure S10. NMR spectra of 4-nitrobenzoyl fluoride. **32**

Figure S11. NMR spectra of 2,6-difluorobenzoyl fluoride. **34**

Figure S12. NMR spectra of 2,3,4,5,6-(pentafluoro)benzoyl fluoride. **36**

Figure S13. NMR spectra of 4-(trifluoromethyl)benzoyl fluoride. **37**

Figure S14. NMR spectra of 4-ethylbenzoyl fluoride. **38**

Figure S15. NMR spectra of 4-^{i}propylbenzoyl fluoride. **39**

Figure S16. NMR spectra of 4-^{t}butylbenzoyl fluoride. **40**

Figure S17. NMR spectra of 4-methoxybenzoyl fluoride. **41**

Figure S18. NMR spectra of probenecid fluoride. **43**

1 Experimental details

1.1 Instrumentation

NMR spectral analysis was carried out using a Bruker Ascend 400 spectrometer (400 MHz) and Bruker Ascend 500 spectrometer (500 MHz) at room temperature (≈ 300 K). ^{1}H and ^{13}C NMR spectra were calibrated to the corresponding solvent signals ($CDCl_3$: 7.26 ppm for ^{1}H, 77.16 ppm for ^{13}C;). The chemical shifts are reported in ppm and coupling constants are given in Hz. A 60s delay was used for quantitative ^{19}F NMR integration. NMR data was processed using MestReNova software. Multiplicity assignments in NMR spectra are labelled as follows: "*s*" = singlet, "*d*"= doublet, "*t*"= triplet, "*q*" = quartet, "*p*" = pentet, "*m*" = multiplet. Electrospray mass spectra were recorded on a Brucker micrOTOF II with Agilent technologies 1200 Infinity Series mass spectrometer. *In-situ* ReactIR measurements conducted using Mettler Toledo ReactIR 15, collecting a spectral average of 256 scans at a scan rate of 256 scans per minute. Experiment set up and analysed using Mettler Toledo iC IR software, using a Mettler Toledo Easy Max basic synthetic workstation. Glassware was oven-dried, evacuated and backfilled with argon before use.

1.2 Materials

RhCp*(Cl_2)(F_5Bzmim), IrCp*(Cl_2)(F_5Bzmim), [Cp*IrCl(κC^2-$MeNC_3H_2NCH_2C_6F_4$)], **9**, [Cp*RhCl(κC^2-$MeNC_3H_2NCH_2C_6F_4$)], **10** and [(η^5,κ^2C-$C_5Me_4CH_2C_6F_5CH_2NC_3H_2NMe$)-RhCl], **1** were synthesized as previously described.[1–3] 3-methyl-1-(3,4,5,6-tetrafluorobenzyl)-imidazolium bromide was synthesized using a method similar to a published procedure.[1] [IrCl(CO)(PPh_3)$_2$] and IrCl(CO)(PPh_3)$_2$ were synthesized following literature procedures.[4] [IrCp*Cl_2]$_2$ and [IrCp*I_2]$_2$, 1-methylimidazole, silver oxide, toluoyl chloride, benzoyl chloride, 2,6-difluorobenzoyl chloride, 4-methoxybenzoyl chloride, 4-^{t}butylbenzoyl chloride and probenecid were purchased from Sigma Aldrich (Merck). [RhCp*Cl_2]$_2$, 4-nitrobenzoyl chloride, 4-ethylbenzoyl chloride, 4-^{i}propylbenzoyl chloride, 4-^{t}butylbenzoyl chloride, 4-(trifluoromethyl)benzoyl chloride, 2,3,4,5,6-pentafluorobenzoyl chloride, benzyl bromide and 2-chloroacetaphenone were purchased from Alfa Aesar. Acetic anhydride was purchased from VWR. 2,3,4,5,6-pentafluorobenzyl bromide and 3,4,5,6-tetrafluorobenzyl bromide were purchased from Fluorochem. 3-chloropenta-2,4-dione was purchased from Acros Organics. All solvents were obtained from a solvent purification system, with the exception of dichloromethane, which was supplied anhydrous in a sure seal bottle, and used as required without further purification.

1.3 Catalytic Fluorination

1.3.1 General procedure for the catalyst screen

Catalyst (0.05 mmol, 5 mol %) and silver fluoride (380 mg, 3.0 eq) were added to a 50 mL round bottomed flask, with a side arm, along with a stirring bar. The flask was evacuated and backfilled with argon three times, with care taken when exposing the evacuated flask to the inert atmosphere to avoid disturbance of the solid. Anhydrous DCM (5 mL) was added via syringe and the reaction mixture was allowed to stir under argon, in darkness for 10 minutes. Toluoyl chloride (1.0 mmol) was added via pipette under a dynamic flow of argon and the reaction was sealed and stirred in darkness for 24 hours. After the reaction time had elapsed the reaction mixture was filtered through a plug of celite, washed with DCM (3 x 5 mL) and the solvent was removed under vacuum. The resulting orange oily residue was transferred to an NMR tube, with the aid of deuterated chloroform (0.5 mL) alongside *α,α,α*-trifluorotoluene (20 µL). Internal contained yield of the toluoyl fluoride product was determined against the *α,α,α*-trifluorotoluene internal standard.

1.3.2 General procedure for further development

Unless otherwise stated, [($C_5Me_4CH_2C_6F_5CH_2NC_3H_2NMe$)- RhCl]**, 1** (27 mg, 0.05 mmol, 5 mol %) and silver fluoride (190 mg, 1.5 eq) were added, alongside any additive, to a 50 mL round bottomed flask, with a side arm, along with a stirring bar. The flask was evacuated and backfilled with argon 3 times, with care taken when exposing the evacuated flask to the inert atmosphere to avoid disturbance of the solid. Anhydrous DCM (5 mL) was added via syringe and the reaction mixture was allowed to stir (400 rpm) under argon, in darkness for 10 minutes. Toluoyl chloride (1.0 mmol) was added via pipette under a dynamic flow of argon and the reaction was sealed and stirred in darkness for 24 hours. After the reaction time had elapsed the reaction mixture was filtered through a plug of celite, washed with DCM (3 x 5 mL) and the solvent was removed under vacuum. The resulting orange oily residue was transferred to an NMR tube, with the aid of deuterated chloroform (0.5 mL) alongside *α,α,α*-trifluorotoluene (20 µL). Internal contained yield of the toluoyl fluoride product was determined against the *α,α,α*-trifluorotoluene internal standard.

1.3.3 General procedure for substrate scope

[($C_5Me_4CH_2C_6F_5CH_2NC_3H_2NMe$)- RhCl]**, 1** (27 mg, 0.05 mmol, 5 mol %) and silver fluoride (190 mg, 1.5 eq) were added to a Schlenk finger, alongside a stir bar and the flask was evacuated and backfilled. The sealed Schlenk finger was placed in a Mettler Toledo Easy Max basic synthetic workstation and heated to 20 °C. The IR probe was inserted under a positive pressure of argon and a background taken using Mettler Toledo iC IR software. Anhydrous DCM (5 mL) was added via syringe and the experiment

was started, with a stir rate of 600 rpm, for 10 minutes prior to the addition of the acyl chloride substrate. The acyl chloride (1.0 mmol) was added, using the standard addition method,[5] following this period of catalyst activation. The use of this standard addition allows for the concentration of reaction components to be quantified, without the need for offline sampling and analysis. This standard addition method involved the addition of the acyl chloride substrate to the reaction vessel in two additions, via pipette, allowing for a complete scan to occur between additions. The solids, 4-nitrobenzoyl chloride and probenecid chloride, were added carefully under a positive pressure of argon in two additions following the standard addition method. Changes that occurred were monitored over time *via* the "solvent abstraction" feature of the iC IR software, and trendlines showing the consumption of acyl chloride and the formation of acyl fluoride were plotted. The reaction was stopped once the formation of the acyl fluoride product plateaued.

In the case of benzoyl chloride, the benzoyl fluoride product is commercially available. Following completion of the reaction, benzoyl fluoride (2 x 0.5 mmol) was doped into the reaction following the double addition method. This allows for quantification of both the acyl chloride reagent and acyl fluoride product. Normalisation of the absorption intensities of the reagent and product to 1 mmol allowed for quantitative measurements to be assigned.

After the absorption peak of the acyl fluoride product plateaued the reaction mixture was filtered through a plug of celite, washed with DCM (3 x 5 mL) and the solvent was removed under vacuum. Off-line ^{19}F NMR analysis of reaction mixture, integrated against the internal standard *α,α,α*-trifluorotoluene (20 µL), enabled secondary quantitative measurement to be completed. Following NMR measurements, the NMR solution was eluted with diethyl ether (5 mL) and placed at *ca*. -20 °C for a minimum of 16 hours. Filtration of the resultant mixture enable recovery of the catalyst and a pale orange solution. Solvent was then removed under vacuum and washed with a minimal quantity (2-5 mL) of hot heptane, the solution was decanted and then cooled *ca*. -20 °C for a minimum of 16 hours to aid recrystallisation of the solid acyl fluoride products. The solvent was then removed under vacuum, to give a colourless oil or solid, with was dried under vacuum and isolated to give 79 - 92 % isolated yield of the acyl fluoride products. This double elution methodology also enabled the recovery of up to 91 % of the mass of the catalyst, which could be regenerated with Ag_2O (1.5 eq.) and reused.

1.3.3.1 Fluorination of benzoyl chloride

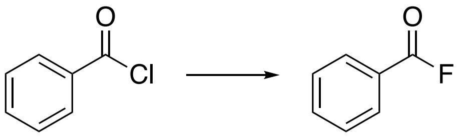

Benzoyl fluoride was formed using the general method (*Section* 1.3.3). Benzoyl chloride (1.0 mmol) was added to a Schlenk tube containing **1** (27 mg, 0.05 mmol) and AgF (190 mg, 1.5 eq.) in dry DCM (5 mL), which had previously been stirred for 10 minutes. The reaction was stirred at 20 °C for 210 minutes, until all of the starting material had been consumed, followed by work-up via filtration through celite, removal of solvent and transfer to a NMR tube, alongside deuterated chloroform (0.5 mL) and α,α,α-trifluorotoluene (20 µL). ^{19}F NMR yield *vs*. the internal standard > 99 %, isolated yield of colourless oil, 108 mg, 87 % yield.

^{1}H NMR (400 Mhz, d_1-chloroform): δ 8.03 (m, C_6-*H,* 2H), 7.70 (tt, J_{HH} = 7.6, 1.2 Hz, C_6-*H*, 1H), 7.52 (m, C_6-*H*, 2H).**^{19}F NMR** (376 MHz, d_1-chloroform): δ 18.05 (s, CO*F*, 1F). **^{13}C NMR** (101 MHz, d_1-chloroform): δ 157.4 (d, J_{CF} = 344.2 Hz, *C*(O)F), 135.4 (s, 4-*C*), 131.4 (d, J_{CF} = 4.0 Hz, 2,6-*C*), 129.1 (s, 3,5-*C*), 124.9 (d, J_{CF} = 60.9 Hz, *C*C(O)F). **HRMS** (ESI): Theoretical $[M]^+$ $[C_7H_5O]^+$ 105.0334; found for $[C_7H_5O]^+$ 105.0333. **IR**(*COF*): 1812 cm^{-1}.[7]

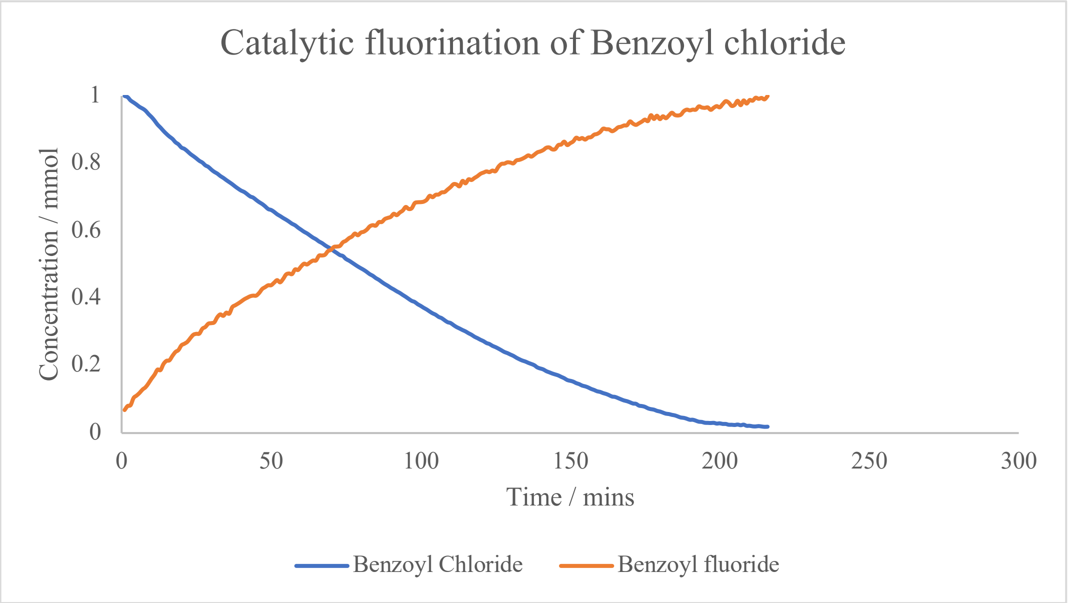

1.3.3.2 Fluorination of toluoyl chloride

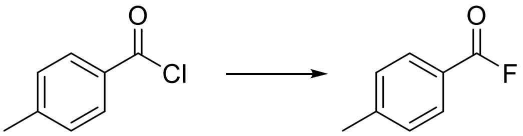

Toluoyl fluoride was formed using the general method (*Section* 1.3.3). Toluoyl chloride (1.0 mmol) was added to a Schlenk tube containing **1** (27 mg, 0.05 mmol) and AgF (190 mg, 1.5 eq.) in dry DCM (5 mL), which had previously been stirred for 10 minutes. The reaction was stirred at 20 °C for 170 minutes, until all of the starting material had been consumed, followed by work-up via filtration through celite, removal of solvent and transfer to a NMR tube, alongside deuterated chloroform (0.5 mL) and α,α,α-trifluorotoluene (20 µL). ^{19}F NMR yield *vs*. the internal standard > 99 %, isolated yield of colourless solid, 126 mg, 92 % yield.

^{1}H NMR (400 Mhz, d_1-chloroform): δ 8.01 (d, J_{HH} = 8.3 Hz, C_6-*H*, 2H), δ 7.28 (d, J_{HH} = 8.1 Hz, C_6-*H*, 2H), 2.43 (s, Me, 3H). **^{19}F NMR** (376 MHz, d_1-chloroform): δ 17.46 (s, CO*F*, 1F).[8] **HRMS** (ESI): Theoretical $[M]^+$ $[C_8H_7O]^+$ 119.0491; found for $[C_8H_7O]^+$ 119.0490. **IR**(*CO*F): 1805 cm^{-1}.[8]

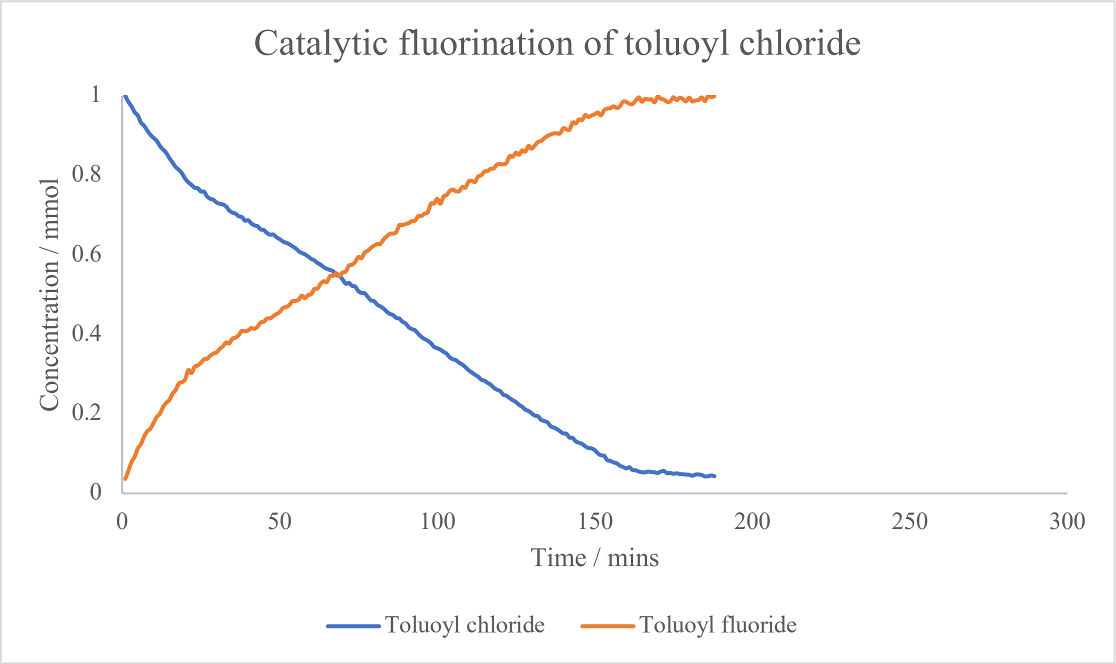

1.3.3.3 Fluorination of 4-nitrobenzoyl chloride

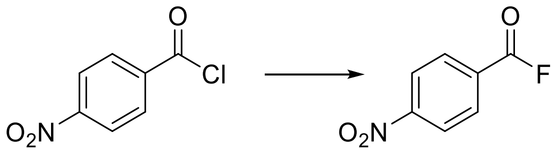

4-nitrobenzoyl fluoride was formed using the general method (*Section* 1.3.3). 4-nitrobenzoyl chloride (1.0 mmol) was added to a Schlenk tube containing **1** (27 mg, 0.05 mmol) and AgF (190 mg, 1.5 eq.) in dry DCM (5 mL), which had previously been stirred for 10 minutes. The reaction was stirred at 20 °C for 110 minutes, until all of the starting material had been consumed, followed by work-up via filtration through celite, removal of solvent and transfer to a NMR tube, alongside deuterated chloroform (0.5 mL) and α,α,α-trifluorotoluene (20 µL). ^{19}F NMR yield *vs*. the internal standard > 99 %, isolated yield of pale-yellow solid, 156 mg, 92 % yield.

^{1}H NMR (400 Mhz, d_1-chloroform): δ 8.38 (d, J_{HH} = 7.8 Hz, C_6-*H*, 2H), δ 8.25 (d, J_{HH} = 8.8 Hz, C_6-*H*, 2H). **^{19}F NMR** (376 MHz, d_1-chloroform): δ 20.36 (s, CO*F*, 1F).[9] **^{13}C NMR** (101 MHz, d_1-chloroform): δ155.6 (d, J_{CF} = 346.4 Hz, *C*(O)F), 152.0 (s, O_2N-*C)*, 132.7 (d, J_{CF} = 3.5 Hz, 2,6-*C*), 130.4 (d, J_{CF} = 63.2 Hz, *C*C(O)F), 124.3 (s, 3,5-*C*). **HRMS** (ESI): Theoretical $[M]^+$ $[C_7H_4NO_3]^+$ 150.0187; found for $[C_7H_4NO_3]^+$ 150.0178. **IR**(*C*O*F*): 1821 cm^{-1}.[9]

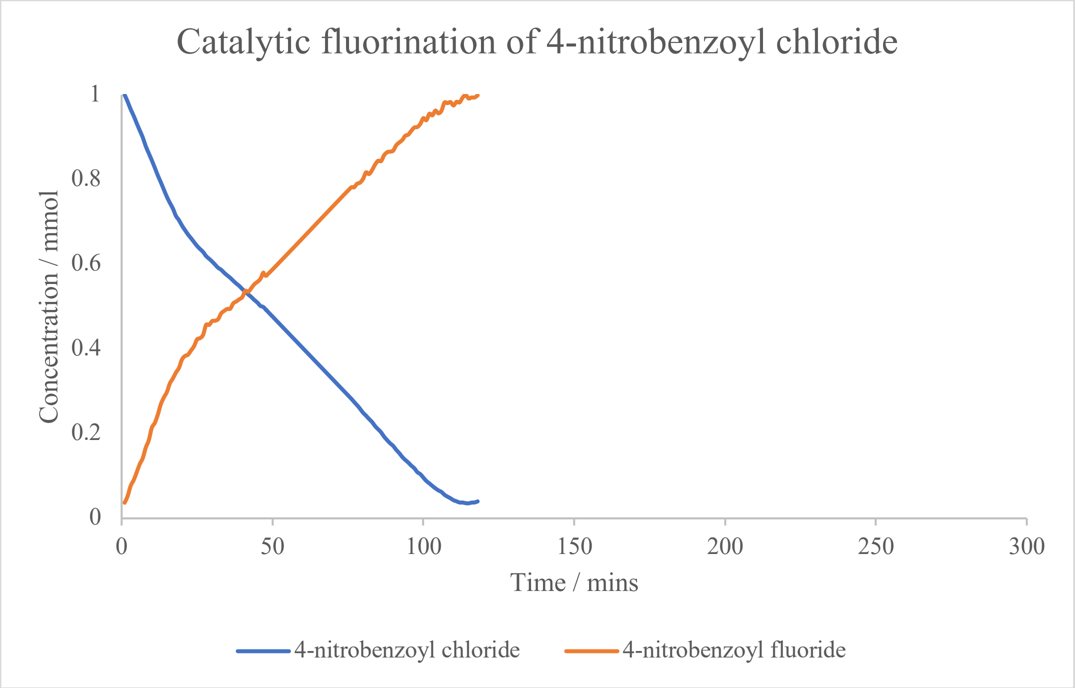

1.3.3.4 *Fluorination of 2,6-difluorobenzoyl chloride*

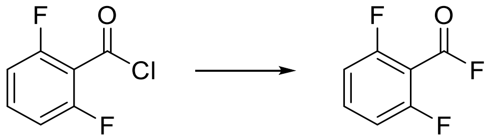

2,6-difluorobenzoyl fluoride was formed using the general method (*Section* 1.3.3). 2,6-difluorobenzoyl chloride (1.0 mmol) was added to a Schlenk tube containing **1** (27 mg, 0.05 mmol) and AgF (190 mg, 1.5 eq.) in dry DCM (5 mL), which had previously been stirred for 10 minutes. The reaction was stirred at 20 °C for 70 minutes, until all of the starting material had been consumed, followed by work-up via filtration through celite, removal of solvent and transfer to a NMR tube, alongside deuterated chloroform (0.5 mL) and α,α,α-trifluorotoluene (20 μL). ^{19}F NMR yield *vs*. the internal standard > 99 %, isolated yield of colourless solid, 158 mg, 83 % yield.

^{1}H NMR (400 Mhz, d_1-chloroform): δ 7.48 (tt, J_{HH} = 8.5, 6.1 Hz, C_6-*H*, 2H), 7.00 (t, J_{HH} = 8.4 Hz, C_6-*H*, 1H). **^{19}F NMR** (376 MHz, d_1-chloroform): δ 47.50 (t, J_{FF} = 38.7 Hz, CO*F*, 1F), -108.39 (s, C*F*, 2F). **HRMS** (ESI): Theoretical $[M]^+$ $[C_7H_3OF_2]^+$ 141.0146; found for $[C_7H_3OF_2]^+$ 141.0149. **IR**(CO*F*): 1822 cm^{-1}.

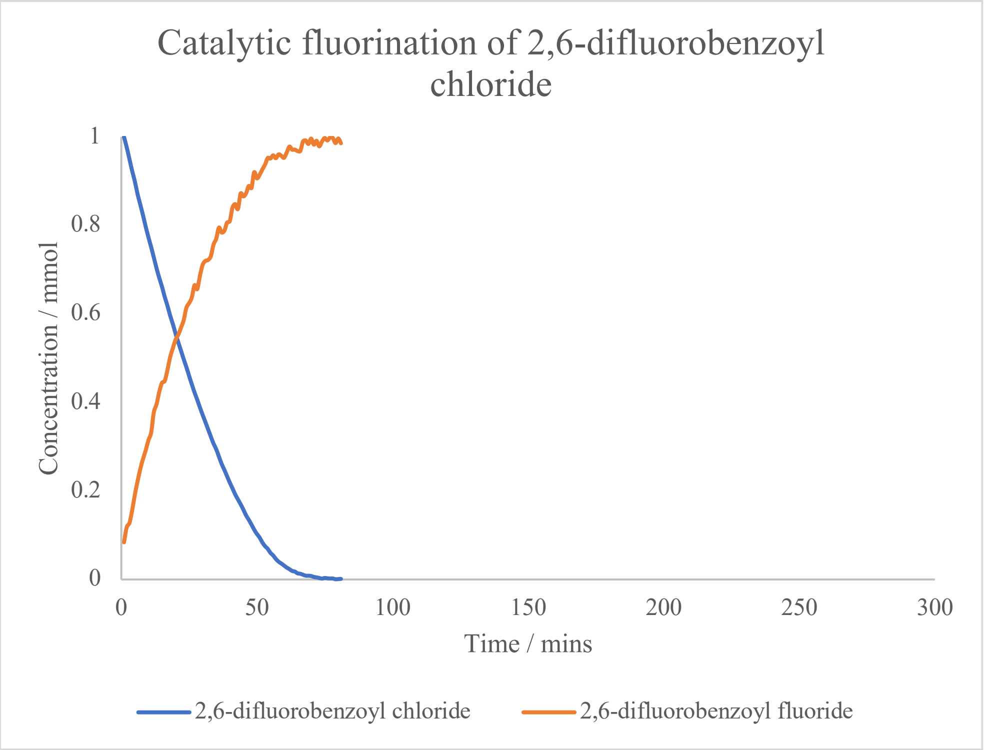

1.3.3.5 *Fluorination of (2,3,4,5,6)-pentafluorobenzoyl chloride*

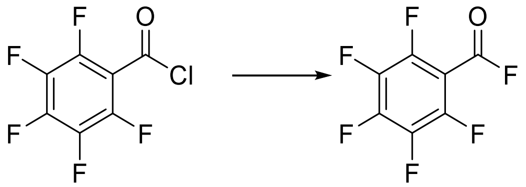

2,3,4,5,6-pentafluorobenzoyl chloride (1.0 mmol) was added to a Schlenk tube containing **1** (27 mg, 0.05 mmol) and AgF (190 mg, 1.5 eq.) in dry DCM (5 mL), which had previously been stirred for 10 minutes. The reaction was stirred at 20 °C for four hours, followed by work-up via filtration through celite, removal of solvent and transfer to a NMR tube, alongside deuterated chloroform (0.5 mL) and α,α,α-trifluorotoluene (20 µL). ^{19}F NMR yield *vs*. the internal standard > 99 %, isolated yield of pale orange solid, 168 mg, 78 % yield.

^{19}F NMR (376 MHz, d_1-chloroform): δ 47.60 (t, J_{FF}= 40.6 Hz, CO*F*, 1F), -136.49 (m, C_6-*F*, 2F), -146.56 (m, C_6-*F*, 1F), -159.96 (m, C_6-*F*, 2F). **HRMS** (ESI): Theoretical $[M]^+$ $[C_7OF_5]^+$ 194.9874; found for $[C_7OF_5]^+$ 194.9879. **IR**(*CO*F): 1830 cm^{-1}.[10]

1.3.3.6 *Fluorination of 4-(trifluoromethyl)benzoyl chloride*

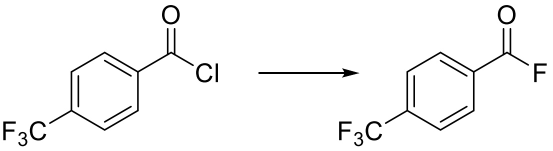

4-(trifluoromethyl)benzoyl chloride (1.0 mmol) was added to a Schlenk tube containing **1** (27 mg, 0.05 mmol) and AgF (190 mg, 1.5 eq.) in dry DCM (5 mL), which had previously been stirred for 10 minutes. The reaction was stirred at 20 °C for four hours, followed by work-up via filtration through celite, removal of solvent and transfer to a NMR tube, alongside deuterated chloroform (0.5 mL) and α,α,α-trifluorotoluene (20 µL). ^{19}F NMR yield *vs*. the internal standard > 99 %.

^{1}H NMR (400 Mhz, d_1-chloroform): δ 8.39 (d, J_{HH} = 8.2 Hz, C_6-*H*, 2H), δ 8.25 (d, J_{HH} = 8.8 Hz, C_6-*H*, 2H). **^{19}F NMR** (376 MHz, d_1-chloroform): δ 20.36 (s, CO*F*, 1F) -62.78 (s, C*F₃*, 3F). **MS** (ESI): Theoretical $[M]^+$ $[C_8H_4OF_3]^+$ 173.02; found for $[C_8H_4OF_3]^+$ 173.01. **IR**(*CO*F): 1817 cm^{-1}.[11]

1.3.3.7 *Fluorination of 4-ethylbenzoyl chloride*

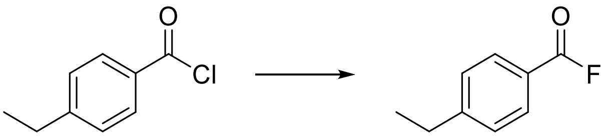

4-ethylbenzoyl fluoride was formed using the general method (*Section* 1.3.3). 4-ethylbenzoyl chloride (1.0 mmol) was added to a Schlenk tube containing **1** (27 mg, 0.05 mmol) and AgF (190 mg, 1.5 eq.) in dry DCM (5 mL), which had previously been stirred for 10 minutes. The reaction was stirred at 20 °C for 290 minutes, until all of the starting material had been consumed, followed by work-up via filtration through celite, removal of solvent and transfer to a NMR tube, alongside deuterated chloroform (0.5 mL) and α,α,α-trifluorotoluene (20 µL). ^{19}F NMR yield *vs*. the internal standard > 99 %, isolated yield of colourless solid, 158 mg, 83 % yield.

^{1}H NMR (400 Mhz, d_1-chloroform): δ 7.95 (d, J_{HH} = 8.3 Hz, C_6-*H*, 2H), δ 7.35 (d, J_{HH} = 7.9 Hz, C_6-*H*, 2H), 2.75 (q, J_{HH} = 7.6 Hz, CH_2, 2H), 1.27 (t, J_{HH} = 7.7 Hz, CH_3, 3H). **^{19}F NMR** (376 MHz, d_1-chloroform): δ 17.44 (s, CO*F*, 1F). **HRMS** (ESI): Theoretical $[M]^+$ $[C_9H_9O]^+$ 133.0647; found for $[C_9H_9O]^+$ 133.0652. **IR**(*COF*): 1804 cm^{-1}. [7]

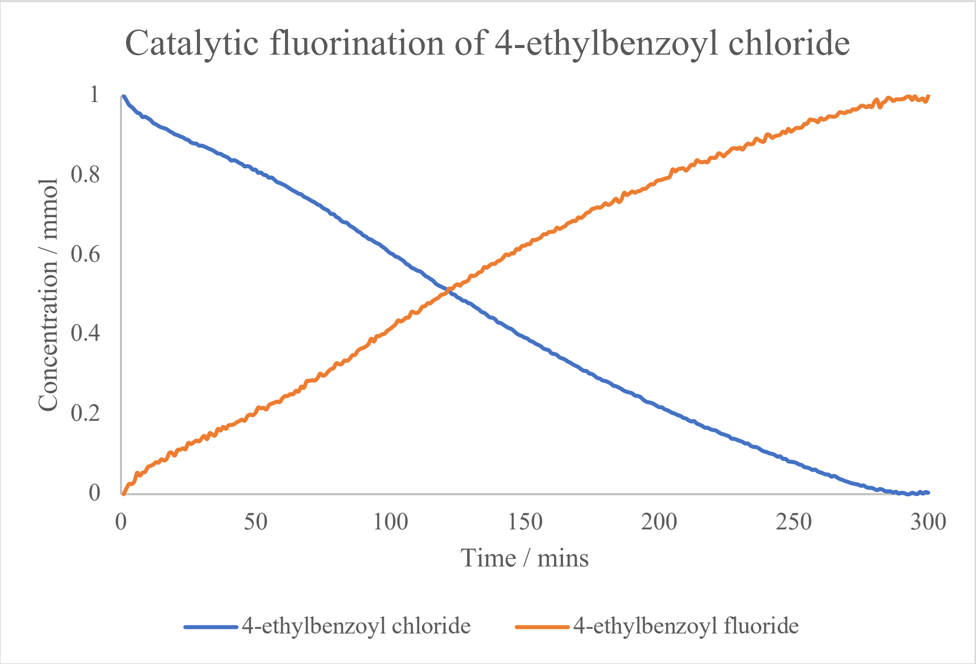

1.3.3.8 Fluorination of 4-^{i}propylbenzoyl chloride

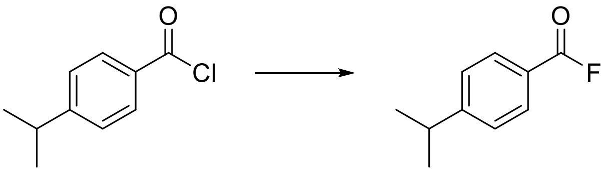

4-^{i}propylbenzoyl fluoride was formed using the general method (*Section* 1.3.3). 4-^{i}propylbenzoyl chloride (1.0 mmol) was added to a Schlenk tube containing **1** (27 mg, 0.05 mmol) and AgF (190 mg, 1.5 eq.) in dry DCM (5 mL), which had previously been stirred for 10 minutes. The reaction was stirred at 20 °C for 220 minutes, until all of the starting material had been consumed, followed by work-up via filtration through celite, removal of solvent and transfer to a NMR tube, alongside deuterated chloroform (0.5 mL) and α,α,α-trifluorotoluene (20 μL). ^{19}F NMR yield *vs*. the internal standard > 99 %, isolated yield of colourless solid, 158 mg, 83 % yield.

^{1}H NMR (400 Mhz, d_1-chloroform): δ 7.96 (d, $^{2}J_{HH}$= 8.3 Hz, C_6-*H,* 2H), 7.37 (d, $^{2}J_{HH}$= 7.9 Hz, C_6-*H,* 2H), 3.00 (sept, $^{3}J_{HH}$= 7.0 Hz, C*H,* 1H), 1.28 (d, $^{3}J_{HH}$= 6.9 Hz, CH_3, 6H).**^{19}F NMR** (376 MHz, d_1-chloroform): δ 17.53 (s, CO*F*, 1F). **HRMS** (ESI): Theoretical [M] $[C_{10}H_{11}O]^+$ 147.0804; found for $[C_{10}H_{11}O]^+$ 147.0803. **IR**(*CO*F): 1800 cm^{-1}.[12]

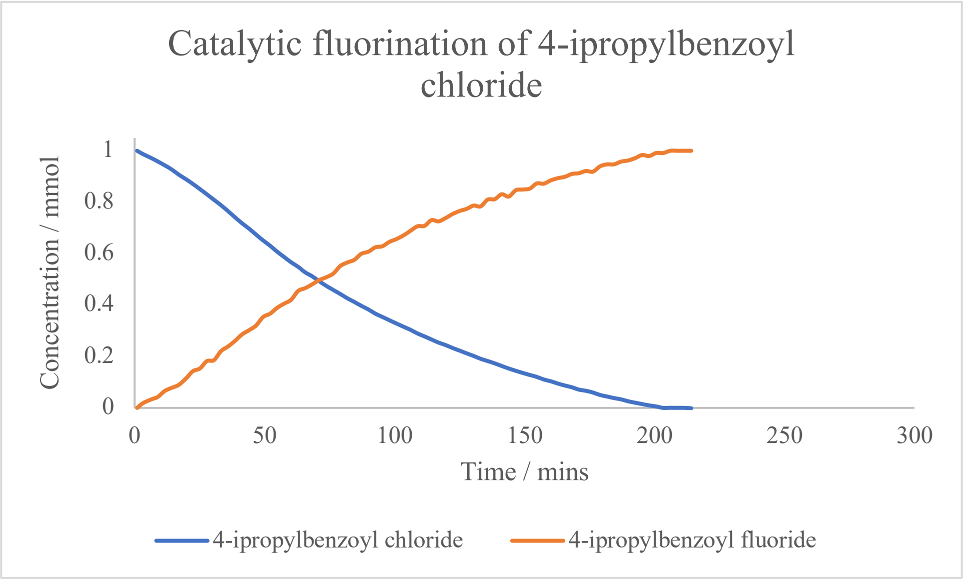

1.3.3.9 *Fluorination of 4-^{t}butylbenzoyl chloride*

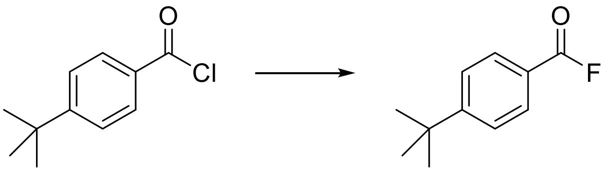

4-^{t}butylbenzoyl fluoride was formed using the general method (*Section* 1.3.3). 4-^{t}butylbenzoyl chloride (1.0 mmol) was added to a Schlenk tube containing **1** (27 mg, 0.05 mmol) and AgF (190 mg, 1.5 eq.) in dry DCM (5 mL), which had previously been stirred for 10 minutes. The reaction was stirred at 20 °C for 230 minutes, until all of the starting material had been consumed, followed by work-up via filtration through celite, removal of solvent and transfer to a NMR tube, alongside deuterated chloroform (0.5 mL) and α,α,α-trifluorotoluene (20 µL). ^{19}F NMR yield *vs*. the internal standard > 99 %, isolated yield of colourless solid, 158 mg, 83 % yield.

^{1}H NMR (400 Mhz, d_1-chloroform): δ 7.98 (d, J_{HH} = 8.5 Hz, C_6-*H*, 2H), δ 7.56 (d, J_{HH} = 7.4 Hz, C_6-*H*, 2H), 1.37 (s, C*H*$_3$, 9H). **^{19}F NMR** (376 MHz, d_1-chloroform): δ 17.47 (s, CO*F*, 1F). **HRMS** (ESI): Theoretical [M] $[C_{11}H_{13}O]^+$ 161.0961; found for $[C_{11}H_{13}O]^+$ 161.0956. **IR**(*COF*): 1805 cm^{-1}.[11]

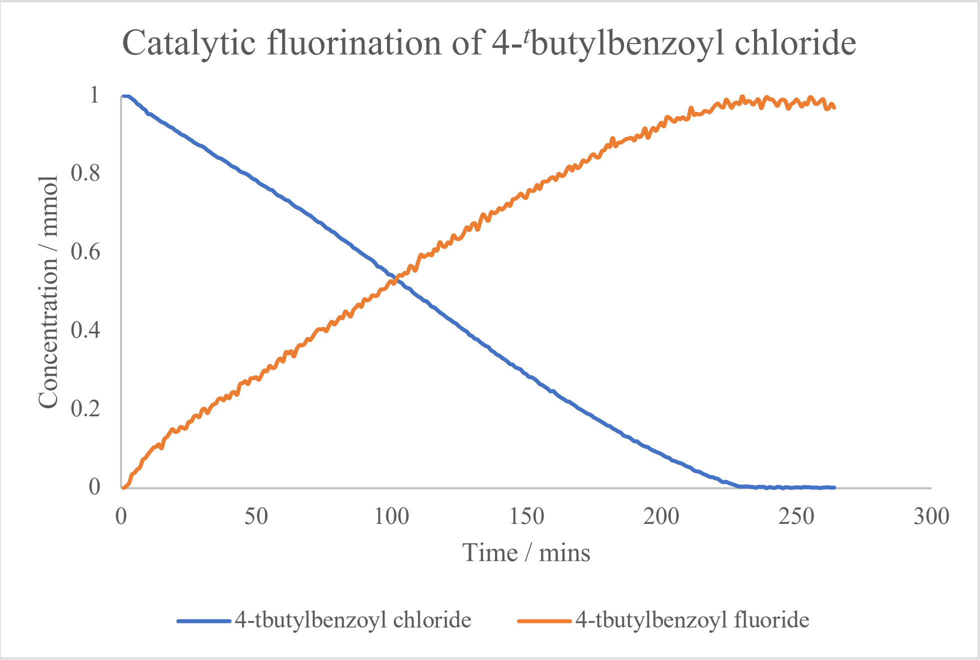

1.3.3.10 Fluorination of 4-methoxybenzoyl chloride

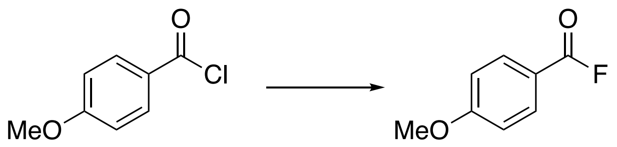

4-methoxybenzoyl fluoride was formed using the general method (*Section* 1.3.3). 4-methoxybenzoyl chloride (1.0 mmol) was added to a Schlenk tube containing **1** (27 mg, 0.05 mmol) and AgF (190 mg, 1.5 eq.) in dry DCM (5 mL), which had previously been stirred for 10 minutes. The reaction was stirred at 20 °C for 620 minutes, until all of the starting material had been consumed, followed by work-up via filtration through celite, removal of solvent and transfer to a NMR tube, alongside deuterated chloroform (0.5 mL) and α,α,α-trifluorotoluene (20 μL). ^{19}F NMR yield *vs*. the internal standard > 99 %, isolated yield of colourless solid, 134 mg, 87 % yield.

^{1}**H NMR** (400 Mhz, d_1-chloroform): δ 8.07 (d, J_{HH} = 8.9 Hz, C_6-*H*, 2H), δ 6.95 (d, J_{HH} = 8.9 Hz, C_6-*H*, 2H), 3.88 (s, C*H*$_3$, 3H). ^{19}**F NMR** (376 MHz, d_1-chloroform): δ 15.89 (s, CO*F*, 1F). ^{13}**C NMR** (101 MHz, d_1-chloroform): δ 167.8 (d, J_{CF} = 747.0 Hz, *C*(O)F),132.4 (s, 2,6-*C*), 121.6 (s, *C*C(O)F), 113.8 (s, 3,5-*C*), 55.5 (s, O*C*H$_3$). **HRMS** (ESI): Theoretical $[M]^+$ $[C_8H_7O_2]^+$ 135.0440; found for $[C_8H_7O_2]^+$ 135.0442. **IR**(*COF*): 1798 cm^{-1}. [7]

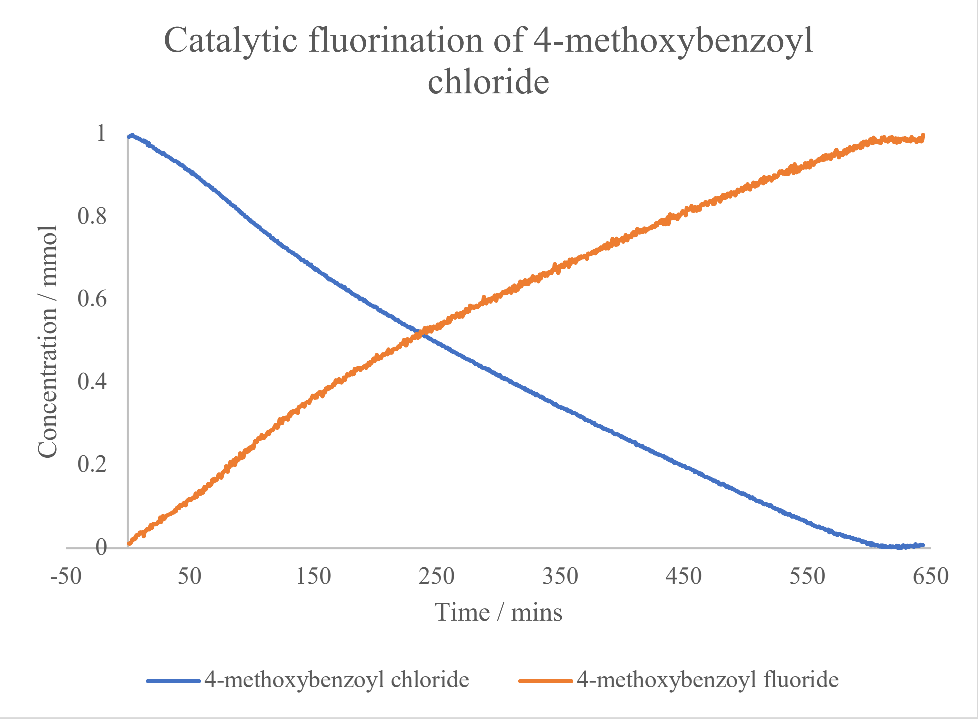

1.3.3.11 Fluorination of probenecid chloride

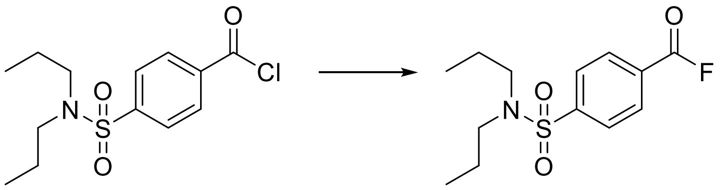

The probenecid fluoride product was formed using the general method (*Section* 1.3.3). Probenecid chloride (1.0 mmol) was added to a Schlenk tube containing **1** (27 mg, 0.05 mmol) and AgF (190 mg, 1.5 eq.) in dry DCM (5 mL), which had previously been stirred for 10 minutes. The reaction was stirred at 20 °C for 90 minutes, until all of the starting material had been consumed, followed by work-up via filtration through celite, removal of solvent and transfer to a NMR tube, alongside deuterated chloroform (0.5 mL) and α,α,α-trifluorotoluene (20 µL). ^{19}F NMR yield *vs*. the internal standard > 99 %, isolated yield of colourless solid, 227 mg, 79 % yield.

^{1}H NMR (400 Mhz, d_1-chloroform): δ 8.10 (dd, J_{HH} = 8.97, 2.4 Hz, C_6-*H*, 2H), 7.89 (dd, J_{HH} = 8.5, 2.3 Hz, C_6-*H*, 2H), 3.05 (tq, J_{HH} = 6.9, 2.3 Hz, N-CH_2, 4H), 1.48 (qt, J_{HH} = 7.4, 2.3 Hz, CH_2, 4H), 0.79 (tt, J_{HH} = 7.5, 2.3 Hz, CH_3, 6H), **^{19}F NMR** (376 MHz, d_1-chloroform): δ 19.91 (s, CO*F*, 1F). **^{13}C NMR** (101 Mhz, d_1-chloroform): δ 156.0 (d, J_{CF} = 345.6 Hz), 146.6, 132.0 (d, J_{CF}= 3.5 Hz, 2,6-*C*), 128.7 (d, J_{CF}= 61.6 Hz, *C*C(O)F), 127.4 (s, 3,5-*C*), 49.8 (s, N*C*H$_2$), 21.8 (s, *C*H$_2$), 10.9 (s, *C*H$_3$). **HRMS** (ESI): Theoretical $[M]^+$ $[C_{13}H_{18}O_3NS]^+$ 268.1001; found for $[C_{13}H_{18}O_3NS]^+$ 268.1002. **IR**(*CO*F): 1816 cm^{-1}. **m.p**: 62-64 ºC.[11]

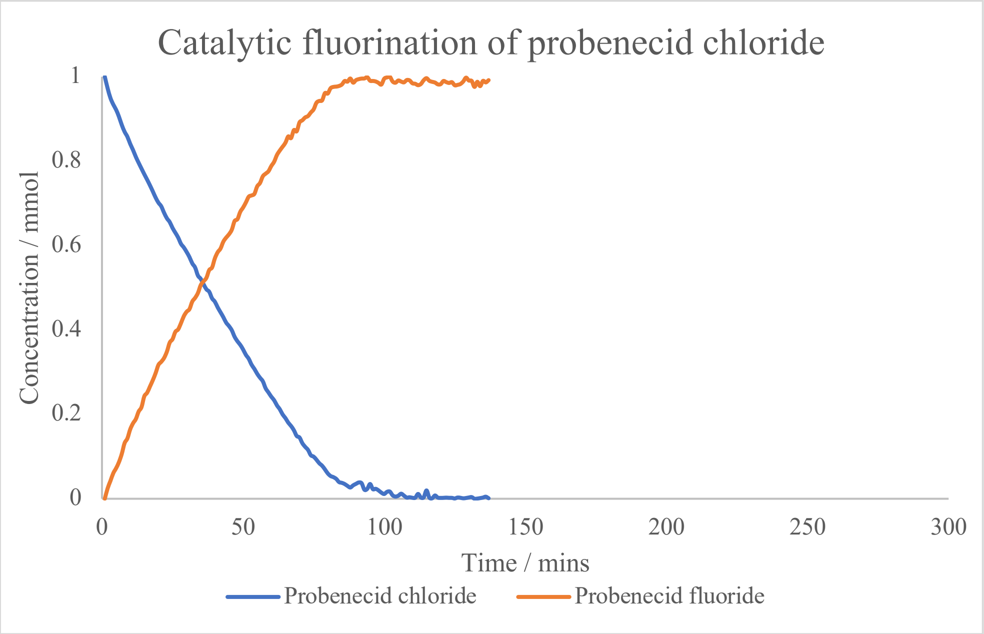

1.3.3.12 Fluorination of benzoic anhydride

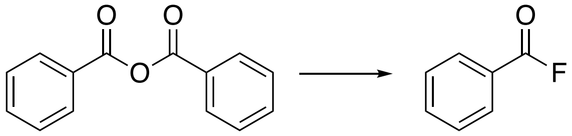

Benzoyl fluoride was formed using the general method (*Section* 1.3.3), substituting the acid chloride for the anhydride. Benzoic anhydride (1.0 mmol) was added to a Schlenk tube containing **1** (27 mg, 0.05 mmol) and AgF (190 mg, 1.5 eq.) in dry DCM (5 mL), which had previously been stirred for 10 minutes. The reaction was stirred at 20 °C for 42 hours, until all of the starting material had been consumed, followed by work-up via filtration through celite, removal of solvent and transfer to a NMR tube, alongside deuterated chloroform (0.5 mL) and α,α,α-trifluorotoluene (20 µL). ^{19}F NMR yield *vs*. the internal standard > 99 %, isolated yield of colourless oil, 108 mg, 87 % yield.

^{1}H NMR (400 Mhz, d_1-chloroform): δ 8.03 (m, C_6-*H,* 2H), 7.70 (tt, J_{HH} = 7.6, 1.2 Hz, C_6-*H*, 1H), 7.52 (m, C_6-*H*, 2H). **^{19}F NMR** (376 MHz, d_1-chloroform): δ 19.69 (s, CO*F*, 1F).[7] **^{13}C NMR** (101 MHz, d_1-chloroform): δ157.4 (d, J_{CF} = 344.2 Hz, *C*(O)F), 135.4 (s, 4-*C*), 131.4 (d, *J* = 4.0 Hz, 2,6-*C*), 129.1 (s, 3,5-*C*), 124.9 (d, J_{CF} = 60.9 Hz, *C*C(O)F). **HRMS** (ESI): Theoretical $[M]^+$ $[C_7H_5O]^+$ 105.0334; found for $[C_7H_5O]^+$ 105.0330. **IR**(*CO*F): 1812 cm^{-1}.[7]

1.4 Visual Time Normalised Analysis

1.4.1 VTN analysis

Figure S1a shows a time normalised concentration profile, where the concentration of the benzoyl chloride reagent has been reduced (Figure S1a, green). The lower concentration of starting material has undergone time normalisation and been shifted to overlay at [benzoyl chloride]$_{1/2}$. As can be seen in Figure S1a, the time normalised concentration profiles for both 1 mmol and 0.5 mmol starting concentration of benzoyl chloride overlay closely. This indicated that over the time course of the reaction at the sampled concentrations no product inhibition or catalyst deactivation is occurring.

Next, the effect of catalyst activation was investigated. Under normal conditions, the catalyst and silver fluoride are stirred for 10 minutes prior to addition of the acyl chloride reagent. This was conducted to generate the active catalytic species from the pre-catalyst, **1**. The effect of catalyst activation was probed, by following the start of the fluorination of benzoyl chloride, upon immediate addition of the substrate and addition of the substrate after 10 minutes equilibration. It can be observed in Figure S1b that the initial rate of reaction between these two systems do not overlay, with an increased rate observed when time was allowed for catalyst pre-activation (Figure S1b; orange) compared to under non-standard conditions, where catalyst pre-activation was not allowed to occur prior to addition of the acyl chloride substrate (Figure S1b; blue). After this period of increased initial rate at the beginning of the reaction post catalyst activation the rate of fluorination runs parallel to the non-preactivated reaction. Where excess silver fluoride was used, it was also possible to see the effect of catalyst activation in real time (Figure S1c). Addition of a second aliquot of benzoyl chloride following consumption of the first aliquot of substrate, in which no catalyst pre-activation occurred, led to a faster initial rate of reaction than the addition of the first aliquot.

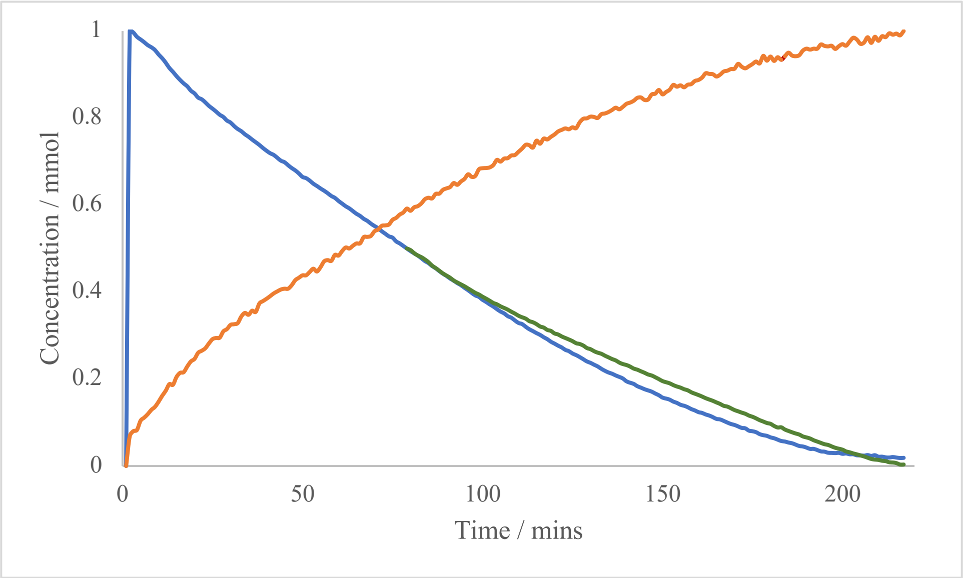

Figure S1a. Time normalized concentration profile for the catalytic fluorination of benzoyl chloride, at [benzoyl chloride]$_{1.0\ mmol}$ (blue), [benzoyl chloride]$_{0.5\ mmol}$. [Benzoyl fluoride] given for the formation of product from 1.0 mmol benzoyl chloride.

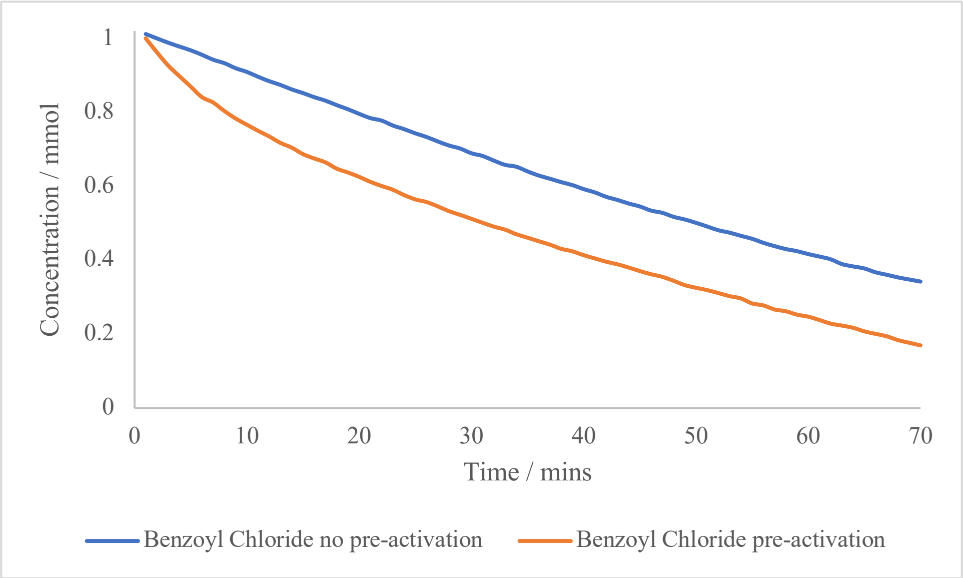

Figure S1b. The effect of catalyst pre-activation on the initial rate of consumption of benzoyl chloride, 10 minutes of catalyst pre-activation (orange) and no catalyst pre-activation (blue).

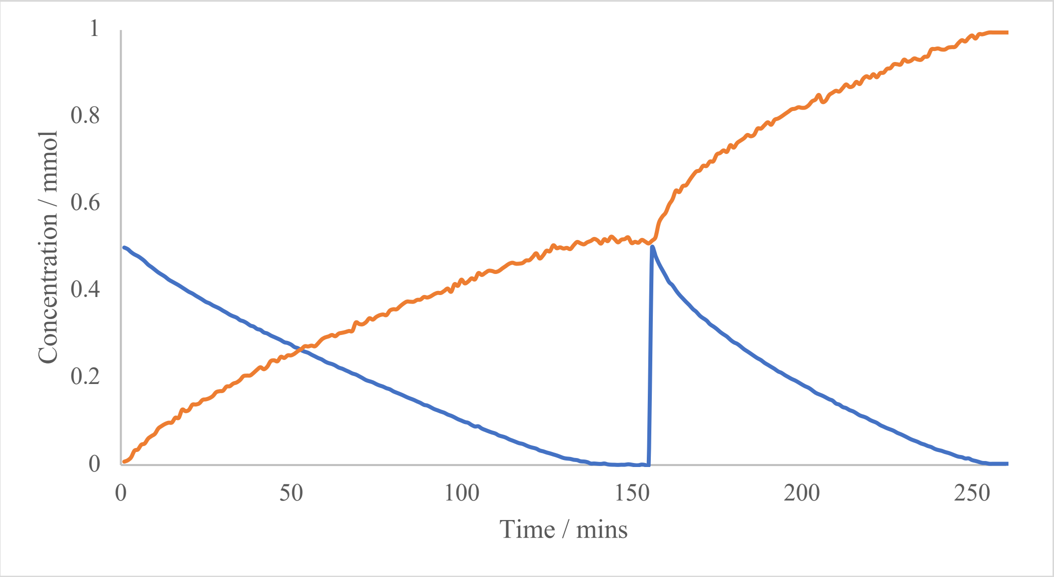

Figure S1c. Two separate additions of benzoyl chloride (0.5 mmol, blue) reveals the effect of catalyst activation on the rate of formation of benzoyl fluoride (orange).

1.4.2 Rate order calculation from VTNA analysis

Rate order determination by visual time normalized kinetic analysis was developed by Bures and co-workers.[13] To elucidate the order of the different components within the reaction the effects of the concentration of these components were examined. The reaction profile for the catalytic fluorination of benzoyl chloride, generated through *in-situ* FTIR analysis, at different concentrations of the substrate were compared. Table S1 presents the results at different concentrations of benzoyl chloride where all other conditions were kept the same. To enable comparison of the reaction profiles at 1.0 mmol benzoyl chloride and 0.5 mmol benzoyl chloride it is necessary to shift the reaction profiles so that the starting point of the reaction is the same (i.e. [benzoyl chloride]$_{1/2}$ at 1.0 mmol). This normalized concentration-time scale for both reactions allow for the order of reaction with respect to benzoyl chloride to be determined. Overlay at different [benzoyl chloride] can be obtained by plotting the normalized concentration of substrate, [benzoyl chloride] against the time normalized concentration profile, tA, which can be calculated using the following equation.

$$tA = \sum(\Delta[benzoyl\ chloride])^{\alpha(\Delta t)}$$

where; α = rate order

The rate order (α) in the equation above can be arbitrarily changed from 0 to *n*, where *n* is the rate order. The correct rate order is determined when the concentration-time normalized profiles for [benzoyl chloride] at 1.0 mmol and [benoyl chloride] at 0.5 mmol overlay. Changing the rate order in

substrate resulted in an overlay between the concentration-time normalised plot at rate order 1 (Figure S2).

To determine the rate order with respect to catalyst, two reactions with identical initial concentration of benzoyl chloride, but different initial concentration of catalyst were compared. The normalized concentration-time profile was used as described above, where the effect on the change in [cat.], *t*cat, on the [benzoyl chloride] was examined (Table S2). An assumption of constant concentration of active catalyst in solution is applied. *T*cat is given by the following equation;

$$tcat = \sum(\Delta[catalyst])^{\alpha(\Delta t)}$$

Applying a rate order in catalyst of 1, was observed to give the closest overlay between the concentration-time normalized profiles (Figure S3), however precise overlay was not observed, signifying added complexity within the system.

Table S1. Rate order calculation for the catalytic fluorination of benzoyl chloride with respect to substrate.

1.0 mmol Benzoyl Chloride			0.5 mmol Benzoyl Chloride		
t (min)	tA	[benzoyl chloride]	t (min)	tA	[benzoyl chloride]
0	0.00	1.00	0	0.00	1.00
12	11.44	0.91	9	8.54	0.90
25	24.44	0.81	18	23.88	0.81
41	40.44	0.71	30	41.97	0.70
59	58.44	0.60	42	57.64	0.60
76	75.44	0.51	55	72.10	0.51
86	85.44	0.45	63	79.74	0.45

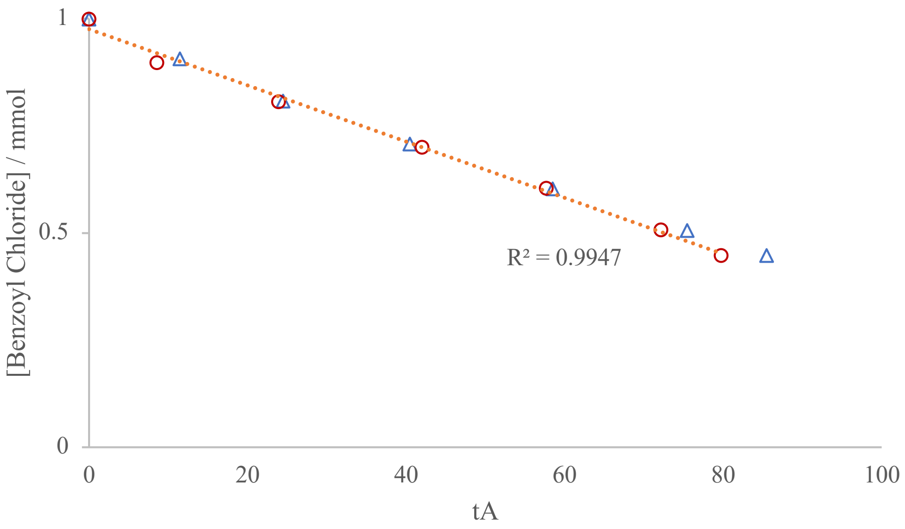

Figure S2. Rate order calculation with respect to substrate, taken from concentration-time normalized data in Table S1. Overlay was observed upon defining rate order with respect to substrate as 1.

Table S2. Rate order calculation for the catalytic fluorination of benzoyl chloride with respect to catalyst.

5 mol % catalyst				10 mol % catalyst			
t (min)	tcat	[benzoyl chloride]	[cat.]	t (min)	tcat	[benzoyl chloride]	[cat.]
**0**	0.00	1.00	0.05	0	0.00	1.00	0.10
**12**	6.00	0.91	0.05	9	0.90	0.90	0.10
**25**	12.50	0.81	0.05	18	9.90	0.81	0.10
**41**	20.50	0.71	0.05	30	19.20	0.70	0.10
**59**	29.50	0.60	0.05	42	31.20	0.60	0.10
**76**	38.00	0.51	0.05	55	43.30	0.51	0.10
**86**	43.00	0.45	0.05				

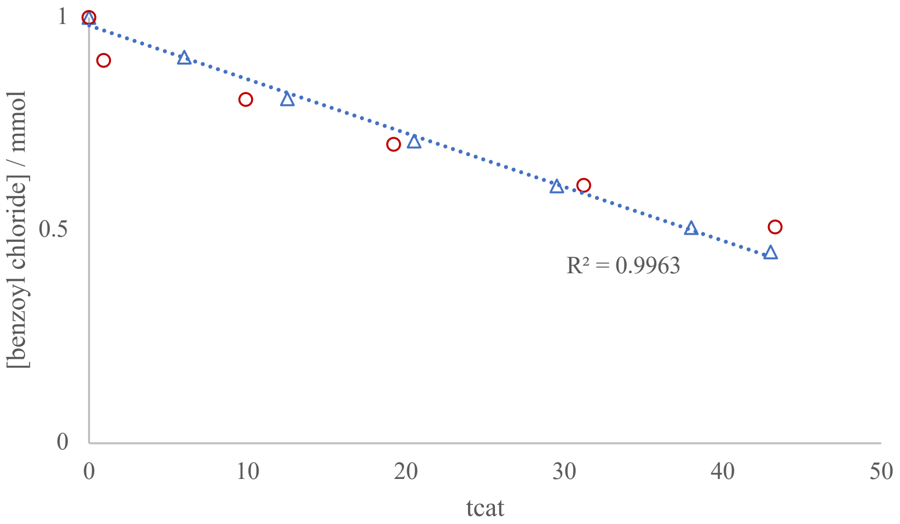

Figure S3. Rate order calculation with respect to catalyst, taken from concentration-time normalized data in Table . Closest overlay was observed upon defining rate order with respect to catalyst as 1.

1.4.3 Initial rate calculations

The initial reaction rates for the fluorination of the acyl chloride substrates were calculated by comparing the change in the concentration of the acyl chloride substrate, determined from the concentration normalized reaction profile taken from ReactIR analysis, over the initial 20 minutes of the reaction, following substrate addition. The acyl chloride substrate was added after catalyst pre-activation of stirring **1** with AgF for 10 minutes. This initial rate of reaction (mmolh^{-1}) was then compared against the carbonyl stretching frequency (cm^{-1}) of the acyl chloride substrates (Table S3).

Table S3. Initial rates for the catalytic fluorination of acyl chloride substrates against carbonyl stretching frequency.

Substrate	*C(O)*Cl wavenumber /cm^{-1}	Initial Rate / $mmolh^{-1}$
**4-nitrobenzoyl chloride**	1788	0.974
**Probenecid chloride**	1782	0.942
**4-butylbenzoyl chloride**	1778	0.511
**Benzoyl chloride**	1775	0.483
**4-ethybenzoyl chloride**	1774	0.305
**Toluoyl chloride**	1773	0.513
**4-isopropylbenzoyl chloride**	1771	0.348
**4-methoxybenzoyl chloride**	1765	0.038

1.4.4 Calibration curve

Off-line ^{19}F NMR analysis was conducted following the completion of the catalytic fluorination of acyl chlorides, to determine the contained yield of the acyl fluoride product. The integration of the C(O)*F* peak was compared to a known quantity of the internal standard, trifluorotoluene (- 63.72 ppm). A calibration curve was generated by comparing known quantities of pure benzoyl fluoride, purchased from Merck, against the internal standard. The calculated standard deviation gave an associated error of contained yield at ±1 %.

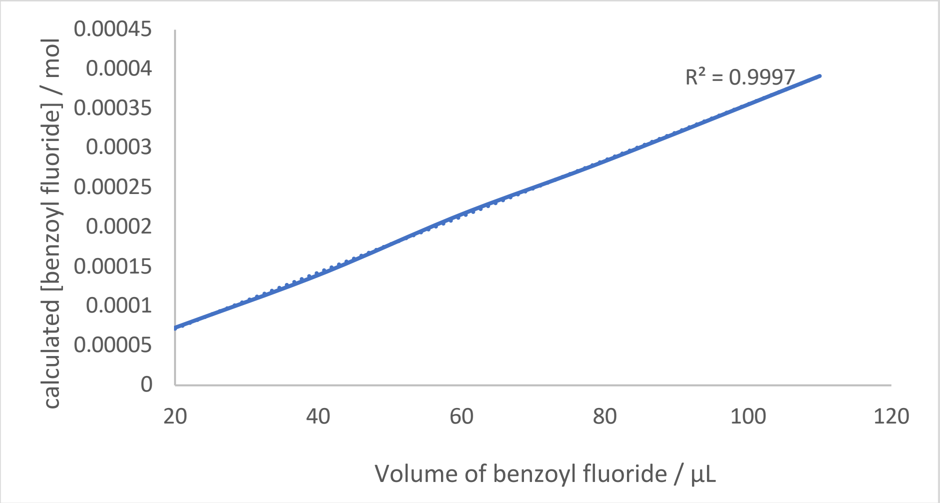

Figure S4. Calibration curve for the concentration of benzoyl chloride vs calculated ^{19}F NMR concentration against internal standard, α,α,α-trifluorotoluene (20 µL). Standard deviation gives an associated error of ±1 %.

1.5 Computations

1.5.1 Computational details

DFT calculations were run with Gaussian 09 (Revision D.01).[14] Geometry optimisations were performed with the BP86 functional[15,16] with Rh and Cl centers described with the Stuttgart RECPs and associated basis sets,[17] with additional d-orbital polarisation on Cl (ζ = 0.640)[18] and 6-31G** basis sets were used for all other atoms.[19,20] This basis set combination is termed BS1. All optimisations were performed taking into account the dichloromethane solvent (ε = 8.93) via the PCM approach[21] and the ‘grid=ultrafine’ option was used throughout. All stationary points were fully characterized via analytical frequency calculations as either minima (all positive eigenvalues) or transition states (one negative eigenvalue). IRC calculations and subsequent geometry optimizations were used to confirm the minima linked by each transition state. All electronic energies were recomputed with the wB97x-D functional[22] and a def2-TZVP basis set for all atoms (BS2).[23,24] These electronic energies were then combined with the thermochemical corrections from the frequency analyses of the geometries optimised in dichloromethane solvent with BS1 to give the final free energies quoted in the text.

Four possible pathways for the nucleophilic attack of the Rh-F ligand at the acyl chloride were considered with the C=O group oriented ‘up’ or ‘down’ and ‘left’ or right’ with respect to the Rh-F vector. Transition states were located for all four orientations but only the most stable pathways are reported here.

1.5.2 Computed stationary points and labelling scheme

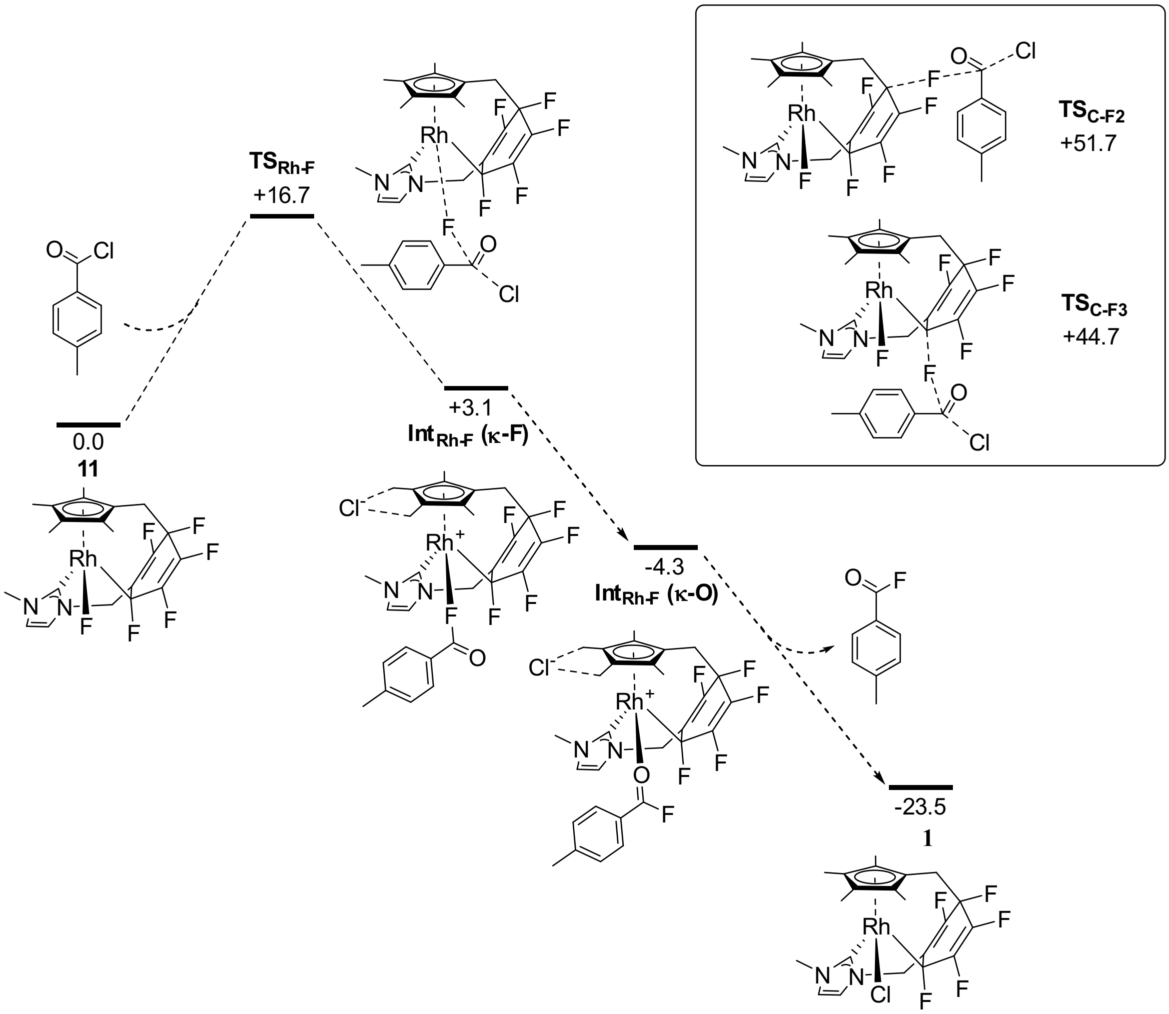

Figure S5. Computed free energies (kcal/mol) and labelling scheme for stationary points for the reactions of **11** with toluyl chloride. Inset shows the much higher alternative transitions states for attack via the C–F2 and C–F3 bonds.

1.6 References

1. McGrandle, S.; Saunders, G. C. Group 9 Complexes of an N-Heterocycle Carbene Bearing a Pentafluorobenzyl Substituent: Attempted Dehydrofluorinative Coupling of Cyclopentadienyl and N-Heterocycle Carbene Ligands. *J. Fluor. Chem.* **2005**, *126*, 449–453.
2. Ma, Y.; Wang, Y.-M.; Morgan, P. J.; Jackson, R. E.; Liu, X.; Saunders, G. C.; Lorenzini, F.; Marr, A. C. Designing Effective Homogeneous Catalysis for Glycerol Valorisation: Selective Synthesis of a Value-Added Aldehyde from 1,3-Propanediol via Hydrogen Transfer Catalysed by a Highly Recyclable, Fluorinated Cp*Ir(NHC) Catalyst. *Catal. Today* **2018**, *307*, 248–259.
3. Thomas, H. P.; Marr, A. C.; Morgan, P. J.; Saunders, G. C. Tethering of Pentamethylcyclopentadienyl and N-Heterocycle Stabilized Carbene Ligands by Intramolecular 1,4-Addition to a Polyfluorophenyl Substituent. *Organometallics* **2018**, *37*, 1339–1341.
4. C. White; A. Yates; Maitlis, P. M., Pentamethylcyclopentadienylrhodium and iridium compounds. *Inorg. Syn.* **1992,** *29*, 228 -234.
5. Dmowski, W.; Kamiński, M. Dialkyl-∝-Difluorobenzylamines and Dialkyl(Trifluoromethyl)-Amines - Novel Fluorinating Reagents. *J. Fluor. Chem.***1983**, *23*, 219–228.
6. Hutchinson, G.; Welsh, C. D. M.; Bures, J., Use of Standard Addition to Quantify In Situ FTIR Reaction Data. *J. Org. Chem.* **2021,** *86* (2), 2012-2016.
7. Munoz, S. B.; Dang, H.; Ispizua-Rodriguez, X.; Mathew, T.; Prakash, G. K. S. Direct Access to Acyl Fluorides from Carboxylic Acids Using a Phosphine/Fluoride Deoxyfluorination Reagent System. *Org. Lett.* **2019**, *21*, 1659–1663.
8. Leclerc, M. C.; Bayne, J. M.; Lee, G. M.; Gorelsky, S. I.; Vasiliu, M.; Korobkov, I.; Harrison, D. J.; Dixon, D. A.; Baker, R. T. Perfluoroalkyl Cobalt(III) Fluoride and Bis(Perfluoroalkyl) Complexes: Catalytic Fluorination and Selective Difluorocarbene Formation. *J. Am. Chem. Soc.* **2015**, *137*, 16064–16073.
9. Scattolin, T.; Deckers, K.; Schoenebeck, F. Direct Synthesis of Acyl Fluorides from Carboxylic Acids with the Bench-Stable Solid Reagent $(Me_4N)SCF_3$. *Org. Lett.* **2017**, *19*, 5740–5743.
10. Shipilov A. I.; Kolpashchikova, L. A.; Igumnov, S. M., Selective Hydrolysis of Pentafluorobenzotrichloride. *Russ J Org Chem* **2003,** *39*, 975-978.
11. Malapit, C. A.; Bour, J. R.; Brigham, C. E.; Sanford, M. S., Base- free nickel-catalysed decarbonylative Suzuki-Miyaura coupling of acid fluorides. *Nature* **2018,** *563*, 100-104.
12. Meanwell, M.; Lehmann, J.; Eichenberger, M.; Martin, R. E.; Britton, R., Synthesis of acyl fluorides via photocatalytic fluorination of aldehydic C-H bonds. *Chem Commun* **2018,** *54*, 9985-9988.
13. Nielsen, C. D.; Bures, J., Visual kinetic analysis. *Chem. Sci.* **2019,** *10* (2), 348-353
14. Gaussian 09, Revision D.01, M. J. Frisch, G. W. Trucks, H. B. Schlegel, G. E. Scuseria, M. A. Robb, J. R. Cheeseman, G. Scalmani, V. Barone, B. Mennucci, G. A. Petersson, H. Nakatsuji, M. Caricato, X. Li, H. P. Hratchian, A. F. Izmaylov, J. Bloino, G. Zheng, J. L. Sonnenberg, M. Hada, M. Ehara, K. Toyota, R. Fukuda, J. Hasegawa, M. Ishida, T. Nakajima, Y. Honda, O. Kitao, H. Nakai, T. Vreven, J. A. Montgomery, J. E. Peralta, F. Ogliaro, M. Bearpark, J. J. Heyd, E. Brothers, K. N. Kudin, V. N. Staroverov, R. Kobayashi, J. Normand, K. Raghavachari, A. Rendell, J. C. Burant, S. S. Iyengar, J. Tomasi, M. Cossi, N. Rega, J. M. Millam, M. Klene, J. E. Knox, J. B. Cross, V. Bakken, C. Adamo, J. Jaramillo, R. Gomperts, R. E. Stratmann, O. Yazyev, A. J. Austin, R. Cammi, C. Pomelli, J. W. Ochterski, R. L. Martin, K. Morokuma, V. G. Zakrzewski, G. A. Voth, P. Salvador, J. J. Dannenberg, S. Dapprich, A. D. Daniels, Farkas, J. B. Foresman, J. V. Ortiz, J. Cioslowski and D. J. Fox, Gaussian Inc., Wallingford CT, **2013**.
15. Density-functional exchange-energy approximation with correct asymptotic behavior. A. D. Becke, *Phys. Rev. A*, **1988**, *38*, 3098-3100.
16. Density-functional approximation for the correlation energy of the inhomogeneous electron gas. J. P. Perdew, *Phy. Rev. B*, **1986**, *33*, 8822-8824.

17. Energy-adjusted ab initio pseudopotentials for the second and third row transition elements. D. Andrae, U. Häußermann, M. Dolg, H. Stoll and H. Preuß, *Theor. Chim. Acta*, **1990**, *77*, 123-141.
18. A set of d-polarization functions for pseudo-potential basis sets of the main group elements Al-Bi and f-type polarization functions for Zn, Cd, Hg. A. Höllwarth, M. Böhme, S. Dapprich, A. W. Ehlers, A. Gobbi, V. Jonas, K. F. Köhler, R. Stegmann, A. Veldkamp and G. Frenking, *Chem. Phys. Lett.* **1993**, *208*, 237-240
19. Self-Consistent Molecular Orbital Methods. XII. Further Extensions of Gaussian-Type Basis Sets for Use in Molecular Orbital Studies of Organic Molecules. W. J. Hehre, R. Ditchfield and J. A. Pople, *J. Chem. Phys.* **1972**, *56*, 2257-2261.
20. The influence of polarization functions on molecular orbital hydrogenation energies. P. C. Hariharan and J. A. Pople, *Theor.l Chim. Acta*, **1973**, *28*, 213-222.
21. Quantum Mechanical Continuum Solvation Models. J. Tomasi, B. Mennucci and R. Cammi, *Chem. Rev.* **2005**, *105*, 2999-3094.
22. Long-range corrected hybrid density functionals with damped atom–atom dispersion corrections. J.-D. Chai and M. Head-Gordon, *Phys. Chem. Chem. Phys.* **2008**, *10,* 6615-6620.
23. Accurate Coulomb-fitting basis sets for H to Rn. F. Weigend, *Phys. Chem. Chem. Phys.* **2006**, *8*, 1057-1065.
24. Balanced basis sets of split valence, triple zeta valence and quadruple zeta valence quality for H to Rn: Design and assessment of accuracy. F. Weigend and R. Ahlrichs, *Phys. Chem. Chem. Phys.* **2005**, *7*, 3297-3305.

2 Characterisation

Figure S6.^{19}F NMR spectrum of the recovered catalyst, pre-regeneration with Ag_2O.

^{19}F NMR (376 MHz, Chloroform-*d*) δ -140.28 (dd, *J* = 20.9, 6.7 Hz), -150.13 (t, *J* = 20.9 Hz), -159.45 – -159.72 (m).

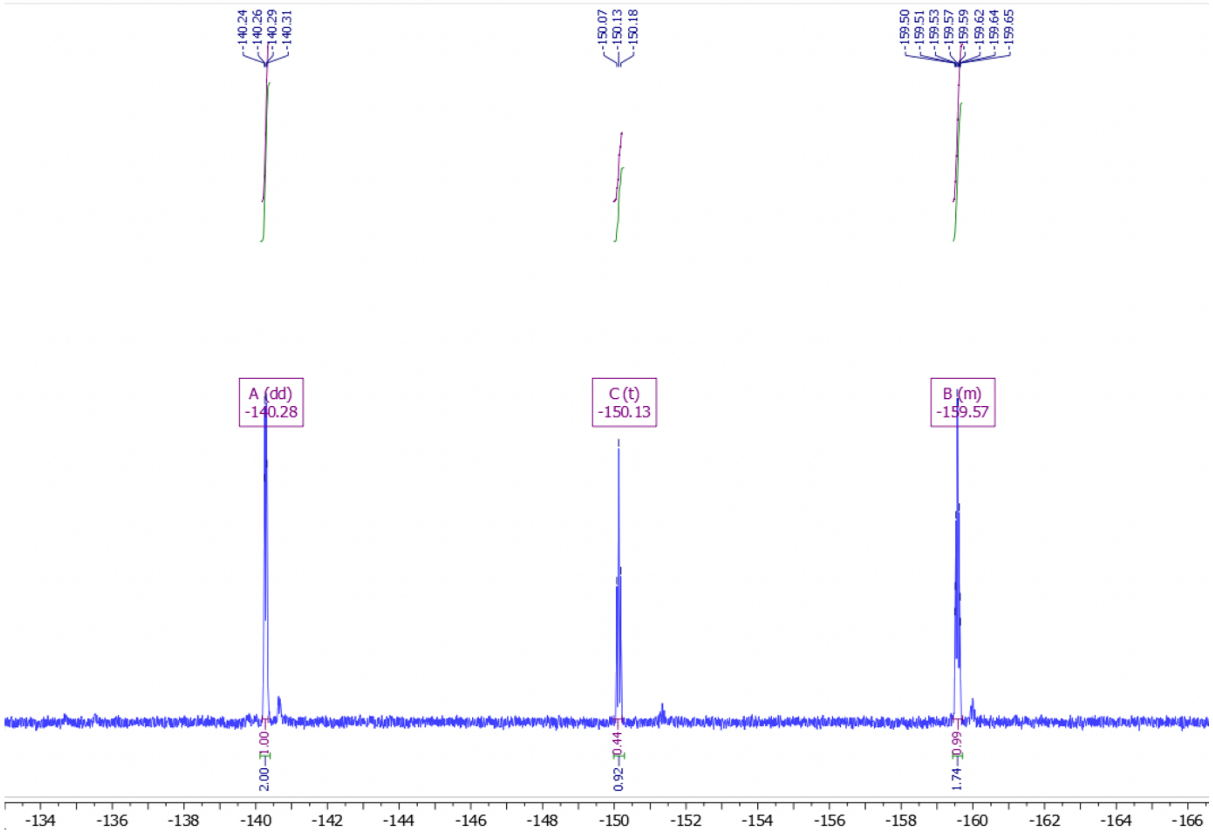

Figure S7. ^{19}F NMR spectrum of the recovered catalyst 1, post-regeneration with Ag_2O.

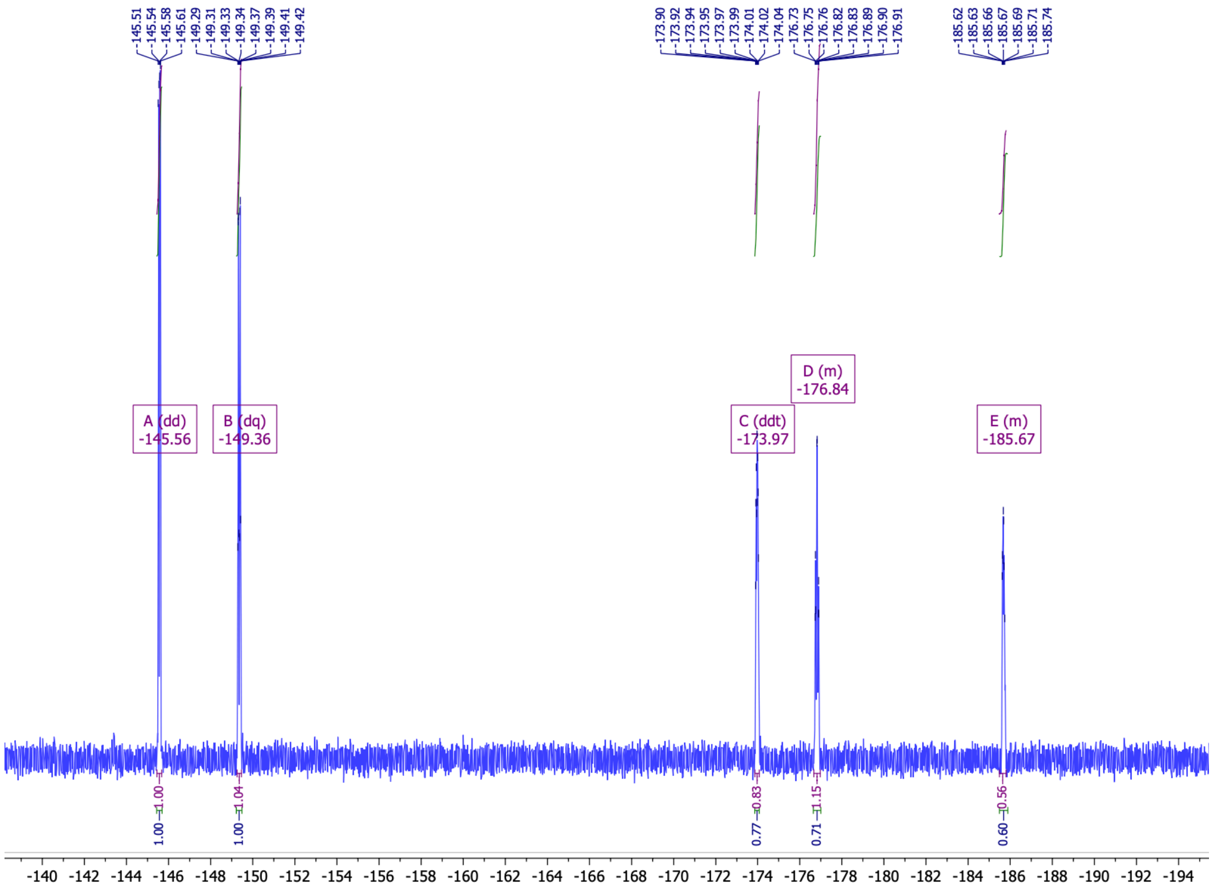

Figure S8. NMR spectra of benzoyl fluoride

^{1}H NMR (400 MHz, Chloroform-*d*) δ 8.02 (dd, *J* = 7.5, 1.0 Hz, 2H), 7.69 (tt, *J* = 7.5, 1.3 Hz, 1H), 7.51 (dd, *J* = 8.3, 1.1 Hz, 2H).

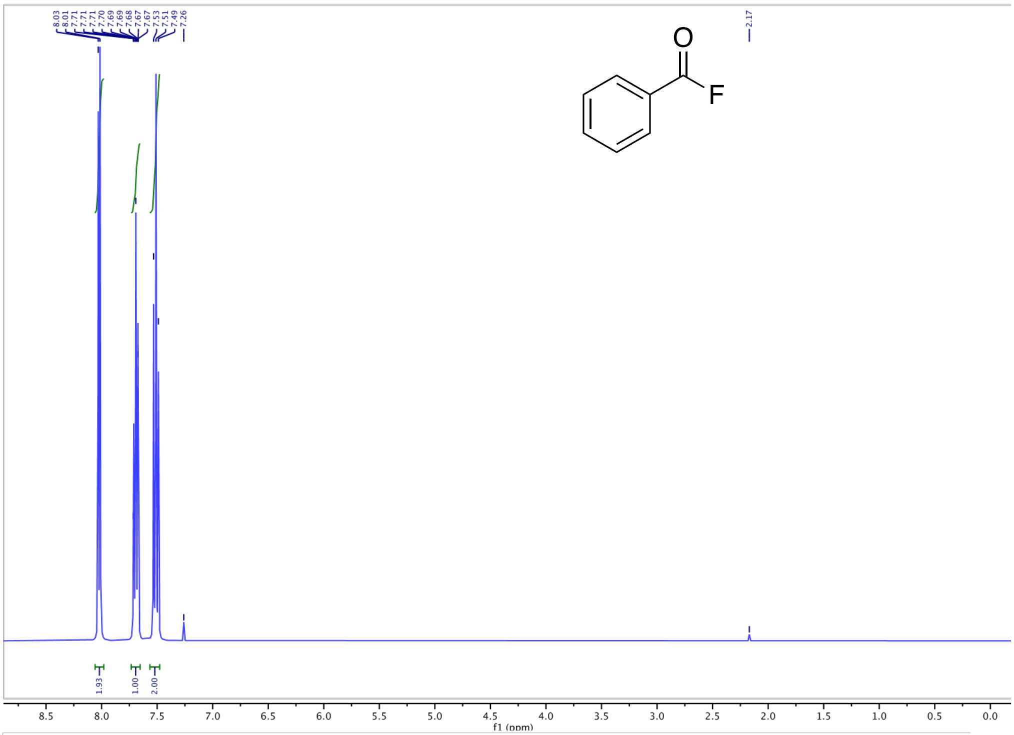

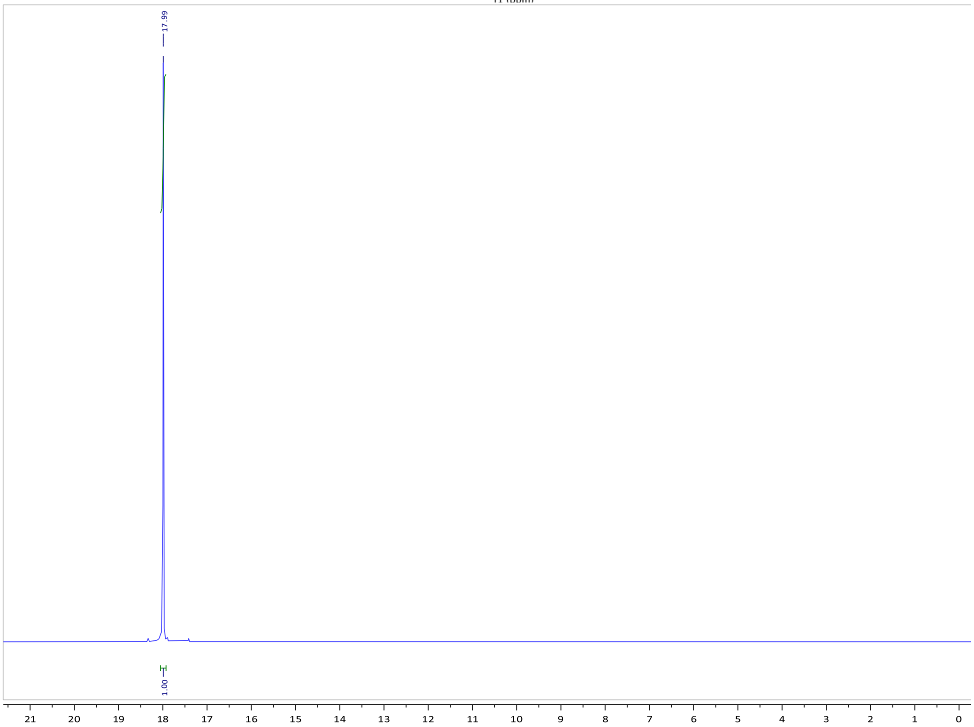

^{13}C NMR (101 MHz, Chloroform-*d*) δ 157.43 (d, *J* = 344.2 Hz), 135.37, 131.42 (d, *J* = 4.0 Hz), 129.10, 124.94 (d, *J* = 60.9 Hz).

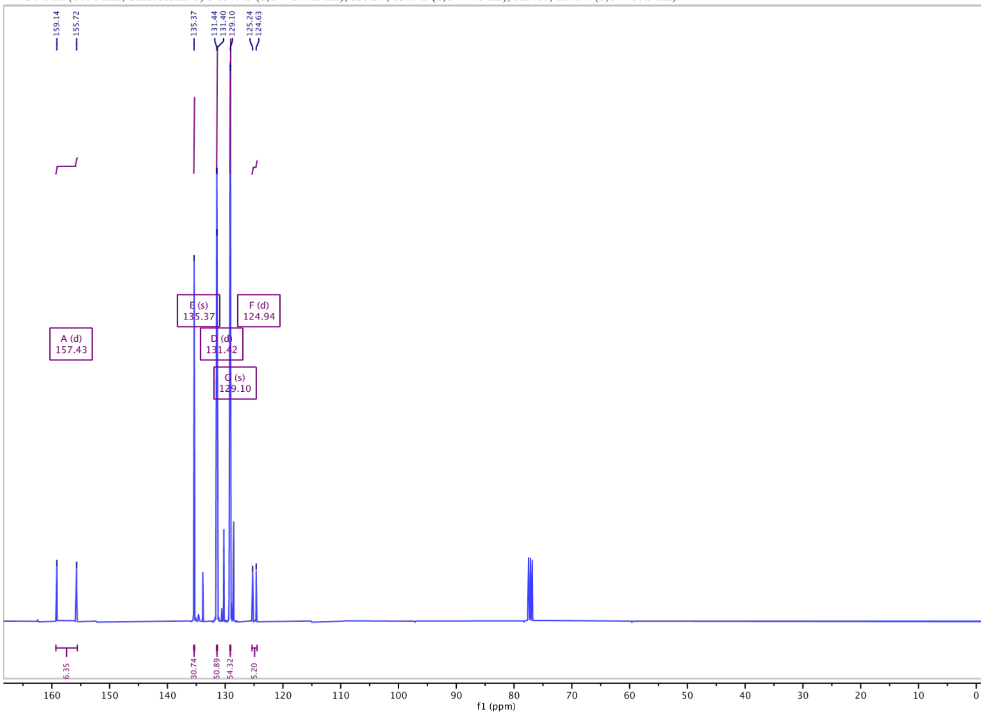

Figure S9. NMR spectra of toluoyl fluoride.

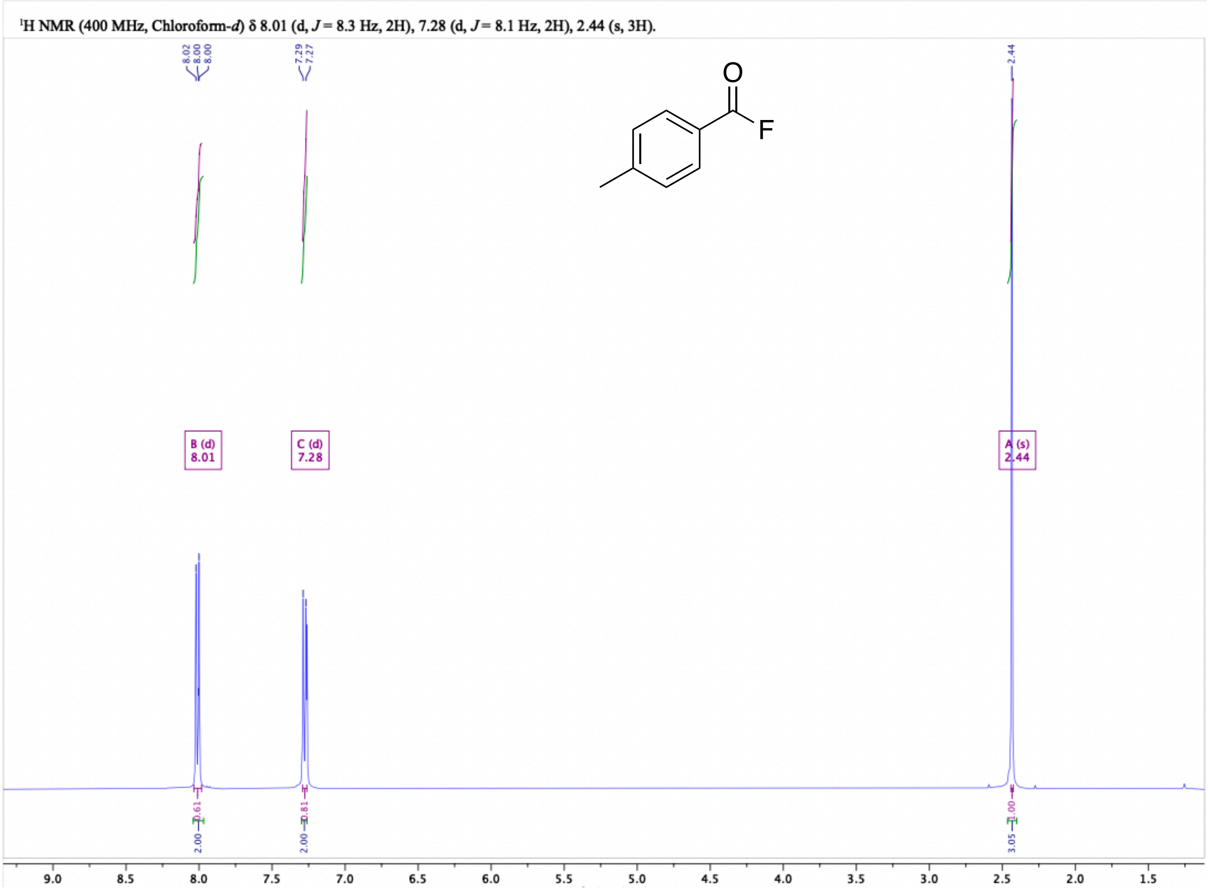

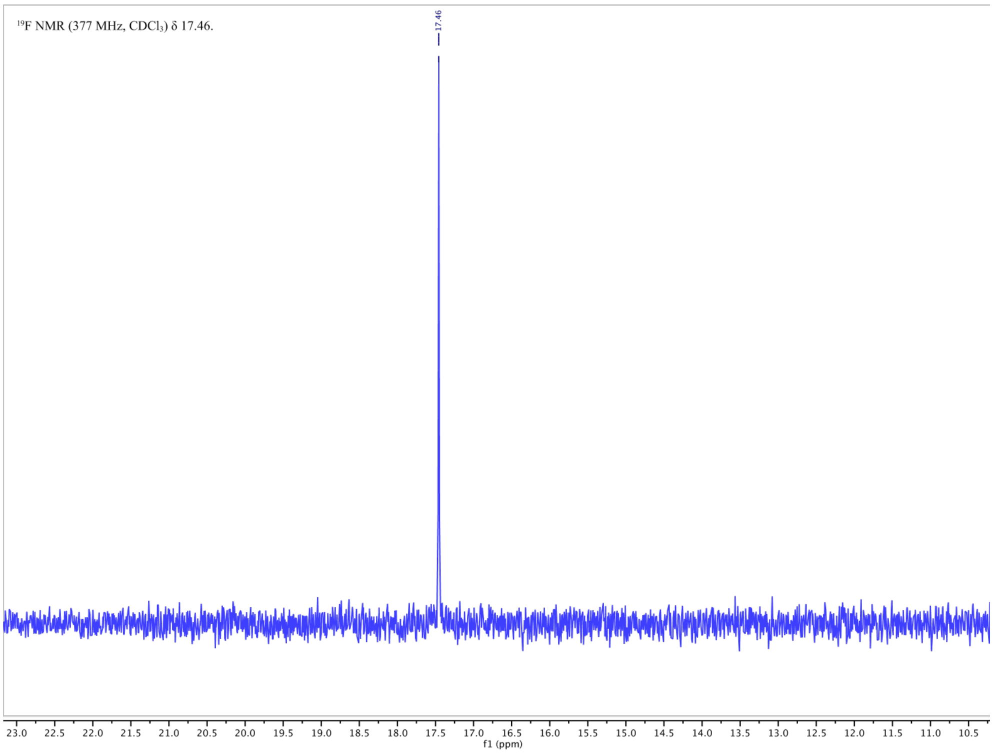

Figure S10. NMR spectra of 4-nitrobenzoyl fluoride.

^{1}H NMR (400 MHz, $CDCl_3$) δ 8.38 (d, J = 7.8 Hz, 1H), 8.25 (d, J = 8.8 Hz, 1H).

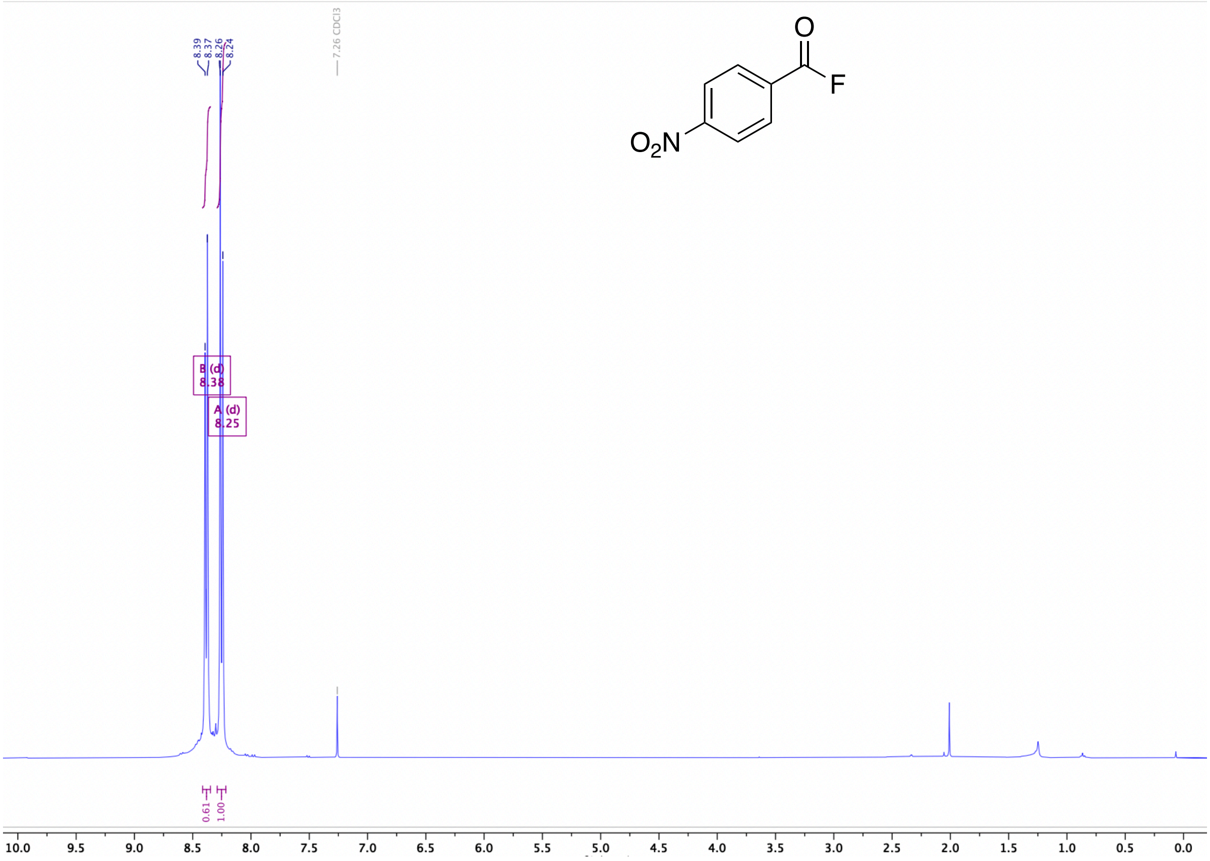

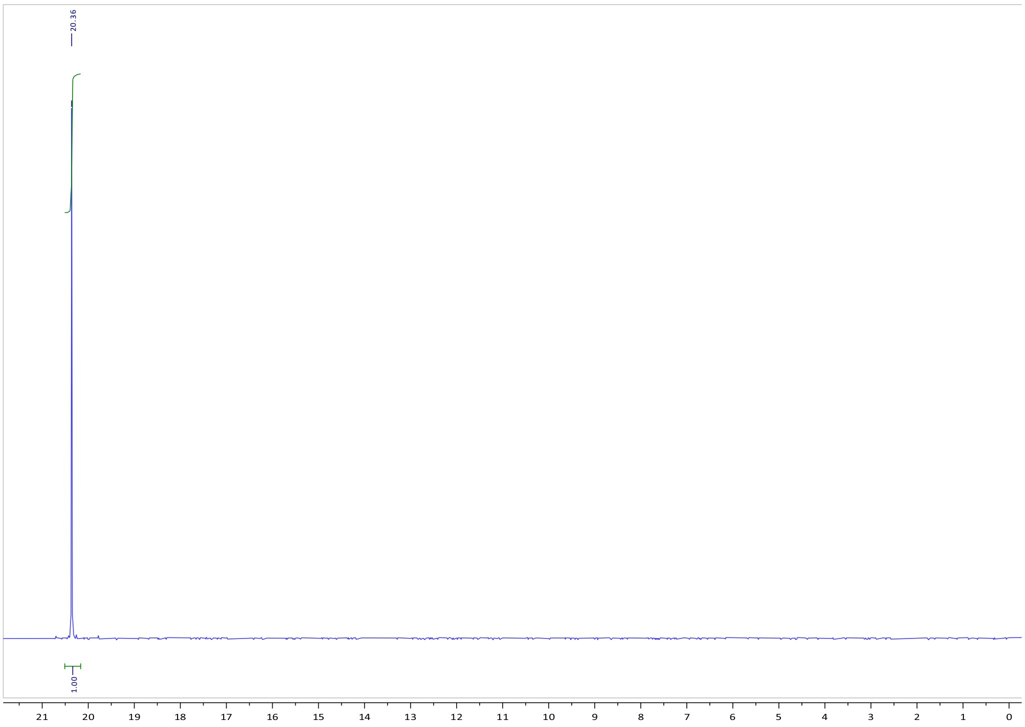

^{13}C NMR (101 MHz, $CDCl_3$) δ 155.6 (d, J = 346.4 Hz), 152.0, 132.7 (d, J = 3.5 Hz), 130.4 (d, J = 63.2 Hz), 124.3.

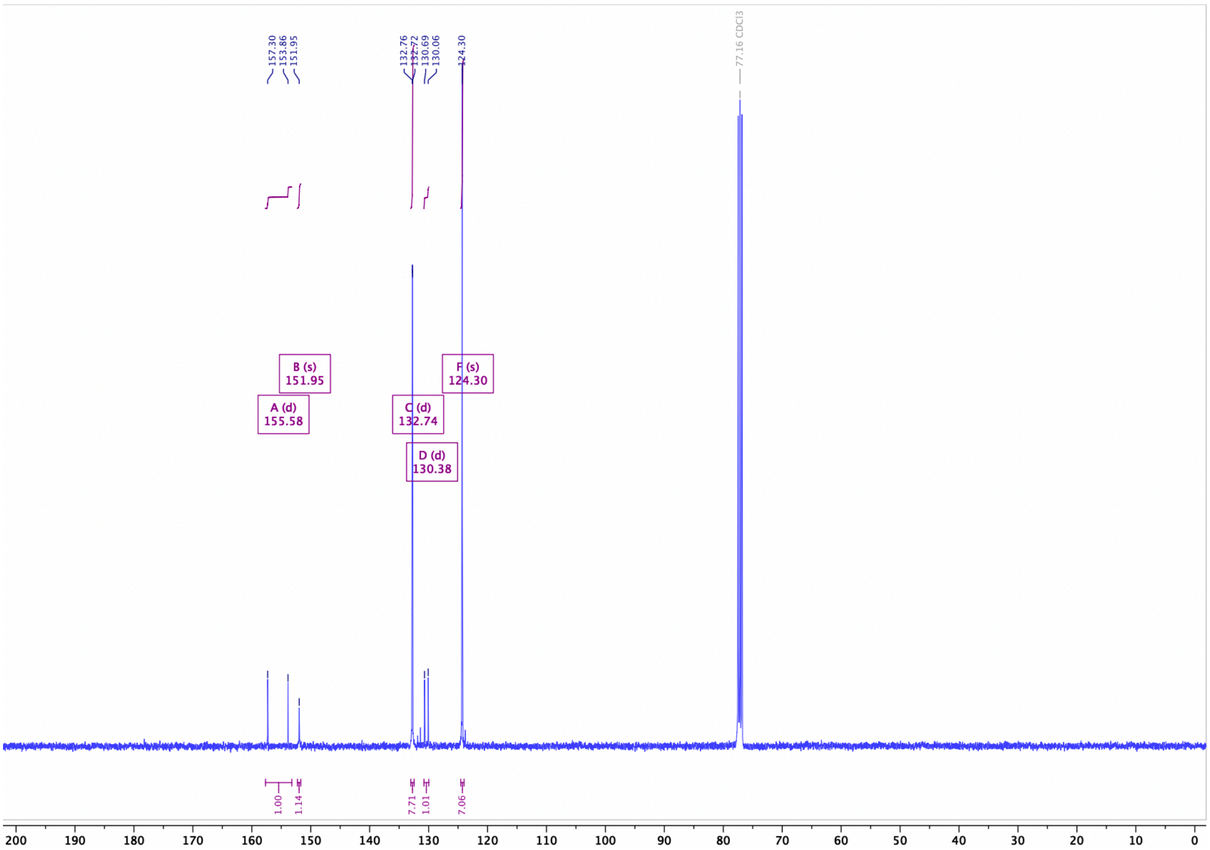

Figure S11. NMR spectra of 2,6-difluorobenzoyl fluoride.

^{1}H NMR (400 MHz, Chloroform-*d*) δ 7.48 (tt, *J* = 8.5, 6.1 Hz, 1H), 7.00 (t, *J* = 8.4 Hz, 2H).

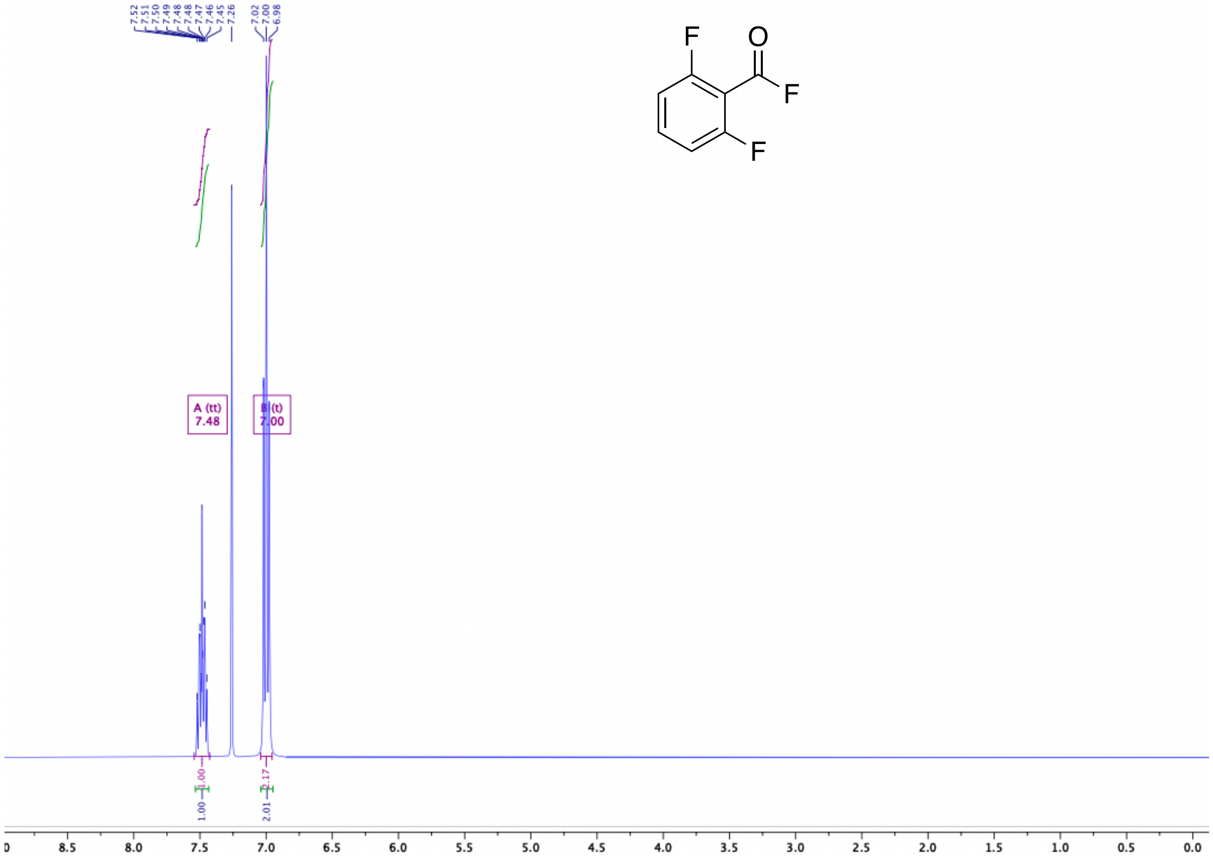

^{19}F NMR (377 MHz, Chloroform-*d*) δ -108.39 (d, *J* = 14.4 Hz).

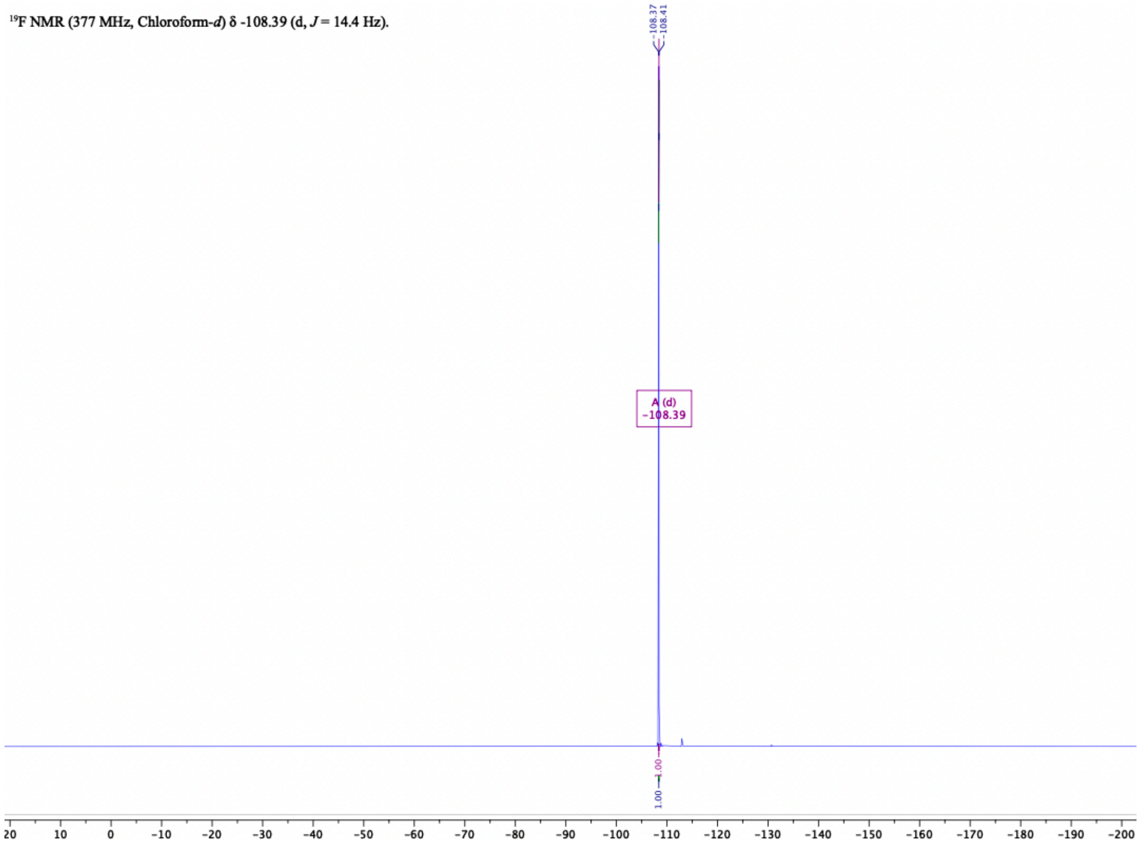

^{19}F NMR (376 MHz, Chloroform-*d*) δ 47.50 (t, *J* = 38.7 Hz).

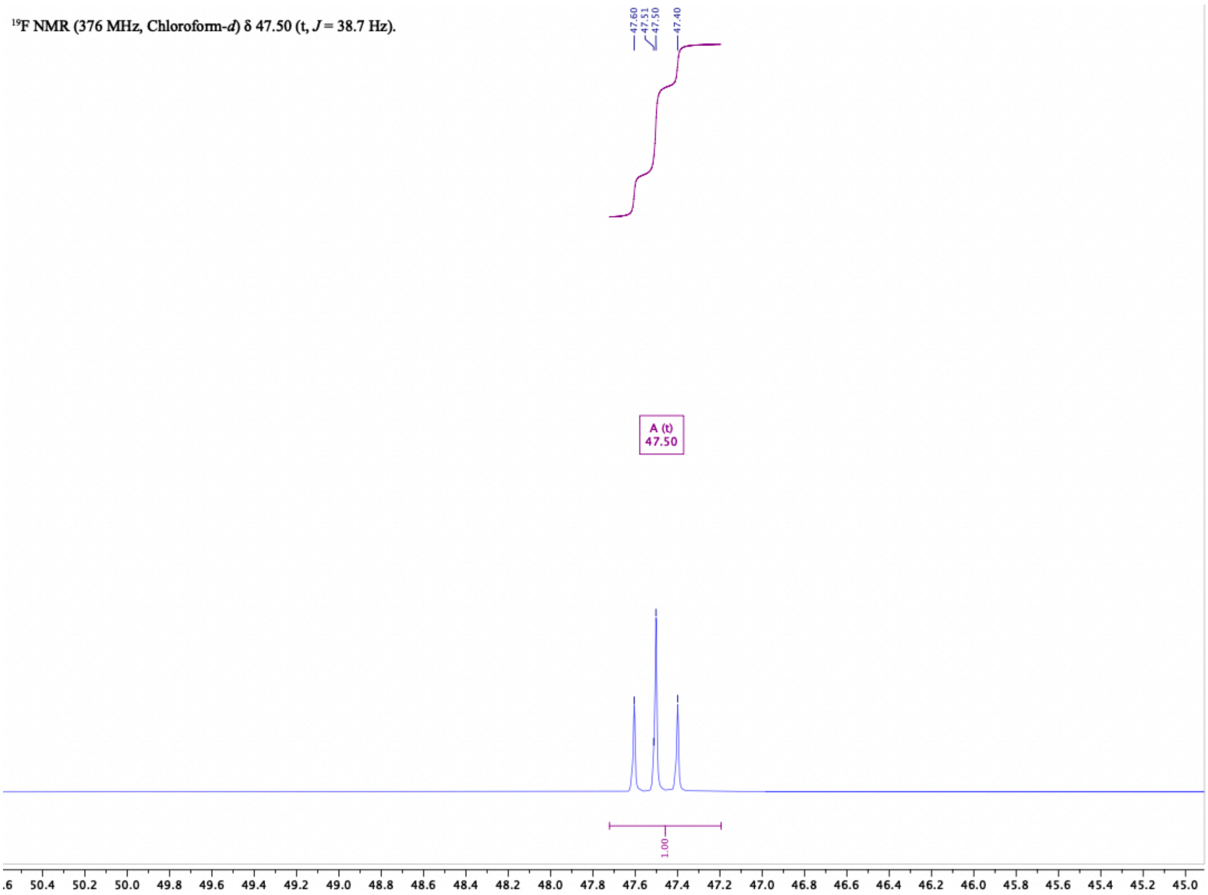

Figure S12. NMR spectra of 2,3,4,5,6-(pentafluoro)benzoyl fluoride.

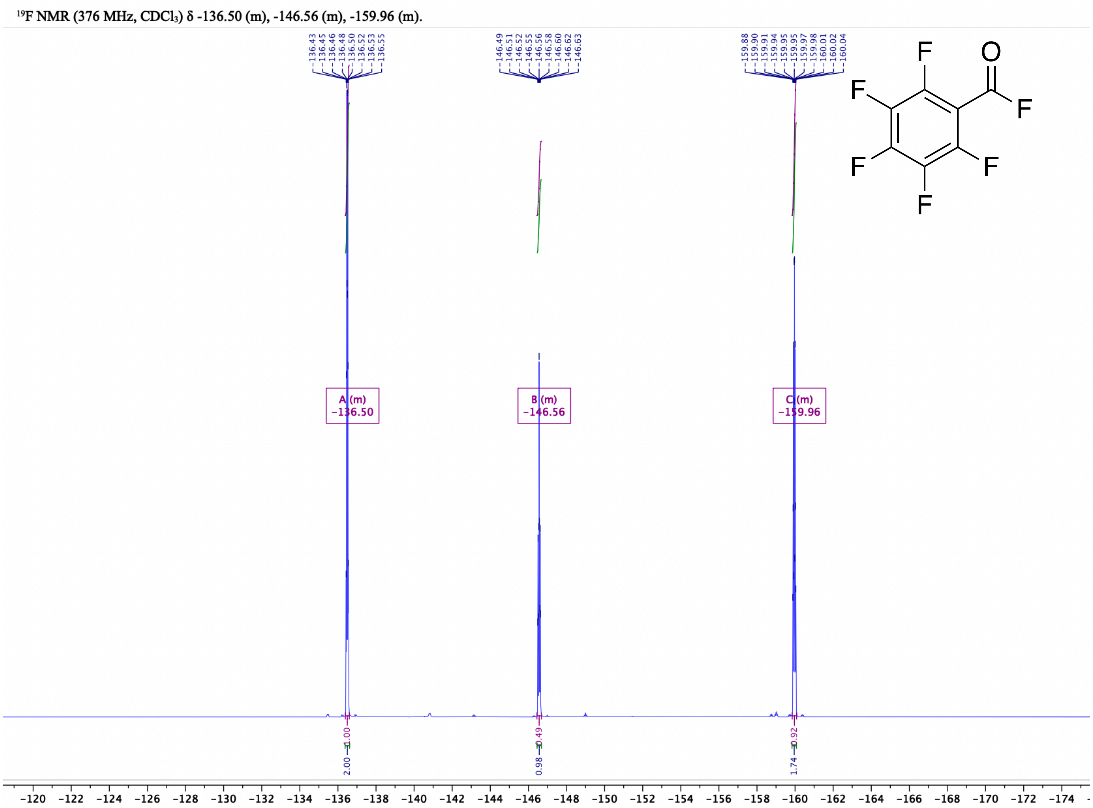

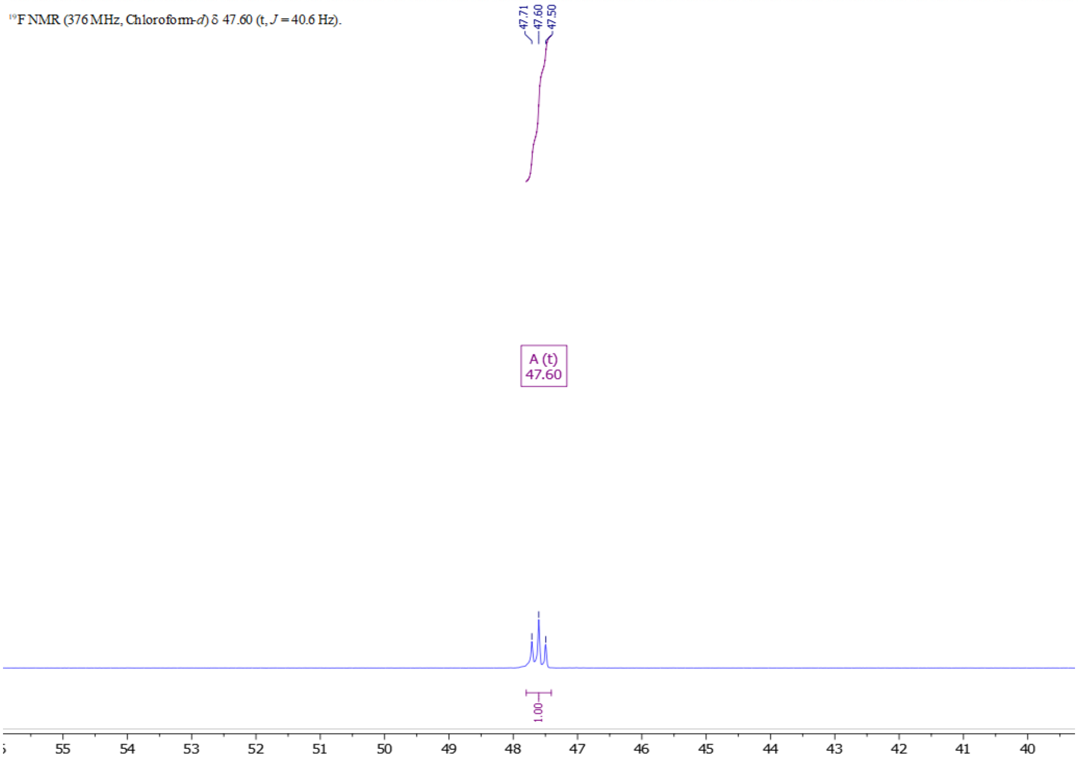

Figure S13. NMR spectra of 4-(trifluoromethyl)benzoyl fluoride.

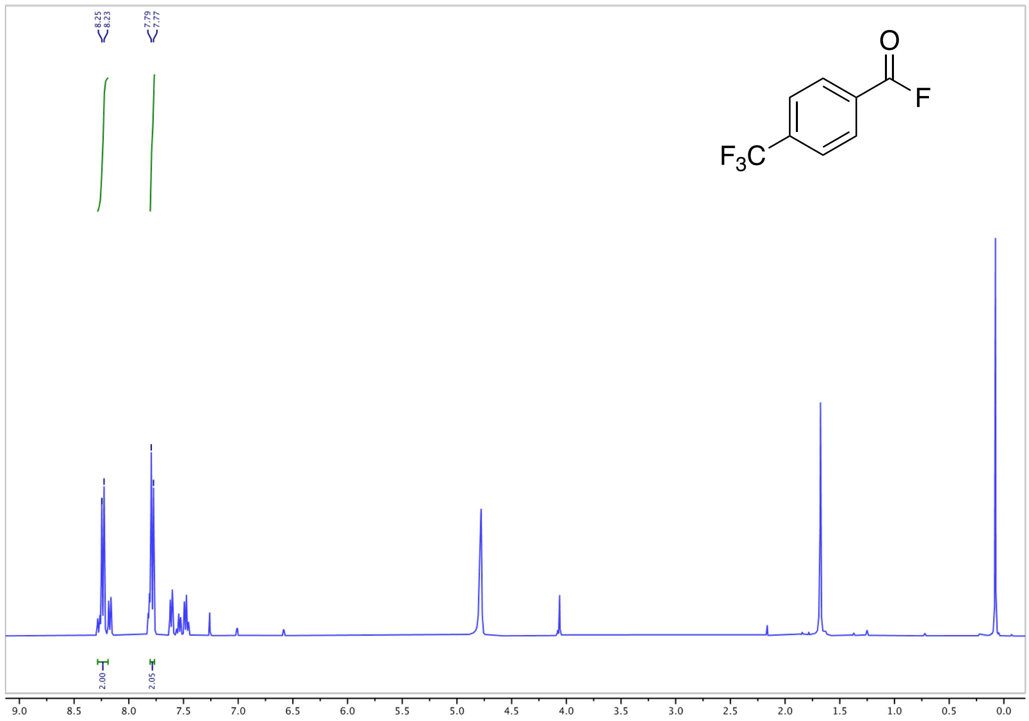

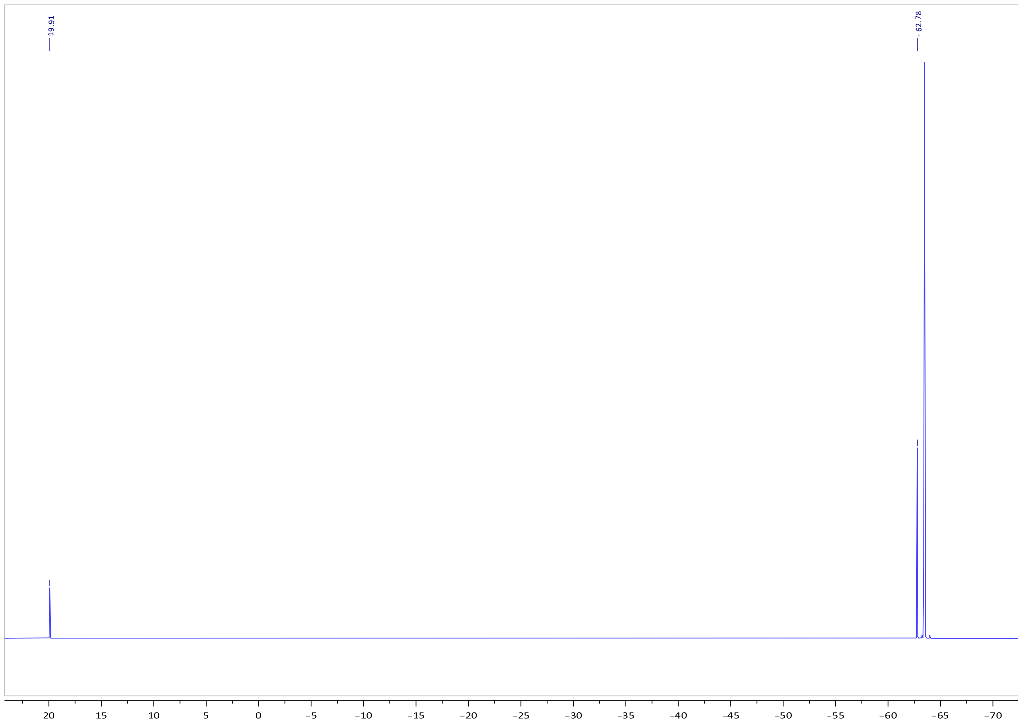

Crude ^{1}H and ^{19}F NMR spectra, including internal standard trifluorotoluene and catalyst.

Figure S14. NMR spectra of 4-ethylbenzoyl fluoride.

^{1}H NMR (400 MHz, $CDCl_3$) δ 7.95 (d, J = 8.3 Hz, 1H), 7.34 (d, J = 8.0 Hz, 1H), 2.74 (q, J = 7.7 Hz, 2H), 1.27 (t, J = 7.7 Hz, 2H).

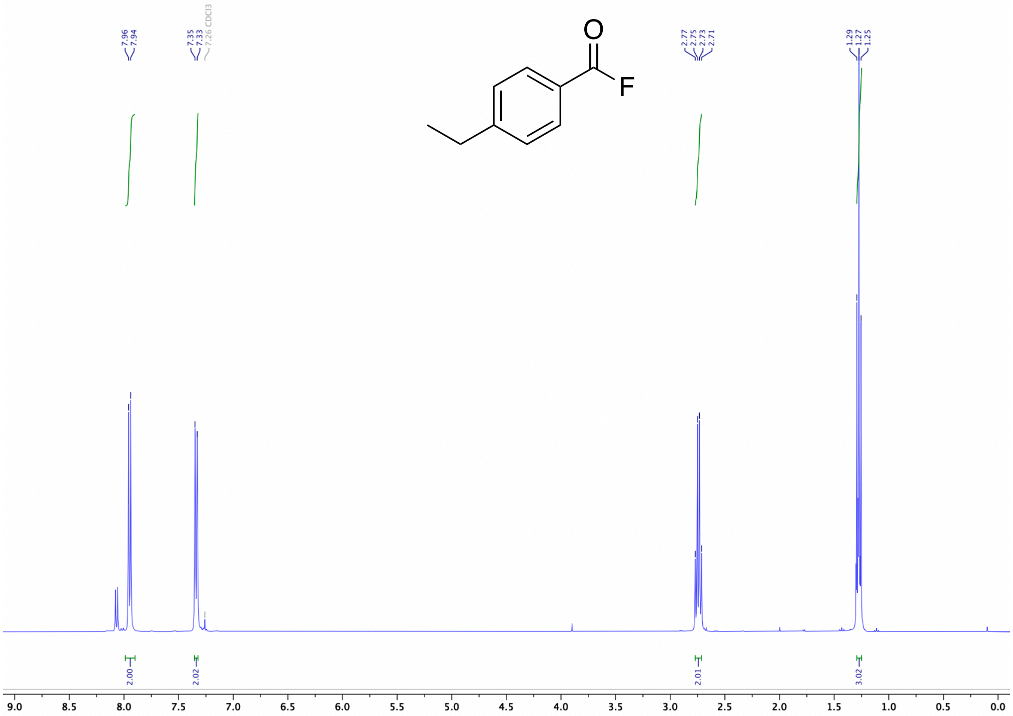

^{19}F NMR (376 MHz, $CDCl_3$) δ 17.44.

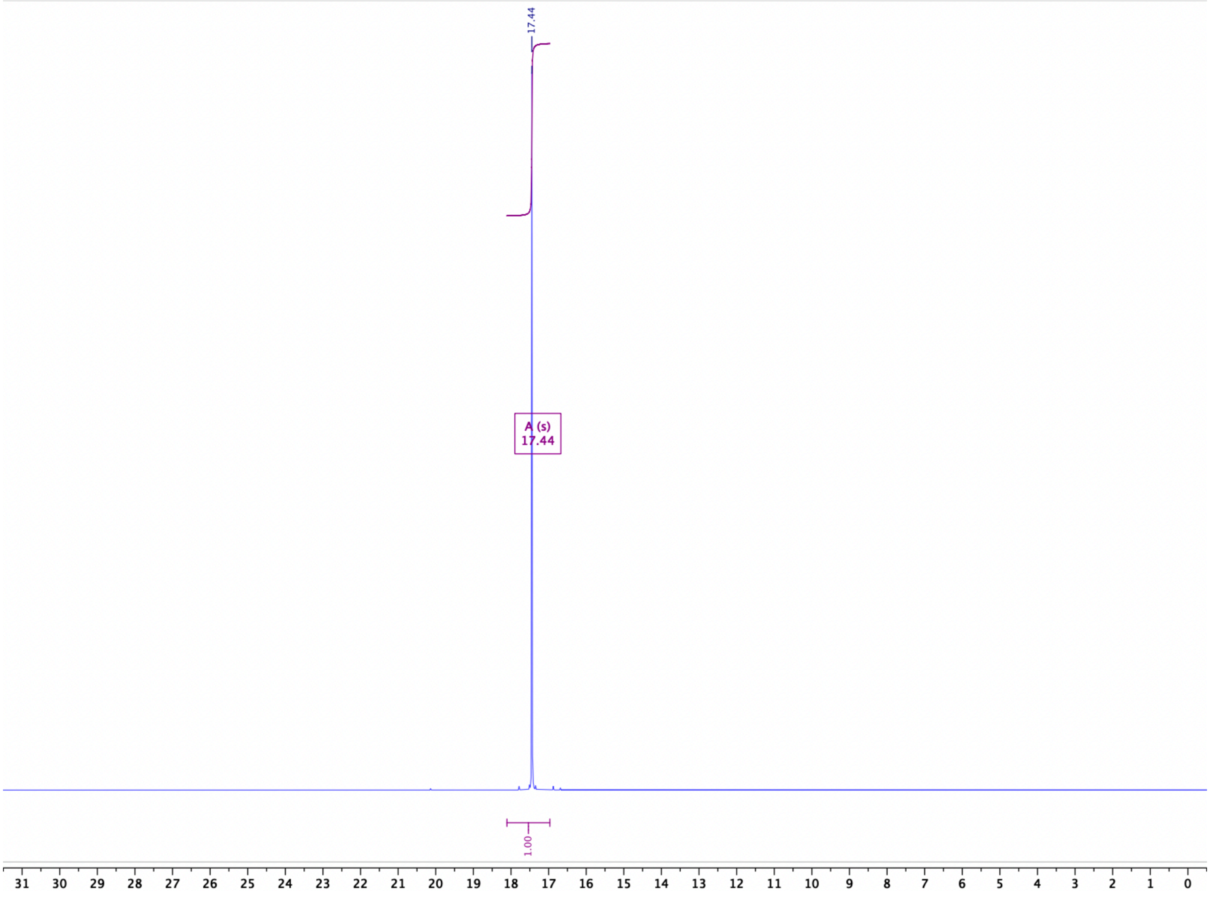

Figure S15. NMR spectra of 4-^{i}propylbenzoyl fluoride.

^{1}H NMR (500 MHz, Chloroform-d) δ 7.96 (d, J = 8.3 Hz, 2H), 7.37 (d, J = 7.9 Hz, 2H), 3.00 (h, J = 7.0 Hz, 1H), 1.28 (d, J = 6.9 Hz, 6H).

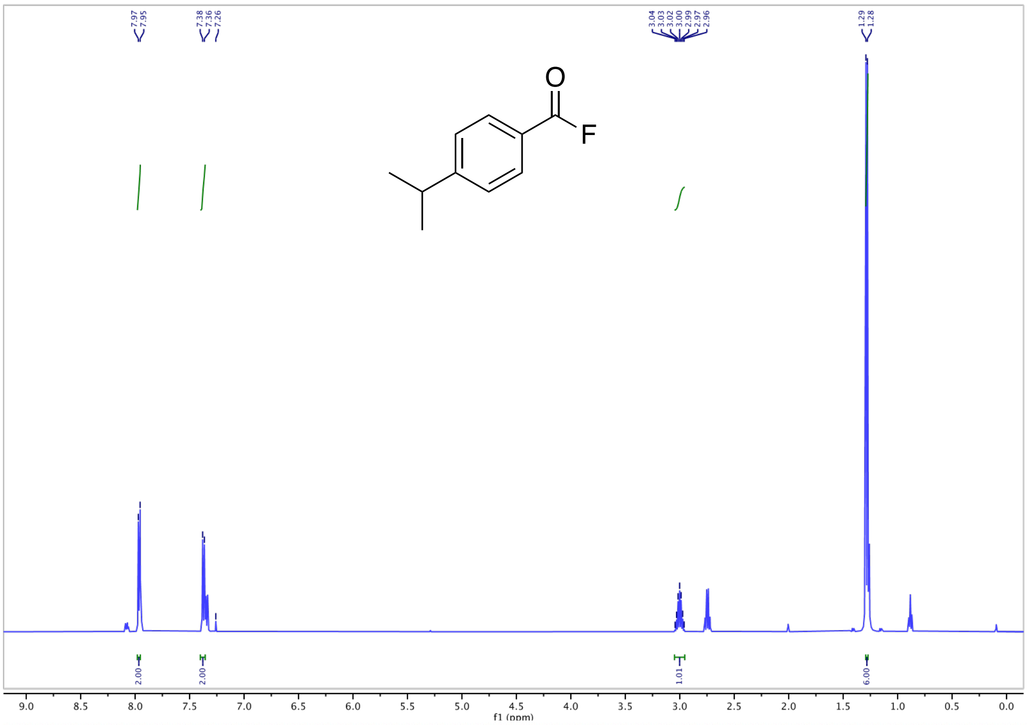

^{19}F NMR (376 MHz, $CDCl_3$) δ 17.53.

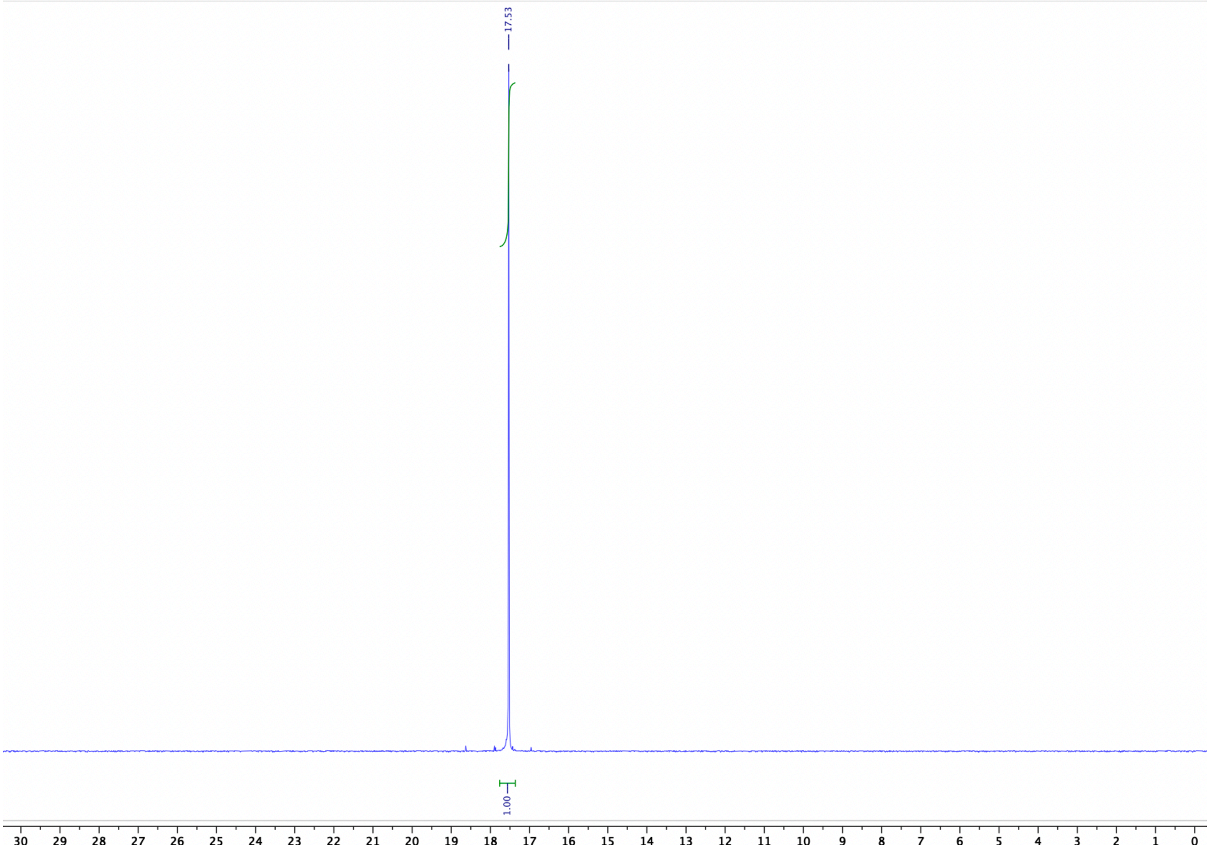

Figure S16. NMR spectra of 4-^{t}butylbenzoyl fluoride.

^{1}H NMR (400 MHz, Chloroform-*d*) δ 7.96 (d, *J* = 8.6 Hz, 2H), 7.54 (d, *J* = 7.6 Hz, 2H), 1.36 (d, *J* = 2.2 Hz, 9H).

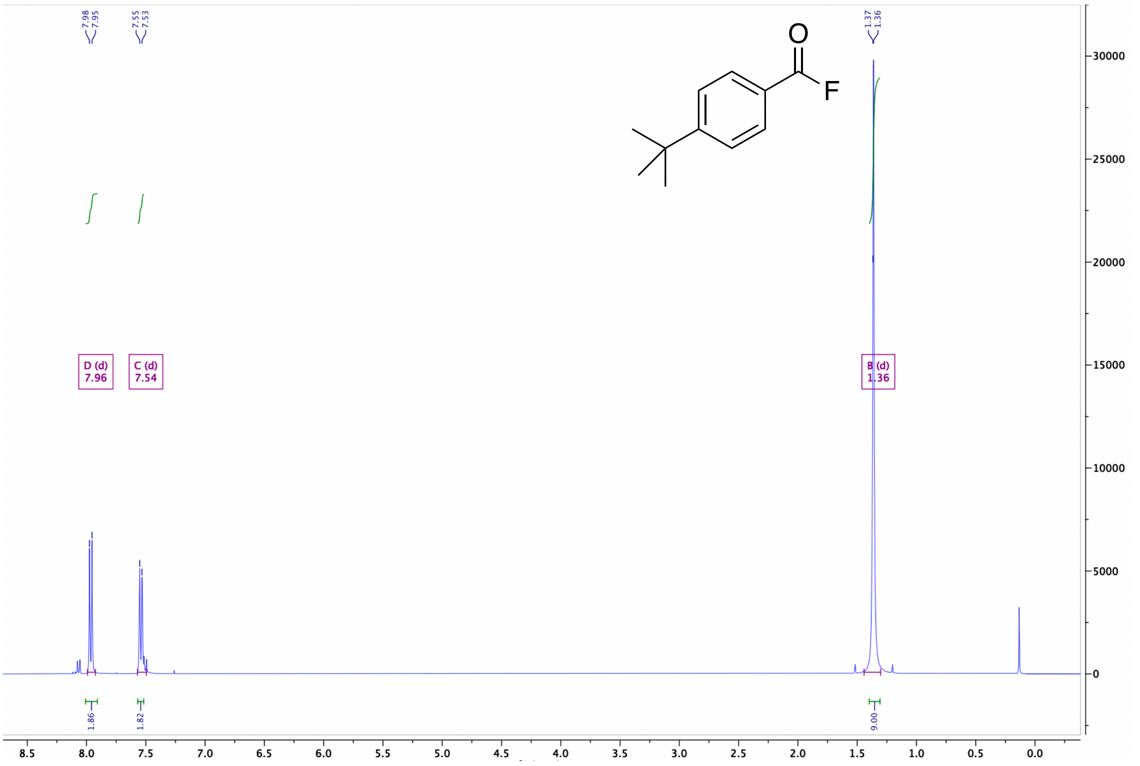

^{19}F NMR (377 MHz, $CDCl_3$) δ 17.62.

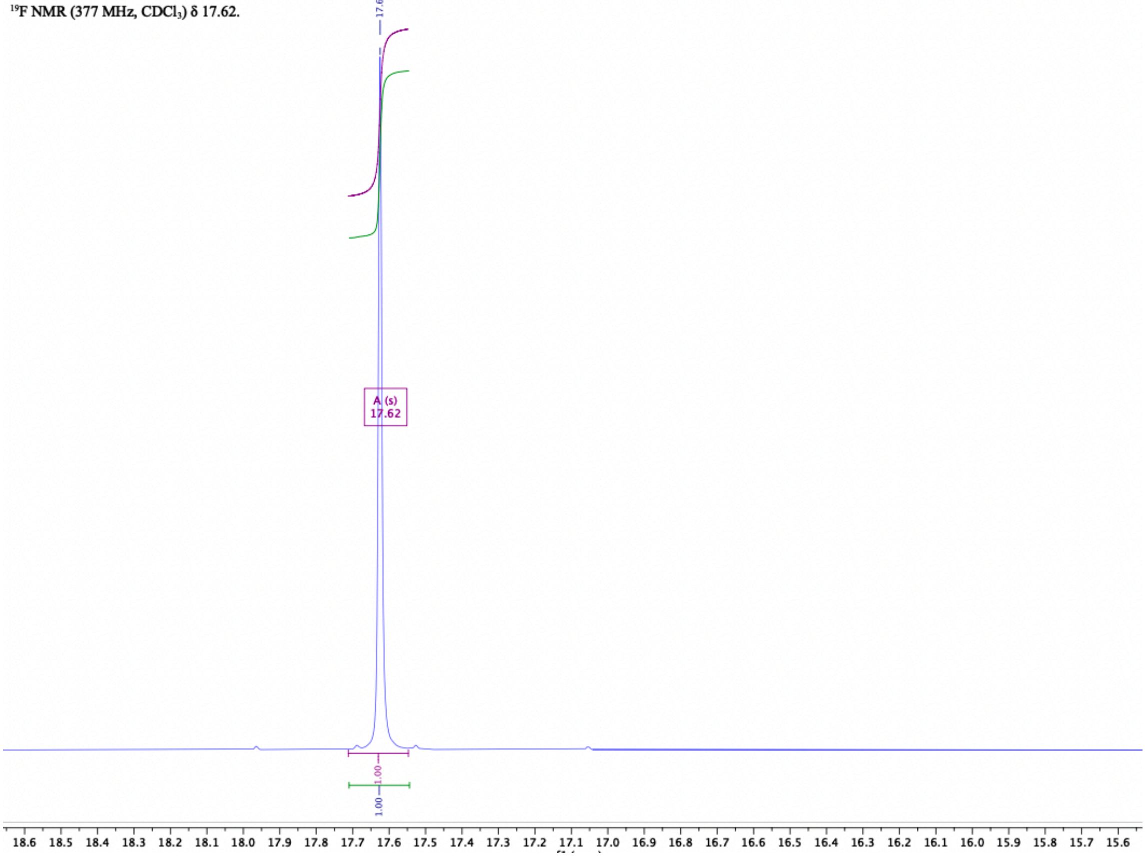

Figure S17. NMR spectra of 4-methoxybenzoyl fluoride.

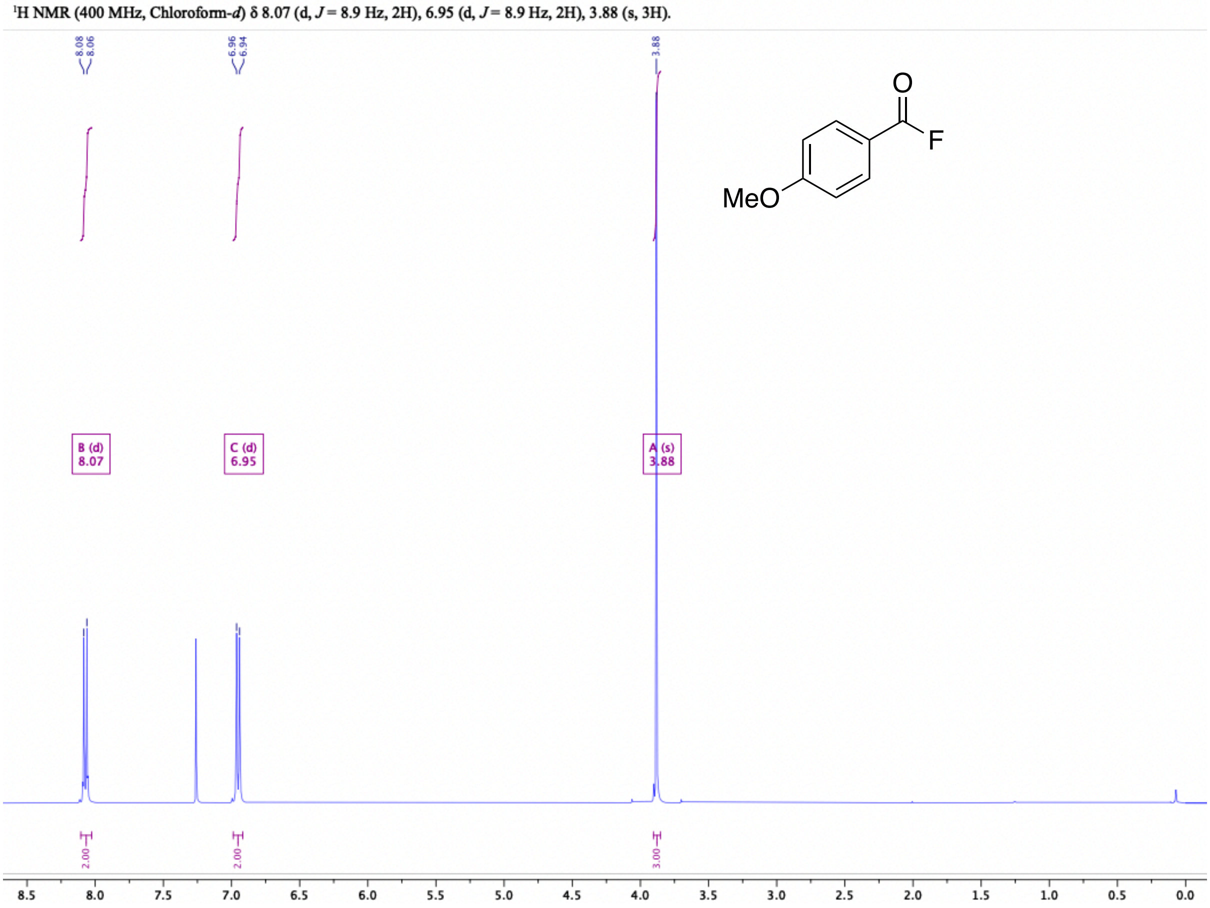

^{19}F NMR (376 MHz, $CDCl_3$) δ 15.89

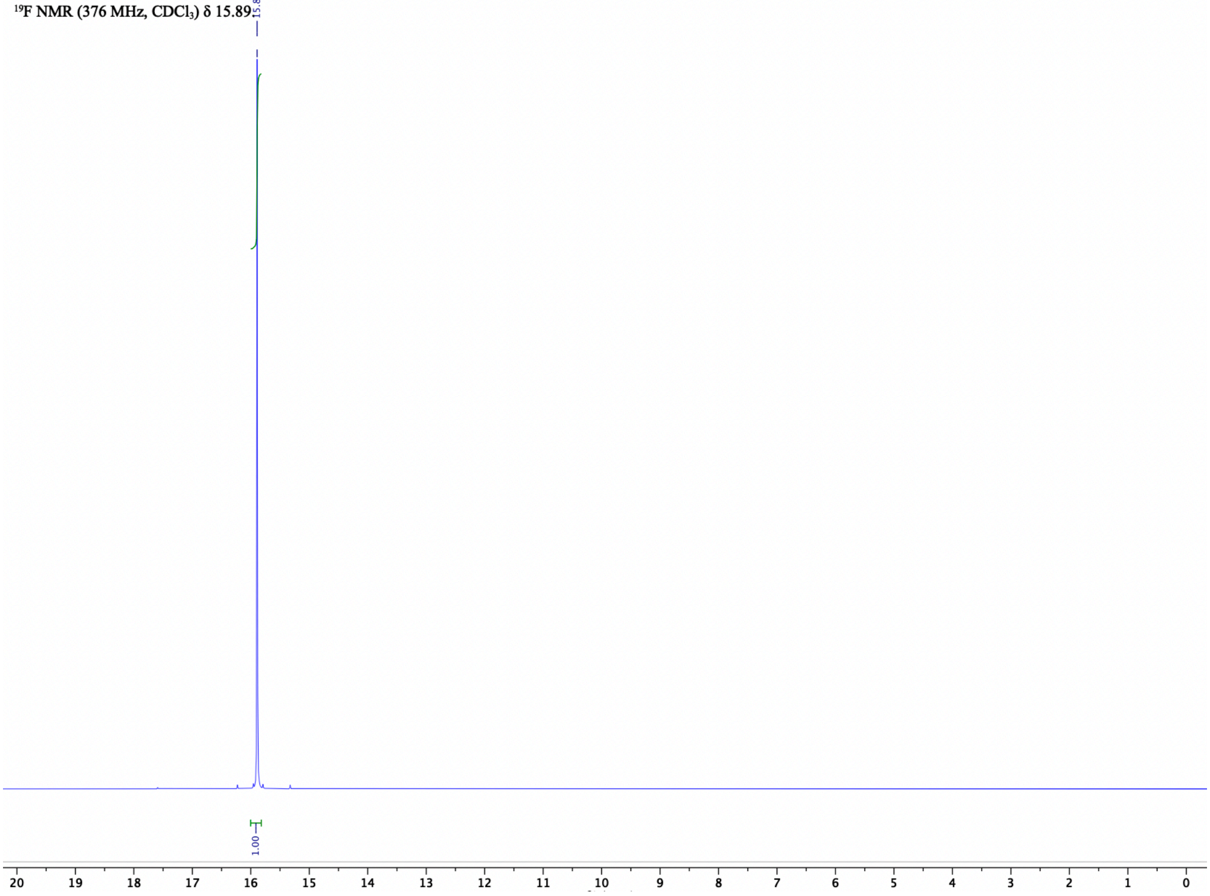

^{13}C NMR (101 MHz, Chloroform-*d*) δ 167.77 (d, *J* = 747.0 Hz), 132.37, 121.64, 113.77, 55.50.

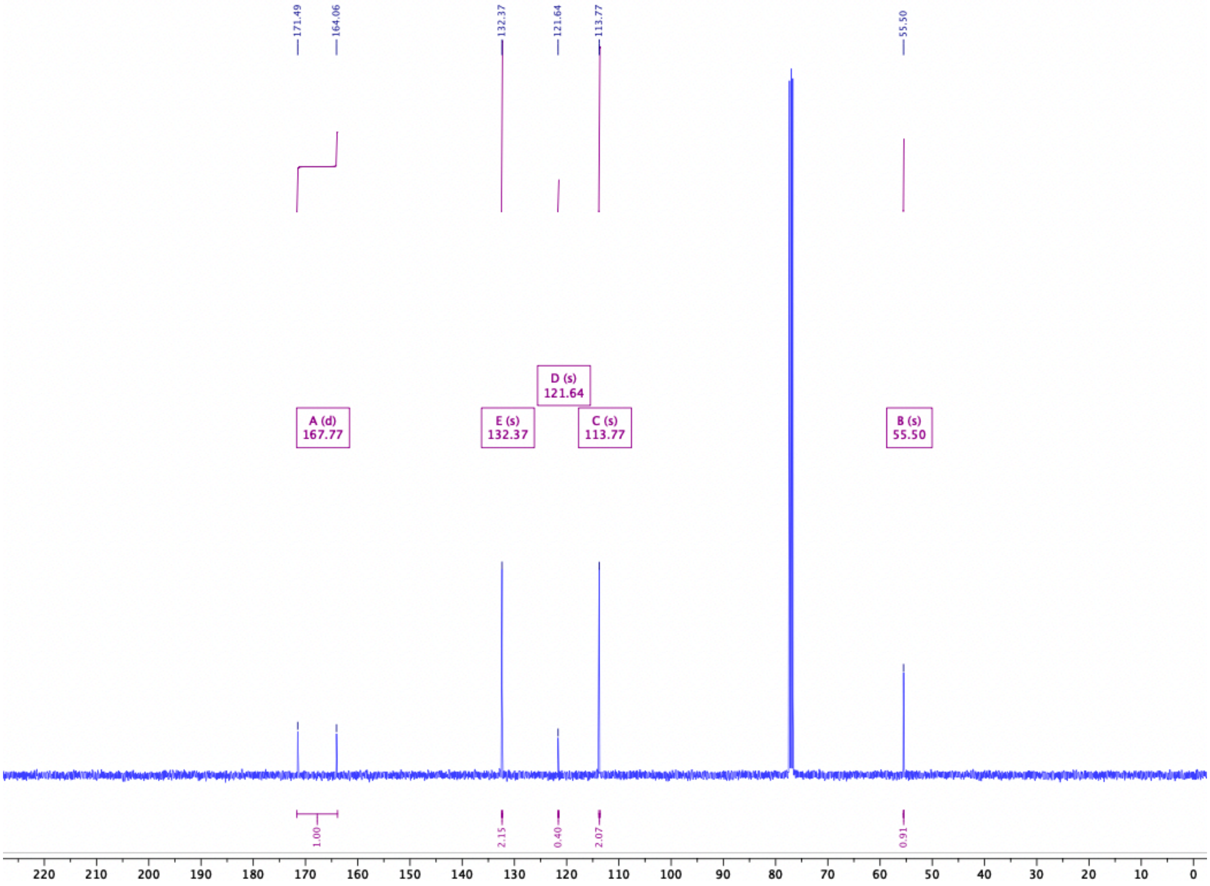

Figure S18. NMR spectra of probenecid fluoride.

^{1}H NMR (400 MHz, $CDCl_3$) δ 8.10 (dd, J = 8.7, 2.4 Hz, 2H), 7.89 (dd, J = 8.5, 2.3 Hz, 2H), 5.23 (s, 3H), 3.05 (tq, J = 6.9, 2.3 Hz, 4H), 1.48 (qt, J = 7.4, 2.3 Hz, 4H), 0.79 (tt, J = 7.5, 2.3 Hz, 6H).

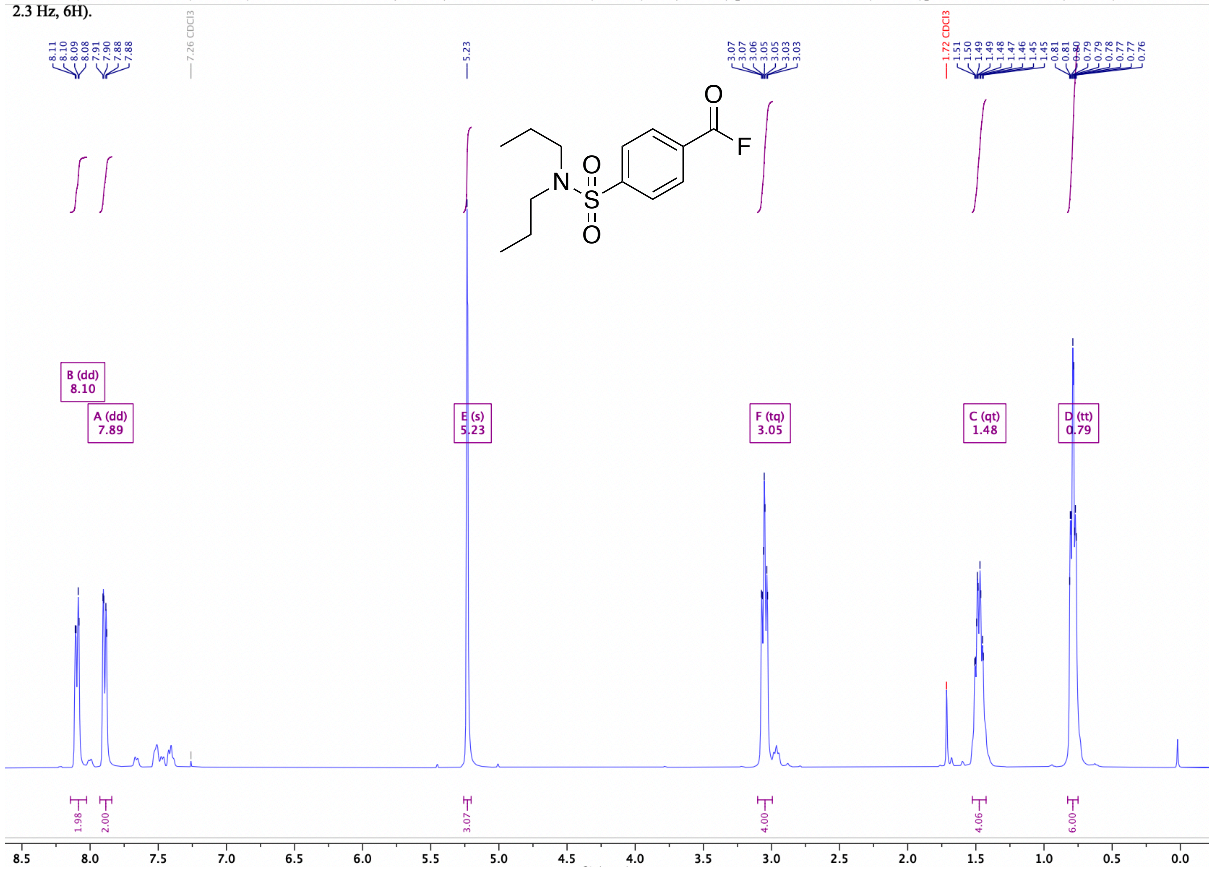

^{19}F NMR (377 MHz, $CDCl_3$) δ 19.91.

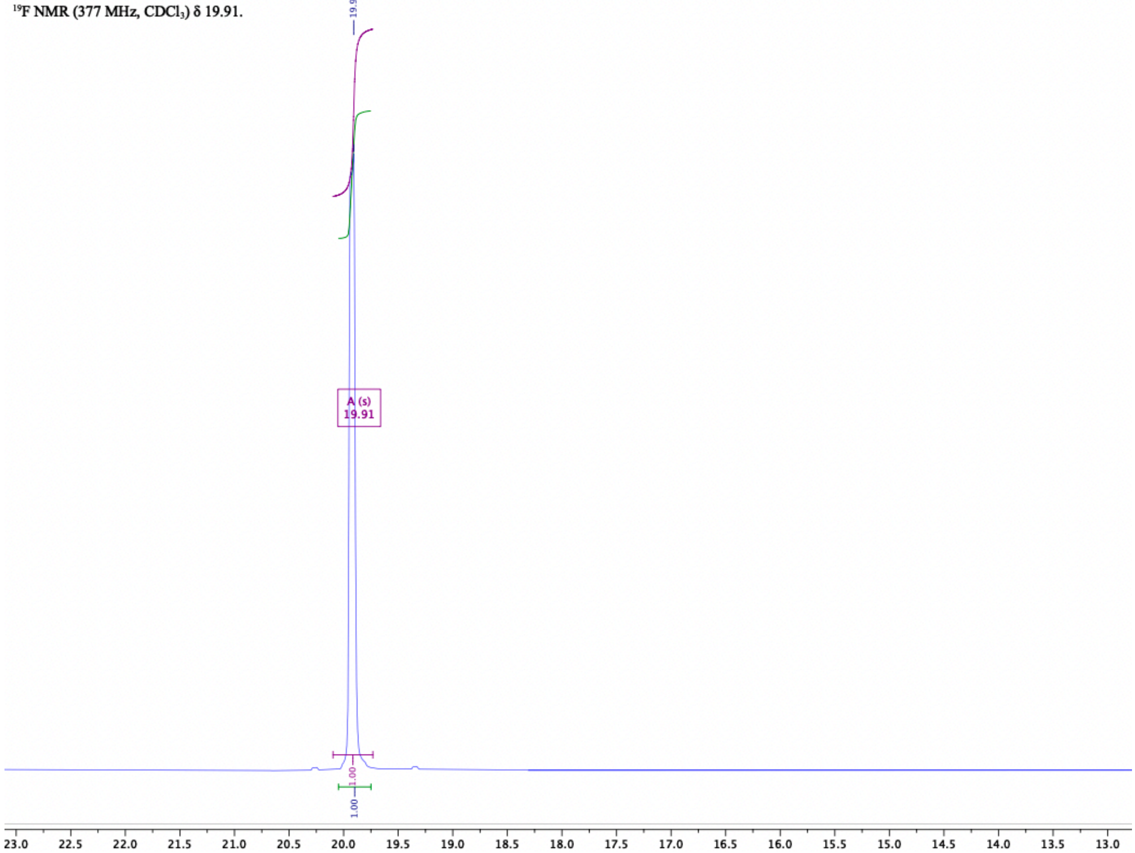

^{13}C NMR (101 MHz, $CDCl_3$) δ 155.94 (d, *J* = 345.6 Hz), 146.56, 131.95 (d, *J* = 3.7 Hz), 128.66, 127.44, 49.84, 21.80, 10.91.

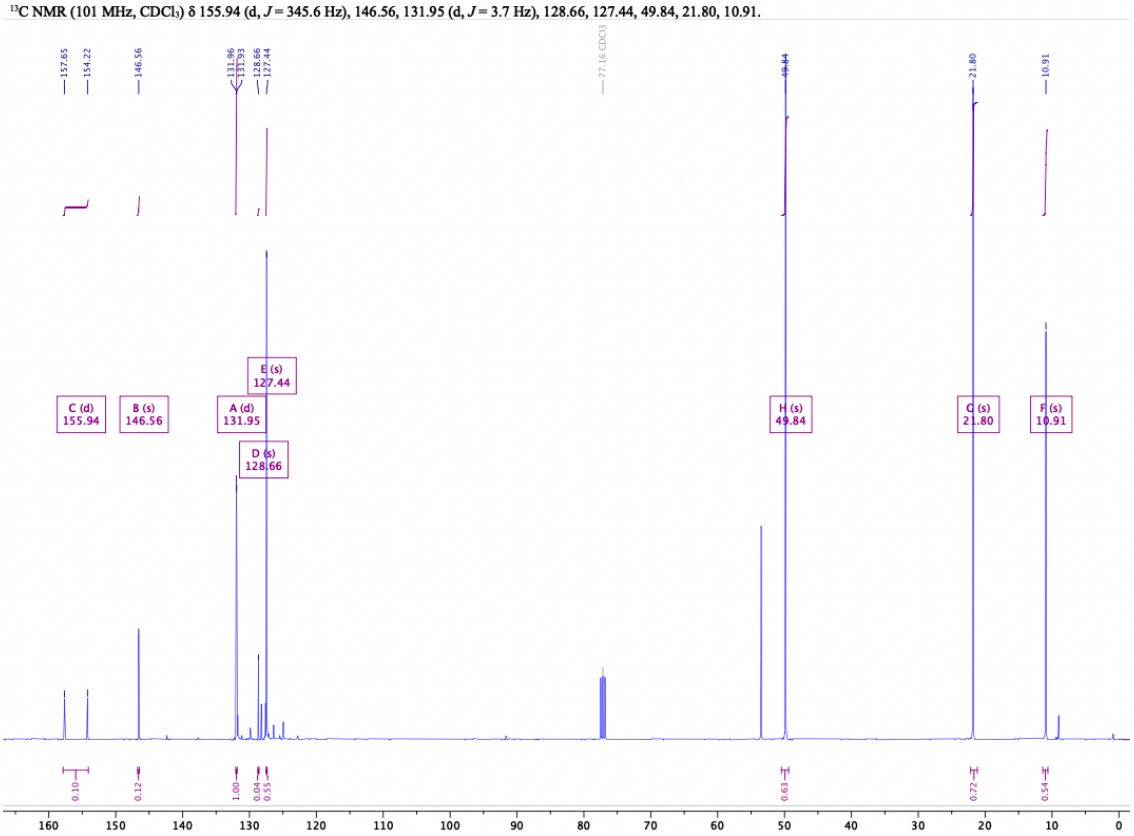

Crude ^{1}H & ^{13}C NMR with trifluorotouene internal standard and catalyst.
